# A reported 20-gene expression signature to predict lymph node-positive disease at radical cystectomy for muscle-invasive bladder cancer is clinically not applicable

**DOI:** 10.1371/journal.pone.0174039

**Published:** 2017-03-20

**Authors:** Kim E. M. van Kessel, Harmen J. G. van de Werken, Irene Lurkin, Angelique C. J. Ziel – van der Made, Ellen C. Zwarthoff, Joost L. Boormans

**Affiliations:** 1 Department of Pathology, Erasmus MC Cancer Institute, Erasmus Medical Center, Rotterdam, The Netherlands; 2 Department of Urology, Cancer Computational Biology Center, Erasmus MC Cancer Institute, Erasmus Medical Center, Rotterdam, The Netherlands; 3 Department of Urology, Erasmus MC Cancer Institute, Erasmus Medical Center, Rotterdam, The Netherlands; Medizinische Universitat Innsbruck, AUSTRIA

## Abstract

**Background:**

Neoadjuvant chemotherapy (NAC) for muscle-invasive bladder cancer (MIBC) provides a small but significant survival benefit. Nevertheless, controversies on applying NAC remain because the limited benefit must be weight against chemotherapy-related toxicity and the delay of definitive local treatment. Therefore, there is a clear clinical need for tools to guide treatment decisions on NAC in MIBC. Here, we aimed to validate a previously reported 20-gene expression signature that predicted lymph node-positive disease at radical cystectomy in clinically node-negative MIBC patients, which would be a justification for upfront chemotherapy.

**Methods:**

We studied diagnostic transurethral resection of bladder tumors (dTURBT) of 150 MIBC patients (urothelial carcinoma) who were subsequently treated by radical cystectomy and pelvic lymph node dissection. RNA was isolated and the expression level of the 20 genes was determined on a qRT-PCR platform. Normalized Ct values were used to calculate a risk score to predict the presence of node-positive disease. The Cancer Genome Atlas (TCGA) RNA expression data was analyzed to subsequently validate the results.

**Results:**

In a univariate regression analysis, none of the 20 genes significantly correlated with node-positive disease. The area under the curve of the risk score calculated by the 20-gene expression signature was 0.54 (95% Confidence Interval: 0.44-0.65) versus 0.67 for the model published by Smith *et al*. Node-negative patients had a significantly lower tumor grade at TURBT (p = 0.03), a lower pT stage (p<0.01) and less frequent lymphovascular invasion (13% versus 38%, p<0.01) at radical cystectomy than node-positive patients. In addition, in the TCGA data, none of the 20 genes was differentially expressed in node-negative versus node-positive patients.

**Conclusions:**

We conclude that a 20-gene expression signature developed for nodal staging of MIBC at radical cystectomy could not be validated on a qRT-PCR platform in a large cohort of dTURBT specimens.

## Introduction

Muscle-invasive bladder cancer (MIBC) comprises 20-25% of all newly diagnosed bladder cancers (urothelial carcinoma). Curative treatment options are few and survival is highly dependent on radical surgery and accompanying lymph node status. Patients having node-positive disease at radical cystectomy have a poor outcome. A large study on recurrence after cystectomy showed that around 80% of the cases with pathological node-positive disease (pN1-3) recurred versus only 20% of pathological node-negative organ-confined disease [1]. To eradicate micro metastases and thereby reducing the risk of node-positive disease at radical cystectomy, radical surgery can be preceded by cisplatin-based neoadjuvant chemotherapy (NAC). A clinical trial meta-analysis showed that about eight out of 100 patients with node-positive disease could benefit from upfront chemotherapy [2]. However, controversies on the use of NAC in MIBC remain because the survival benefit is small. A neoadjuvant regimen of cisplatin, methotrexate and vinblastine was shown to give a reduction in the risk of death (HR: 0.84) and an absolute increase in 10-year overall survival from 30% to 36% in a large multicenter randomized trial [3]. On the contrary, patients who do not respond to NAC experience a delay in time to curative surgery while being exposed to treatment-related toxicity. The limited survival benefit and the accompanying toxicities render many physicians reluctant to apply NAC for non-metastatic MIBC. As a result, the use of NAC in MIBC greatly varies across centers. Therefore, there is a clear clinical need for tools to guide treatment decisions on NAC in MIBC.

In other tumor types, several commercially available gene expression assays are used in clinical practice. In prostate cancer, Prolaris® and OncotypeDx Prostate® can be used for clinical decision making on surveillance of primary prostate cancer and Decipher® for clinical decisions on adjuvant therapy in surgically treated patients [4–6]. In breast cancer, assays such as OncotypeDx® and MammaPrint® have been clinically implemented [7]. In bladder cancer, a 12-gene expression signature to predict the outcome in non-muscle invasive bladder cancer was developed on a microarray platform and transferred to a qRT-PCR assay [8]. Although this 12-gene signature is still in need of further validation, it does suggest a potential role for gene expression assays in bladder cancer. Further, a 20-gene expression signature was developed to predict the presence of lymph node metastases at radical cystectomy in clinically node-negative MIBC patients. This signature might have the potential to be used to select patients for NAC [9]. Smith *et al* identified differentially expressed genes by microarray on 32 pairs of fresh frozen (FF) and formalin-fixed paraffin embedded (FFPE) tissues from three different cohorts of cystectomy specimens. Since NAC is administered before radical cystectomy, the selection for NAC would have to take place before cystectomy, i.e. after diagnostic transurethral resection of the primary bladder tumor (dTURBT). Further, to clinically implement such a signature a less complex laboratory assay on paraffin-embedded tissue samples is mandatory. Therefore, we aimed to validate this 20-gene expression signature on a qRT-PCR platform in a large cohort of 150 FFPE dTURBT specimens of MIBC patients who subsequently underwent radical cystectomy and pelvic lymph node dissection.

## Materials and methods

### Patient selection and data collection

**Fig 1:**
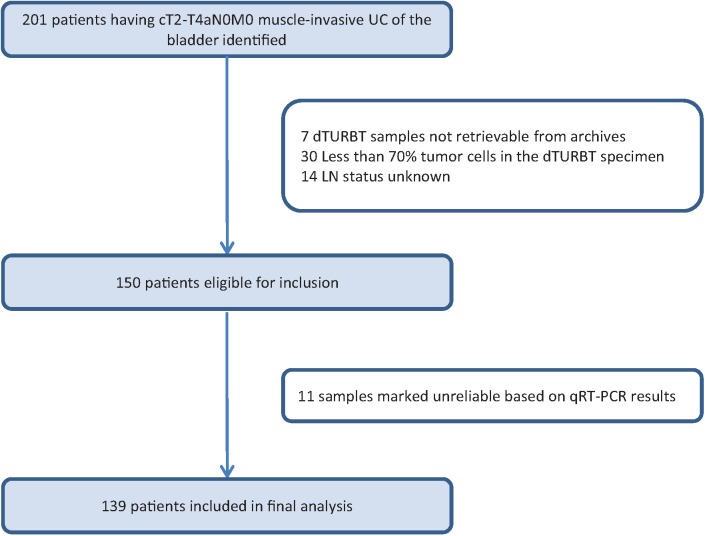
Flowchart of the selection of patients for the present study. UC: Urothelial Carcinoma, dTURBT: diagnostic transurethral resection of a bladder tumor, LN: lymph node.

This study was approved by the Erasmus MC institutional review board (MEC-2014-641), samples were collected and analyzed according to the code of conduct for responsible use of left over materials [10]. As part of standard procedure, all patients were informed and offered an option to opt out. Patients that opted out, by written or verbal notification, were excluded from the study. In total, 201 patients who were diagnosed with MIBC (urothelial carcinoma) and who were treated by radical cystectomy and pelvic lymph node dissection were retrospectively collected for the present study. None of the patients had received NAC. In seven patients, the FFPE blocks of the dTURBT could not be retrieved. Of the remaining 194 patients, 30 patients were excluded because the tumor area did not fulfill the minimum demand of at least 70% tumor cells or the RNA quality was insufficient to complete the analyses. Another 14 patients had to be excluded because the lymph node status at time of cystectomy could not be retrieved from the pathology reports. Therefore, 150 patients were included in the qRT-PCR analyses. Based on reliability criteria (see RNA expression data analysis) another 11 patients were excluded from the statistical analysis leaving 139 patients for the final analyses (Fig 1).

### RNA expression data

The 20 genes included in the assay were: *TOX3*, *SLC11A2*, *FAM36A/COX20*, *LIMCH1*, *RAB15*, *AVL9*, *PCMTD2*, *PTHLH*, *DPP4*, *PCDHGA10*, *MT1E*, *MAP4K4*, *SLC16A1*, *BST2*, *MMP14*, *IFI27*, *NCLN*, *HLA G*, *RRBP1* and *ICAM1*. The Taqman assays were chosen based on the Affimetrix probes used by Smith *et al* [9] and were selected by best coverage and exon spanning. First, the qRT-PCR of the 20 genes was optimized using cell line RNA (TCCSUP) and pooled FFPE derived tumor RNA by dilution series and calibration lines per gene. Then, all FFPE tumor samples (H&E slides) were centrally reviewed to select areas that contained at least 70% tumor cells. Of these tumor cell areas, a 2.2-mm core biopsy was taken (Beecher Instruments®, Silver Spring, MD, USA). The core was deparaffinized and RNA was isolated by High Pure FFPE RNA Micro Kit (Roche Applied Science®, Mannheim, Germany) according to the manufacturer’s protocol. The RNA concentration was measured using the Qubit RNA Assay (Invitrogen, Ltd, Paisley UK). Next, total RNA was reverse transcribed and cDNA was synthesized using a pool of 22 Gene Expression Taqman assays (20 genes + 2 housekeeping genes). The assay was done in two replicates for all samples. Then, 2 μl of the cDNA was pre-amplified using Pooled Gene Expression TaqMan assays and TaqMan PreAmp Master Mix (Applied Biosystems, Foster City, USA). Amplification was done in 15 cycles of 15 seconds at 95°C and 4 minutes at 60°C each. Pre-amplification was then followed by denaturation of 10 minutes at 99.9°C. Quantitative qRT-PCR was done in duplicate using the 7500 FAST Real Time PCR System (Applied Biosystems, Foster City, USA) including the preAmp cDNA, TaqMan Universal Master MIX II and single Gene Expression TaqMan assays (both Applied Biosystems, Foster City, USA). For normalization purposes, the housekeeping genes *HPRT*, *ACTB* and a plate control (T24 bladder cancer cell line RNA) was also included in the assay [11]. Two patient samples were run per plate, see for the plate design S1 Fig. The qRT-PCR was done under the following conditions: 10 minutes at 95°C, 40 cycles of 15 seconds at 95°C and 1 minute at 60°C. The threshold for determining the Ct value was set at 0.05. Since the amplification efficiency of the different assays was good, the comparative delta Ct method was used to quantify the gene expression levels.

### RNA expression data analysis

The qPCR data were analyzed and visualized using the R/Bioconductor package HtqPCR version 1.26.0 [12]. Sample data were split according to the qPCR plate design. A qPCR value was set to its replicated Ct-value if the qPCR result was undetermined. However, if both replicated Ct-values were undetermined both Ct-values were set to 35 and were marked as undetermined. The qPCR data were set as unreliable if the Ct value was outside a confidence interval of 95% within each transcript. Samples were discarded if the housekeeping genes *ACTB* and *HPRT* had two or more data points that were undetermined or unreliable.

Correlation plots were generated using the Pearson’s product-moment correlation coefficient values (*r*) and the subsequent hierarchical clustering was employed through complete linkage using the 1-*r* distances. Since *ACTB* expression analysis was the highest expressed gene and had the least missing values, all assays were normalized over the average *ACTB* Ct-value resulting in a ΔCt-value. Overall, the gene expression analysis was robust within each patient as depicted by the plots of the duplicate samples per patient (S2 Fig).

For each patient a risk score was calculated using the normalized Ct values based on the formula: the average ΔCt-value of the genes downregulated in lymph node-positive (LN+) tumors minus the average ΔCt-value of the genes upregulated in LN+ tumors according to Smith *et al* [9]. This formula was previously performed for validating microarray data using qPCR data [8]. Subsequently, using the risk score a logistic regression analysis was performed to predict the LN status. The area under the Receiver Operating Characteristic (ROC) curve was calculated and compared to the Area Under the Curve (AUC) reported by Smith *et al* [9].

The Cancer Genome Atlas (TCGA) clinical data and mRNA expression data was downloaded with CGDSR R package version 1.2.5 (https://github.com/cBioPortal/cgdsr) [13]. The lymph node-positive group was indicated by N1-3 and the lymph node-negative group by N0. The beeswarms were plotted with the R package beeswarm version 0.2.3 (https://github.com/aroneklund/beeswarm) and the Mann-Whitney *U* test was applied to compare lymph node-positive with lymph node-negative patients. Statistical analyses were performed using SPSS for Windows (IBM Corp. Released 2012. IBM SPSS Statistics for Windows, Version 21.0. Armonk, NY: IBM Corp) and R statistical software version 3.3.1 (R Core Team, 2016), which was also used for plotting the data.

## Results

### Patient and tumor characteristics

The clinical and histopathological characteristics of the 139 included patients are described in Table 1. The mean age was 64 years and 111 (80%) patients were male. None of the patient characteristics significantly differed between node-positive and node-negative patients. Node-negative patients had a significantly lower tumor grade at TURBT (p = 0.03), a significantly lower pT stage disease at radical cystectomy (p<0.01) and less frequent lymphovascular invasion at radical cystectomy than node-positive patients (13% versus 38%, p<0.01).

**Table 1:** Patient and tumor characteristics of all included patients with lymph node-negative or lymph node-positive disease (n = 139). The p-value indicates the (non)significant differences between both groups.

		LN negative (n = 91)	LN positive (n = 48)	p-value
**Patient characteristics**				
**Age**	Mean (range)	65 (32-83)	63 (37-81)	0.12
		**N (%)**	**N (%)**	
**Gender**	Male	74 (81)	37 (77)	0.35
	Female	17 (19)	11 (23)	
**Smoking**	Current	40 (44)	12 (25)	0.15
	Former	13 (14)	11 (23)	
	Never	12 (13)	7 (15)	
	*Unknown*	*26 (29)*	*18 (38)*	
**dTURBT characteristics**				
**Clinical Stage**	T2	91 (100)	47 (98)	0.35
	T3	-	1 (2)	
**Grade (WHO 1973)**	G2	8 (9)	2 (4)	**0.03**
	G3	74 (81)	46 (96)	
	Gx†	9 (10)	-	
**Concomitant *CIS***	Yes	16 (18)	14 (29)	0.49
	No	63 (70)	33 (69)	
	*Unknown*	*3 (3)*	*1 (2)*	
**Cystectomy characteristics**				
**Pathological Stage**	T0	11 (12)	1 (2)	**<0.01**
	Tis	2 (2)	-	
	T1	4 (4)	-	
	T2	20 (22)	7 (15)	
	T3	21 (23)	17 (35)	
	T4	8 (9)	6 (13)	
	Tx†	25 (28)	17 (35)	
**Grade (WHO 1973)**	G2	6 (7)	1 (2)	0.70
	G3	47 (52)	29 (60)	
	Gx†	38 (42)	18 (38)	
**Concomitant *CIS***	Yes	22 (24)	11 (23)	1.00
	No	11 (12)	5 (10)	
	*Unknown*	*58 (64)*	*32 (67)*	
**LVI**	Yes	12 (13)	18 (38)	**<0.01**
	No	14 (15)	2 (4)	
	*Unknown*	*65 (72)*	*28 (58)*	
**Total LN dissected**	Mean (range)	16 (2-40)	15 (2-33)	0.94
**Total LN positive**	Mean (range)	-	2 (1-14)	**<0.01**
**Sample characteristics**				
**RNA yield (ng/μl)**	Median (range)	606 (32-5360)	428 (44-3000)	0.81

LN: lymph nodes, CIS: *carcinoma-in-situ*, LVI: lymphovascular invasion

†: stage and/or grade not specified

The median RNA yield was similar for both node-negative and node-positive patients: 606 ng/μL (range 32-5360) versus 428 ng/μL (range 44-3000), p = 0.81).

### 20-gene expression signature validation

**Fig 2:**
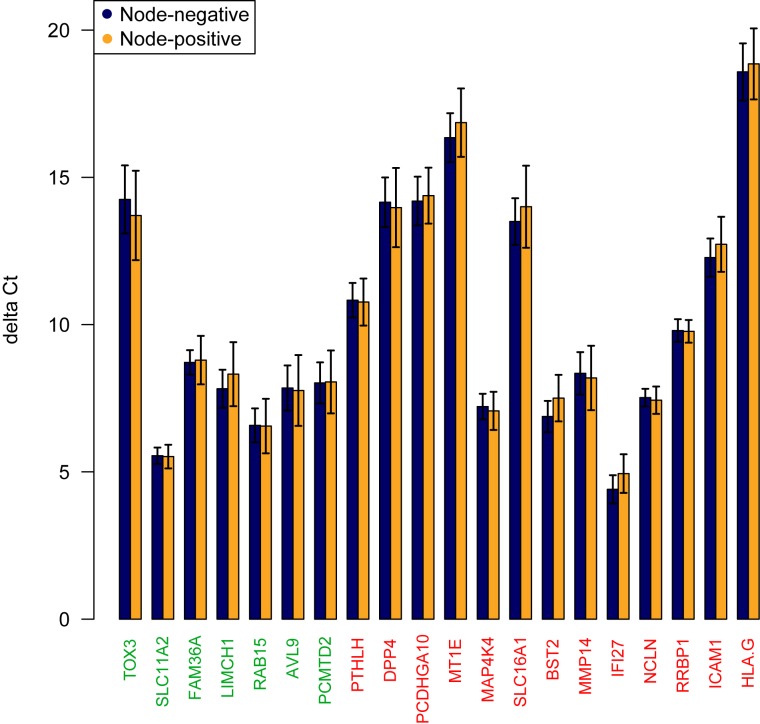
Risk score calculation, a barplot is showing the mean ΔCt values per gene in node-negative versus node-positive patients. Gene names colored red indicate higher expression in node-positive patients and gene names colored green indicate lower gene expression in node-positive patients according to Smith *et al* [9].

**Fig 3:**
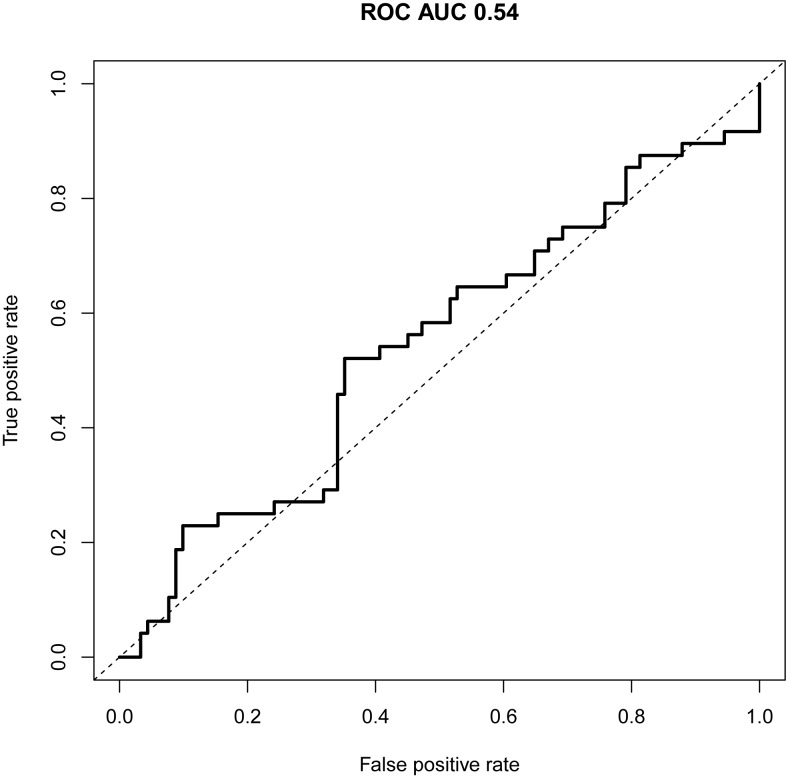
Model performance represented by the ROC curve. The predictive capacity of the 20-gene expression signature is represented by a ROC curve with an AUC of 0.54 (95% CI: 0.44-0.65).

After normalization, the mean expression level differences per gene for node-positive versus node-negative patients were plotted in a bar plot (Fig 2). The overall risk score per patient was calculated and a ROC curve was used to visualize the discriminatory value of the overall risk score. The predictive capacity of this risk score was minimal with an AUC of 0.54 (95% Confidence Interval (CI): 0.44-0.65, Fig 3). Of the 20 genes included in the model, normalized *BST2* and *IF127* ΔCt-values were the best performing individual predictors resulting in an odds ratio of 1.10 (95% CI: 0.96-1.25) and 1.11 (95% CI: 0.95-1.30) respectively (Table 2). Multivariate analysis of the ΔCt-values of these two genes combined resulted in an AUC of 0.59 (95% CI: 0.49-0.69).

**Table 2:** Univariate regression analysis of the ΔCt-values of the 20 single genes predicting node-positive disease at radical cystectomy (n = 139).

Gene	OR (95% CI)	p-value logistic regression
*TOX3*	0.98 (0.92-1.05)	0.57
*SLC11A2*	0.98 (0.76-1.28)	0.90
*FAM36A*	1.02 (0.87-1.18)	0.85
*LIMCH1*	1.05 (0.94-1.16)	0.40
*RAB15*	1.00 (0.88-1.13)	0.96
*AVL9*	0.99 (0.91-1.09)	0.90
*PCMTD2*	1.00 (0.91-1.11)	0.96
*PTHLH*	0.99 (0.87-1.13)	0.90
*DPP4*	0.99 (0.91-1.08)	0.81
*PCDHGA10*	1.01 (0.92-1.11)	0.78
*MT1E*	1.03 (0.95-1.13)	0.47
*MAP4K4*	0.97 (0.82-1.14)	0.70
*SLC16A1*	1.03 (0.95-1.12)	0.50
*BST2*	1.10 (0.96-1.25)	0.19
*MMP14*	0.99 (0.89-1.09)	0.80
*IFI27*	1.11 (0.95-1.30)	0.19
*NCLN*	0.96 (0.76-1.22)	0.74
*HLA_G*	1.01 (0.94-1.10)	0.73
*RRBP1*	0.99 (0.80-1.22)	0.93
*ICAM1*	1.05 (0.94-1.17)	0.42

**Fig 4:**
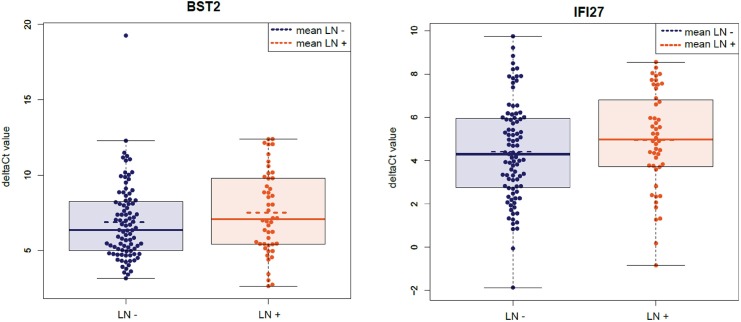
Beeswarm plots for BST2 and IF127 showing the distribution of ΔCt values for node-negative versus node-positive patient samples. The box indicates the upper and lower quartiles of distribution. The solid line indicates the median and the dotted line indicates the mean ΔCt value.

Beeswarm plots of the different genes showed the distribution of ΔCt-values for node-positive and node-negative samples per gene (*BST2* and *IF127* are shown in Fig 4, whereas all genes are shown in S3 Fig). Distribution of the ΔCt-values in these plots showed major overlap in all genes with some outliers. Mean and median results of the ΔCt-values showed similar trends in most genes (node-positive versus node-negative) with some exceptions (e.g. *TOX3*, median expression level higher in node-positive versus node-negative, mean expression level lower in node-positive versus node-negative).

**Fig 5:**
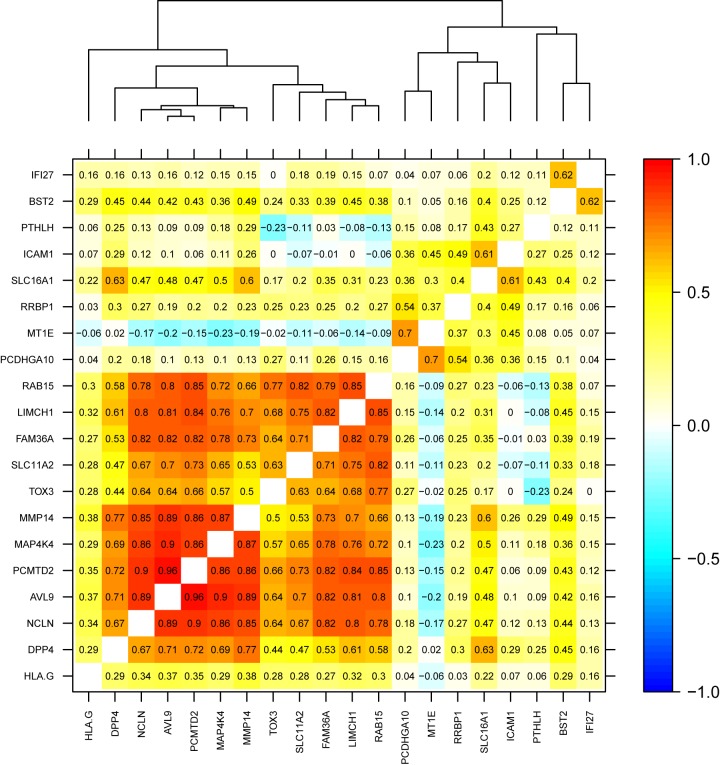
Pearson’s product moment correlation coefficient matrix showing a cluster of eight highly positively correlated genes (*AVL9*, *PCMTD2*, *FAM36A*, *LIMCH1*, *RAB15*, *NCLN*, *MAP4K4*, *MMP14*) (*r* = 0.66–0.96).

Pearson correlation analysis showed significant positive correlation in a cluster of eight genes (Fig 5). Of these eight genes, five were determined as upregulated (*AVL9*, *PCMTD2*, *FAM36A*, *LIMCH1*, *RAB15*) in node-positive disease and three were determined as downregulated (*NCLN*, *MAP4K4*, *MMP14*) in node-positive disease by Smith *et al* [9].

### 20-gene expression signature and validation in TCGA

The TCGA gene expression data by RNA sequencing from 408 chemo-naïve cystectomy specimens is publicly available (12) and was used as an additional external validation cohort. All 20 genes from the 20-gene expression signature and necessary clinical data were available for 365 MIBC samples. Beeswarm plots of the 20 genes of interest showed a similar overlap in data points (S4 Fig). Gene expression levels did not differ significantly between node-positive and node-negative patients for any of the genes (Mann Whitney-*U*-test, range p-values 0.07-0.93, S4 Fig). Pearson correlation analysis did not convincingly substantiate the positive correlations in the eight genes found in our data (except *LIMCH1* and *RAB15*). It did show some correlation between a number of other genes (e.g. *ICAM*, *DPP4*. *TOX3*) (S5 Fig).

### Technical evaluation

Since there were no major differences in patient and tumor characteristics between node-negative and node-positive patients, we checked the data for possible technical confounders that might have influenced the gene expression levels. We hypothesized that the originating hospital might have influenced the RNA quantity and quality of the samples, for instance by differences in fixation protocols. Since most samples originated from Erasmus MC (35%), we repeated the ROC analysis for Erasmus MC archival material only (n = 37 node-negative and n = 11 node-positive) as an attempt to rule out this bias. The predictive capacity was marginally poorer in this more homogeneous but significantly smaller sample set (AUC 0.52, 95% CI: 0.31-0.72).

## Discussion

Curative treatment options for MIBC are few and survival is highly dependent on radical surgery and lymph node status. The presence of micro-metastasis in lymph nodes could be an additional argument to justify the administration of NAC before radical surgery. There is a clear clinical need to have tools that could aid in the decision whether or not to give NAC. In the present study, we aimed to validate a 20-gene expression signature, which previously was shown to predict the presence of lymph node metastases at cystectomy in clinically node-negative MIBC patients [9].

Since the dTURBT represents the starting point for treatment decisions in MIBC patients, we used RNA isolated from a large cohort of dTURBT and not radical cystectomy specimens. In addition, we aimed to validate the 20-gene expression signature using qRT-PCR, which is an easier to use technology than microarray analysis and is being used in many pathology laboratories around the world. Our data showed robust performance of the qRT-PCR performed: 1) the dilution series and calibration plots in the optimization of the qRT-PCR showed a good quality in the FFPE-derived RNA samples; 2) the gene plots of the duplicate samples per patient also showed a near optimal result as robust measurements in the RT-qPCR. Nevertheless, we were unable to validate the predictive capacity of the previously published 20-gene expression signature. Smith *et al* who obtained the 20-gene expression signature and accompanying cut-offs by optimization in two independent training cohorts of archival FFPE cystectomy specimens and one additional test cohort of 185 cystectomy specimens described an AUC of 0.67 (95% CI: 0.60-0.75) [9]. In contrast, our expression model only reached an AUC of 0.54 (95% CI: 0.44-0.65). In addition, analysis of the TCGA expression data in 365 MIBC patients also did not show a difference between node-positive and node-negative patients based on the suggested 20 genes.

Recently, Seiler *et al* published a study that attempted to design another gene expression signature to predict the presence of lymph node metastases at time of cystectomy [14]. Seiler *et al* used RNA isolated from cystectomy specimens for a whole transcriptome approach in order to include also non-coding transcripts. This approach resulted in a 51 K-nearest neighbor classifier (KNN51). Of the 51 genes included in this signature, none overlapped with the 20 genes from the 20-gene expression signature of Smith *et al* [9, 14]. However, Seiler *et al* did attempt to validate the 20-gene expression signature. The 20-gene expression signature had an AUC of 0.46 and a non-significant OR of 1.39 versus an AUC of 0.82 and an OR of 2.65 (p<0.01) for the KNN51 model [14]. A number of important differences should be noted between the study of Seiler *et al* and our study. The platform used by Seiler *et al* was whole transcriptome expression analysis, a laborious technique that is not widely available for standard diagnostic procedures in contrast to the qRT-PCR technology we used. Further, Seiler *et al* like Smith *et al* used radical cystectomy for their analysis and not dTURBT specimens we used. As the authors stated in their conclusions, the KNN51 expression signature should also first be validated on dTURBT specimens before implementation in clinical practice is possible.

In general, the discordance between the study by Smith *et al* and our current study does not undermine the value of the primary study, given the different methodologies. However, we believe it does illustrate the practical limitations and the difficulties related to translation into the clinical setting. Several other hypotheses could be raised for the lack of validation of the predictive gene expression model in our study. In general, MIBC is known to be a heterogeneous cancer in terms of biological behavior. Even in similar histological subtypes, the biological profile of the tumor may differ greatly. Recent publications identified the presence of very distinct molecular subtypes in MIBC having different phenotypes and accompanying biological behavior [15, 16]. These molecular subtypes might be differently distributed among the different datasets that were used to develop and validate the 20-gene expression signature. Secondly, the punches taken from the dTURBT samples might not have portrayed the overall tumor expression levels due to intra-tumor heterogeneity. Also, the punch taken from the FFPE TURBT material could have been contaminated by normal cells. We cannot fully exclude this possibility, even though we carefully selected areas of at least 70% tumor cells and we punched centrally in the tumor sections. Thirdly, from a statistical point of view, robustness of the predictive model would increase if gene expression could be defined as either ‘on’ or ‘off’. The subtle differences in higher or lower expression of certain genes in node-positive versus node-negative samples probably led to part of the frailty of the model. Lastly, correlation between different genes in this expression signature could suggest a biological interaction, such as cross-communicating pathways. Even though we could not confirm this finding in the TCGA data and this data was not available for the model presented by Smith *et al*. Multi-collinearity between different components of a predictive model risks overestimation of the predictive capacity of a model and should therefore be corrected for in the development process.

The present study showed the difficulty of validating gene expression data, not only in an independent patient cohort but also on a different platform. In line with the whole transcriptome approach by Seiler *et al* on 199 patients and the RNA sequencing data of 365 patients of TCGA, we were unable to validate the 20-gene signature on a qRT-PCR platform in 139 MIBC patients who underwent dTURBT. We therefore conclude that a previously developed 20-gene expression signature that predicted the presence of lymph node metastases at pelvic lymph node dissection and radical cystectomy in MIBC patients who were clinically node-negative is clinically not applicable and cannot serve as a tool to guide treatment decisions on NAC in MIBC patients.

## Supporting information

S1 Fig Plate-design of the qRT-PCR assay validating the 20-gene expression signature. Two patients were run per plate in duplicate. For normalization purposes the housekeeping genes *HPRT*, *ACTB* and a plate control (T24 bladder cancer cell line RNA) was also included in the assay.
(PDF)

S2 Fig Plots of the Ct values for the 20 genes and household genes of the duplicate samples per patient. (PDF)

S3 Fig Beeswarm plots of the 20 different genes and 2 household genes, which show the distribution of ΔCt values for node-positive and node-negative samples per gene (n = 139). The box indicates the upper and lower quartiles of distribution, with the solid line indicating the median and the dotted line indicating the mean ΔCt value.
(PDF)

S4 Fig Beeswarm plots based on TCGA data (n = 365) of the distribution of ΔCt values for the 20 different genes in node-positive and node-negative samples per gene. The box indicates the upper and lower quartiles of distribution, with the solid line indicating the median and the dotted line indicating the mean ΔCt value.
(PDF)

S5 Fig Pearson’s product moment correlation coefficient matrix based on the TCGA RNA expression data of the 20 genes of interest (n = 408). (PDF)
