# Supplementary material for: A reported 20-gene expression signature to predict lymph node-positive disease at radical cystectomy for muscle-invasive bladder cancer is clinically not applicable
Source: PLoS One. 2017 Mar 20;12(3):e0174039. doi: 10.1371/journal.pone.0174039 (PMC5358850; doi:10.1371/journal.pone.0174039)
Supplement: S1 Fig — Two patients were run per plate in duplicate. For normalization purposes the housekeeping genes HPRT, ACTB and a plate control (T24 bladder cancer cell line RNA) was also included in the assay. (PDF) [file pone.0174039.s001.pdf]

	1	2	3	4	5	6	7	8	9	10	11	12	sample
**A**	SLC11A2	COX20	TOX3	LIMCH1	RAB15	AVL9	PCMTD2	PTHLH	DPP4	PCDHGA10	MT1E	MAP4K4	**1a**
**B**	SLC16A1	BST2	MMP14	IFI27	NCLN	HLA-G	RRBP1	ICAM1	ACTB	HPRT			**1a**
**C**	SLC11A2	COX20	TOX3	LIMCH1	RAB15	AVL9	PCMTD2	PTHLH	DPP4	PCDHGA10	MT1E	MAP4K4	**1b**
**D**	SLC16A1	BST2	MMP14	IFI27	NCLN	HLA-G	RRBP1	ICAM1	ACTB	HPRT	T24 HPRT	nt HPRT	**1b**
**E**	SLC11A2	COX20	TOX3	LIMCH1	RAB15	AVL9	PCMTD2	PTHLH	DPP4	PCDHGA10	MT1E	MAP4K4	**2a**
**F**	SLC16A1	BST2	MMP14	IFI27	NCLN	HLA-G	RRBP1	ICAM1	ACTB	HPRT			**2a**
**G**	SLC11A2	COX20	TOX3	LIMCH1	RAB15	AVL9	PCMTD2	PTHLH	DPP4	PCDHGA10	MT1E	MAP4K4	**2b**
**H**	SLC16A1	BST2	MMP14	IFI27	NCLN	HLA-G	RRBP1	ICAM1	ACTB	HPRT	T24 HPRT	T24 HPRT	**2b**
