# Supplementary material for: A reported 20-gene expression signature to predict lymph node-positive disease at radical cystectomy for muscle-invasive bladder cancer is clinically not applicable
Source: PLoS One. 2017 Mar 20;12(3):e0174039. doi: 10.1371/journal.pone.0174039 (PMC5358850; doi:10.1371/journal.pone.0174039)
Supplement: S2 Fig — (PDF) [file pone.0174039.s002.pdf]

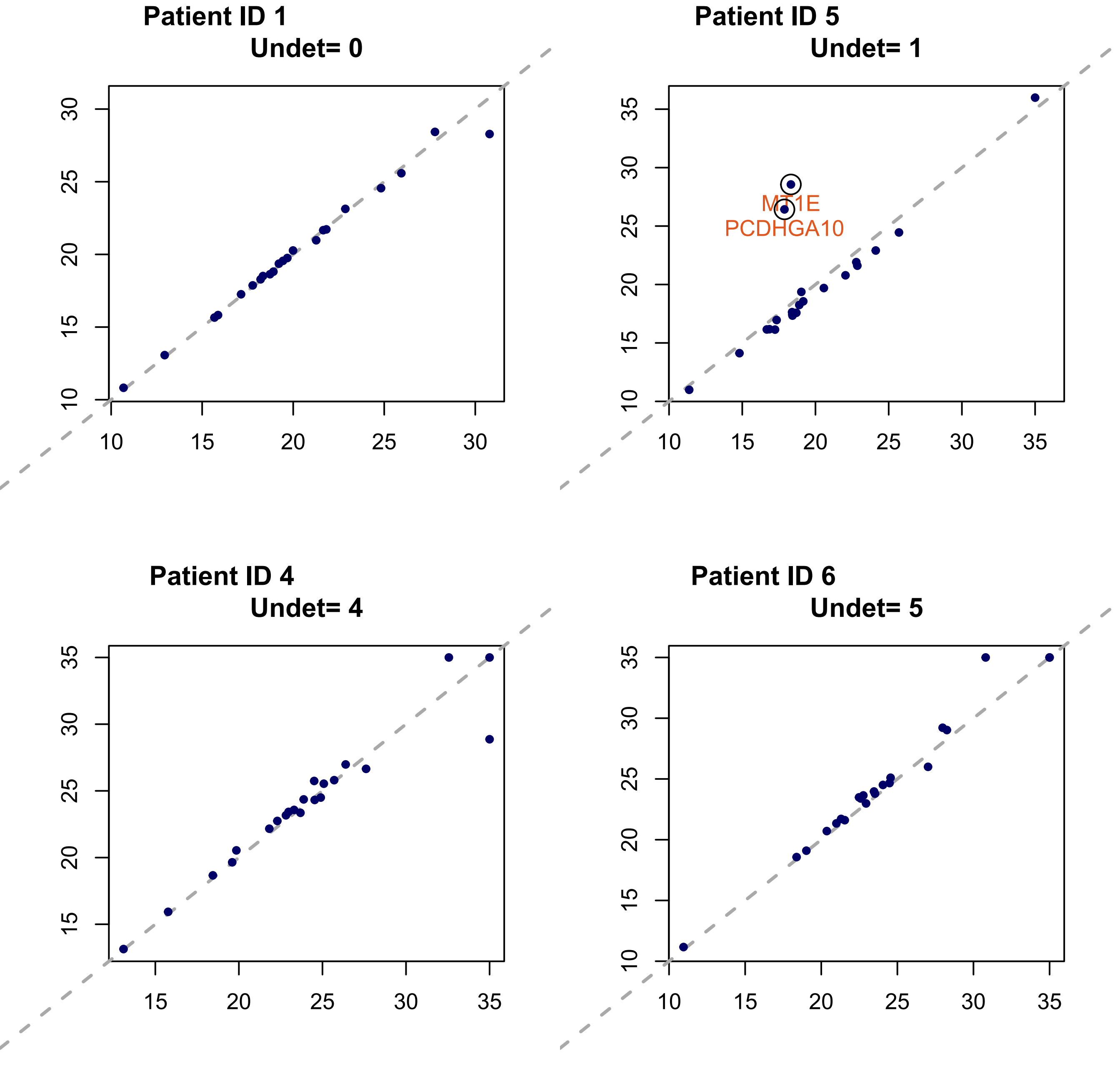
Patient ID 1
Undet= 0
Patient ID 5
Undet= 1
MT1E
PCDHGA10
Patient ID 4
Undet= 4
Patient ID 6
Undet= 5
10
15
20
25
30
35

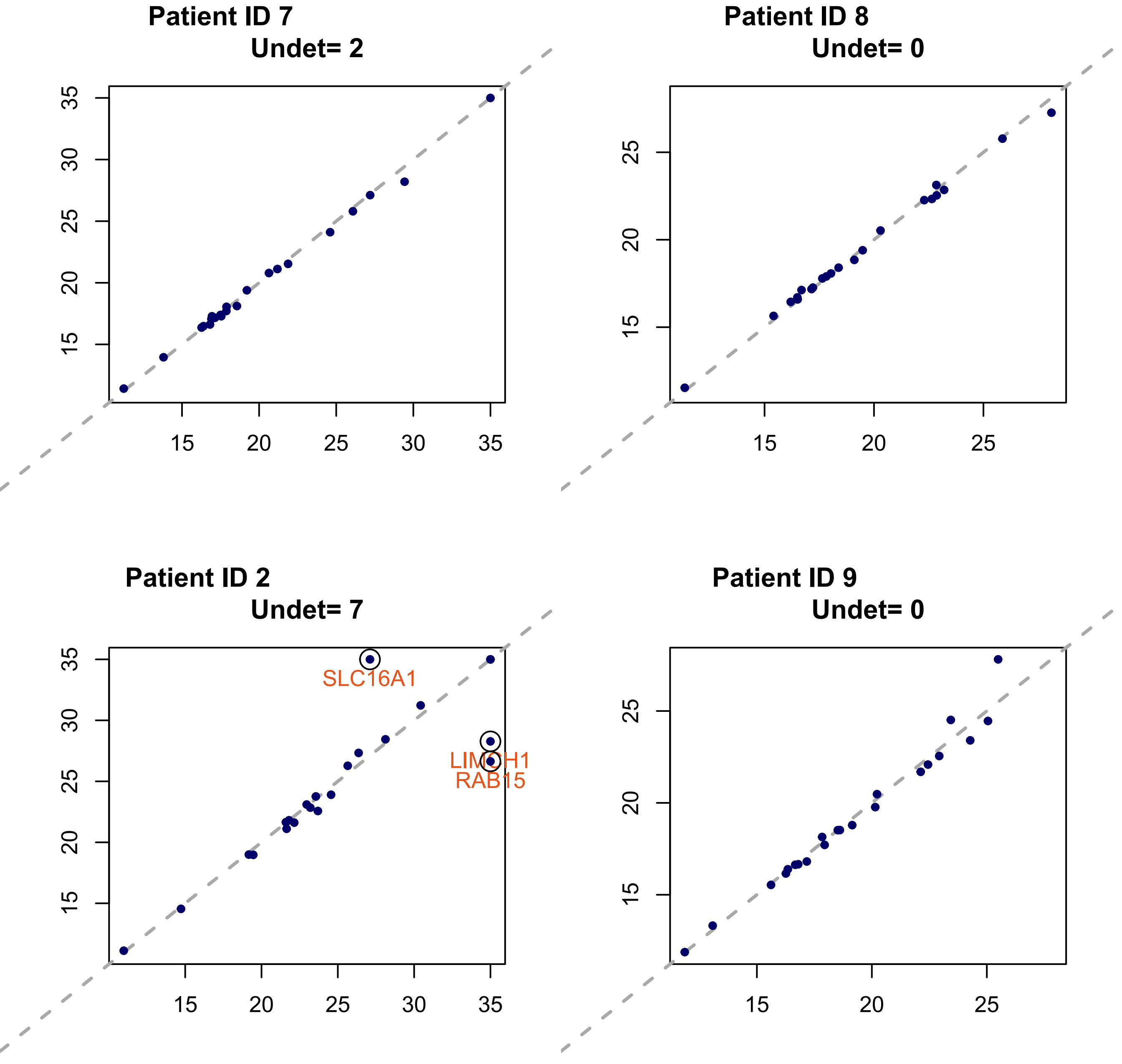
Patient ID 7
Undet= 2
Patient ID 8
Undet= 0
Patient ID 2
Undet= 7
SLC16A1
LIMCH1
RAB15
Patient ID 9
Undet= 0

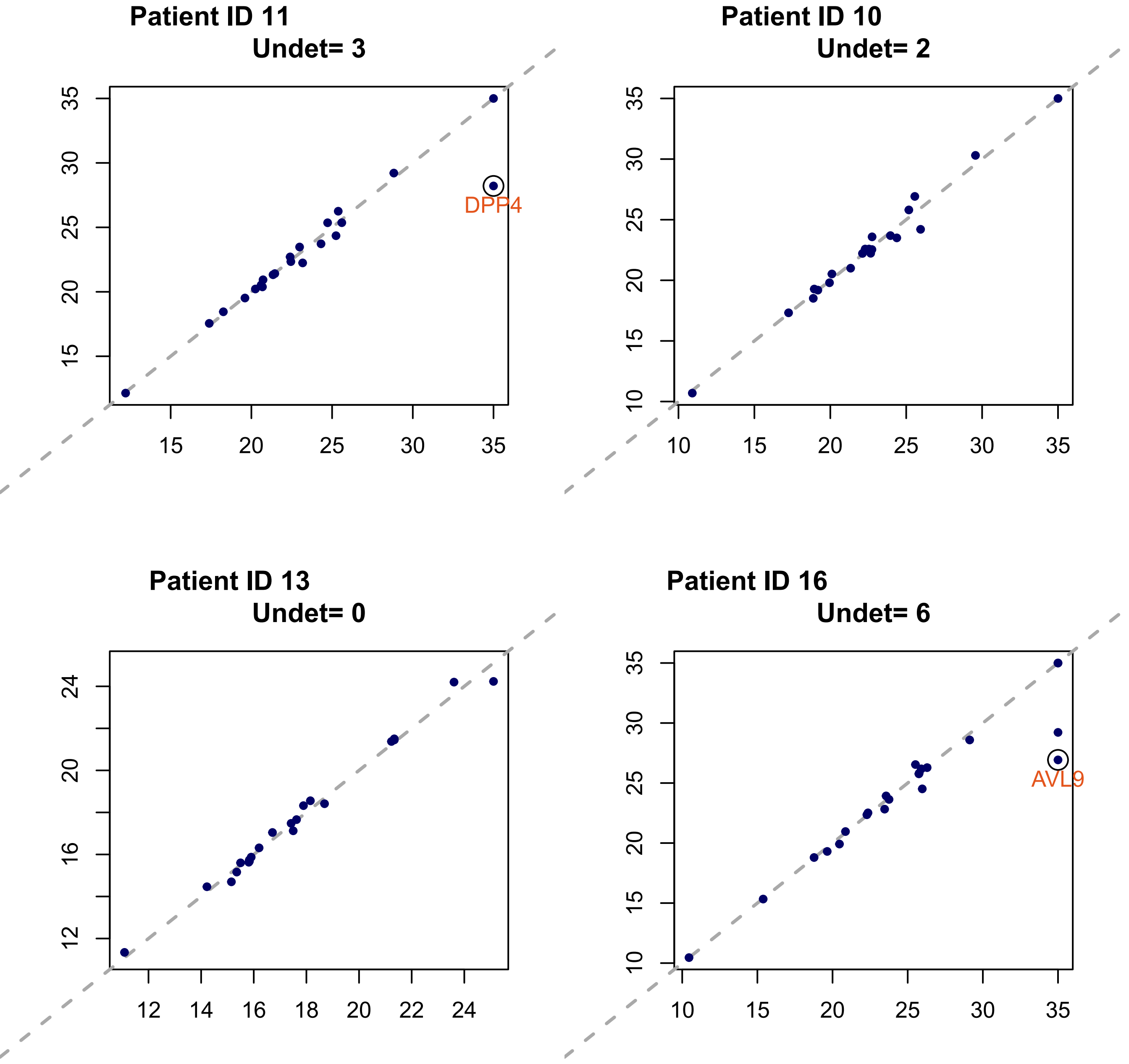
Patient ID 11
Undet= 3
DPP4
15
20
25
30
35
Patient ID 10
Undet= 2
10
Patient ID 13
Undet= 0
12
16
20
24
14
18
22
Patient ID 16
Undet= 6
AVL9

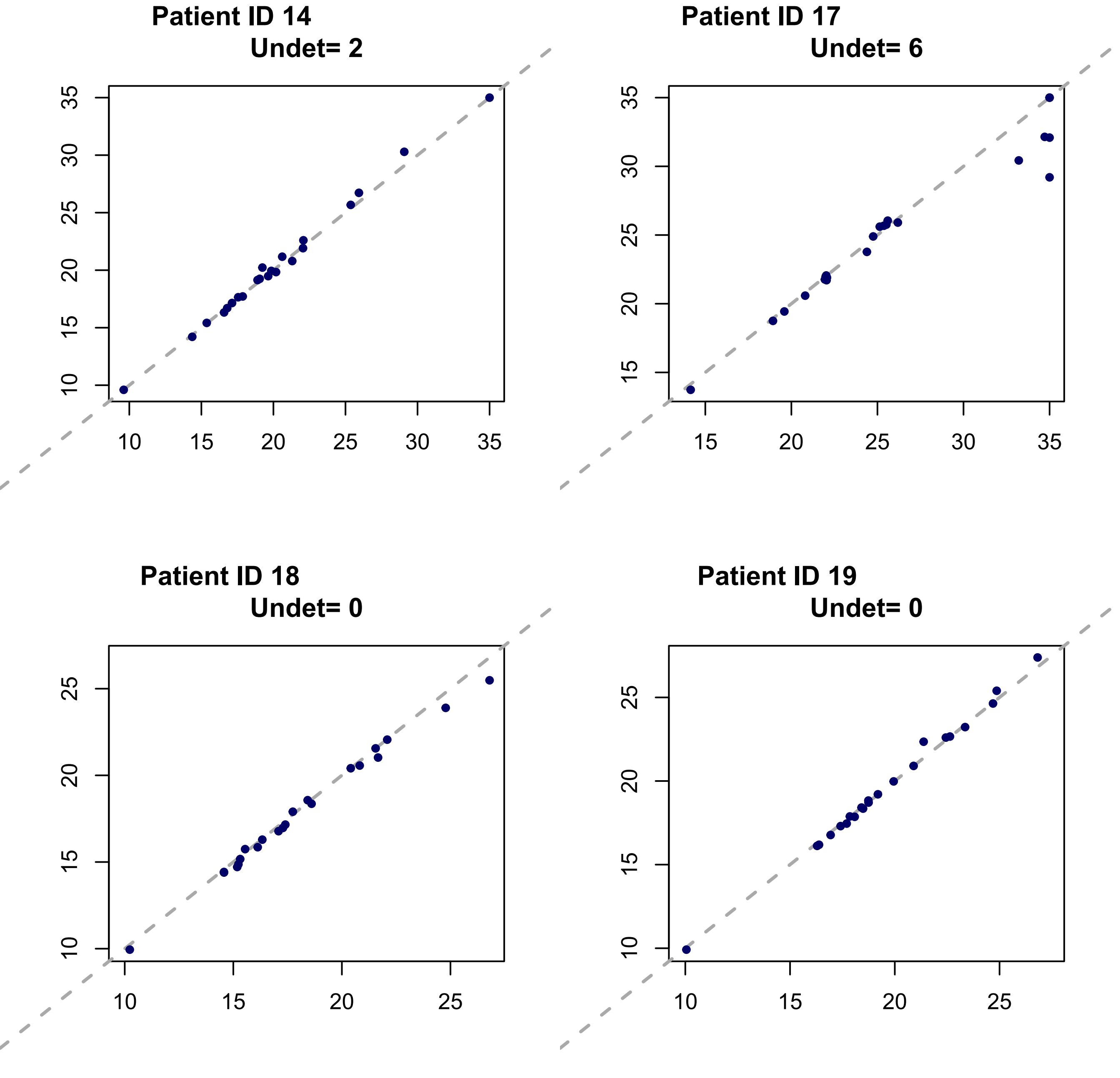

Patient ID 14
Undet= 2
10 15 20 25 30 35
10 15 20 25 30 35
Patient ID 17
Undet= 6
15 20 25 30 35
15 20 25 30 35
Patient ID 18
Undet= 0
10 15 20 25
10 15 20 25
Patient ID 19
Undet= 0
10 15 20 25
10 15 20 25

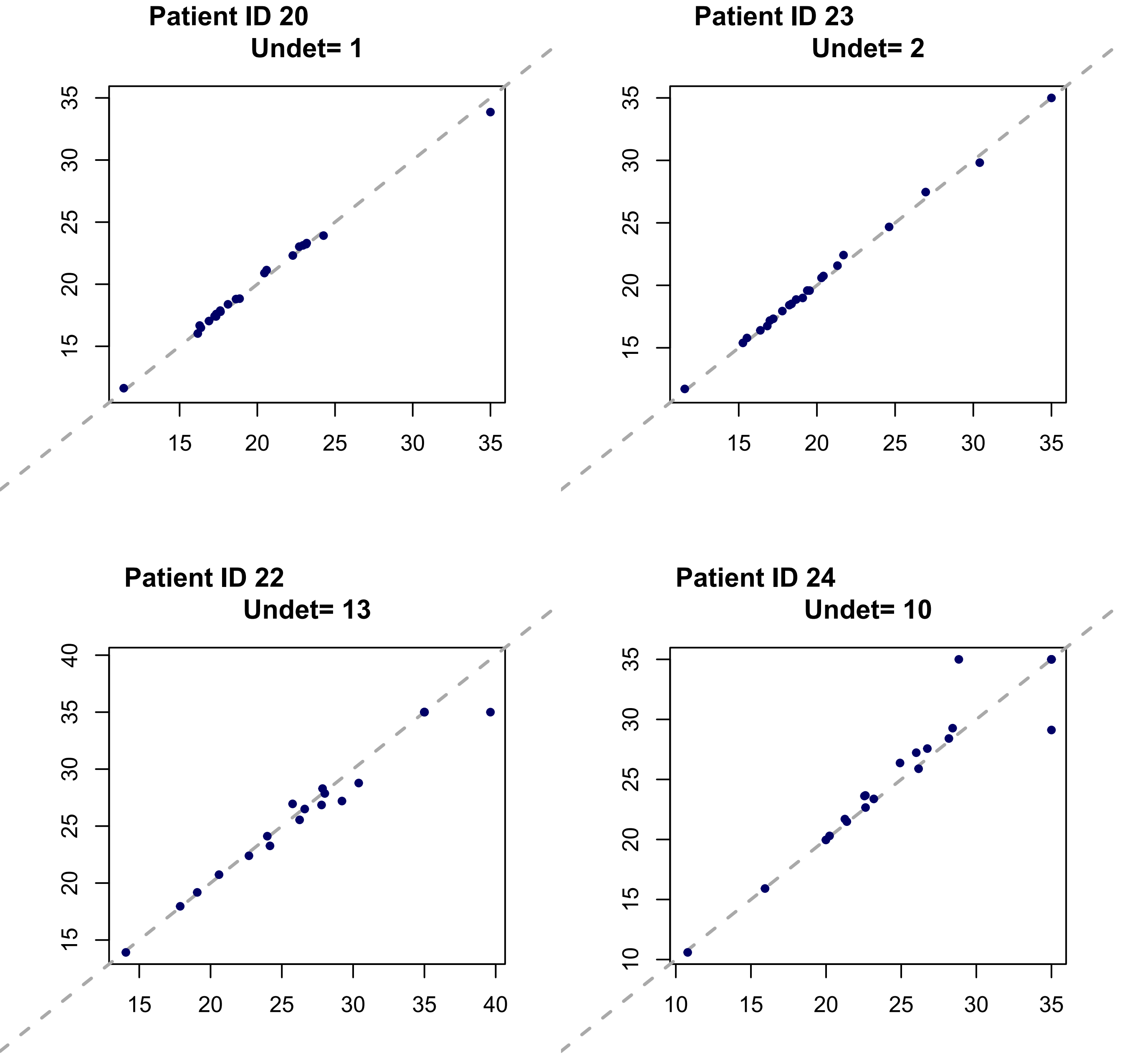
Patient ID 20
Undet= 1
Patient ID 23
Undet= 2
Patient ID 22
Undet= 13
Patient ID 24
Undet= 10

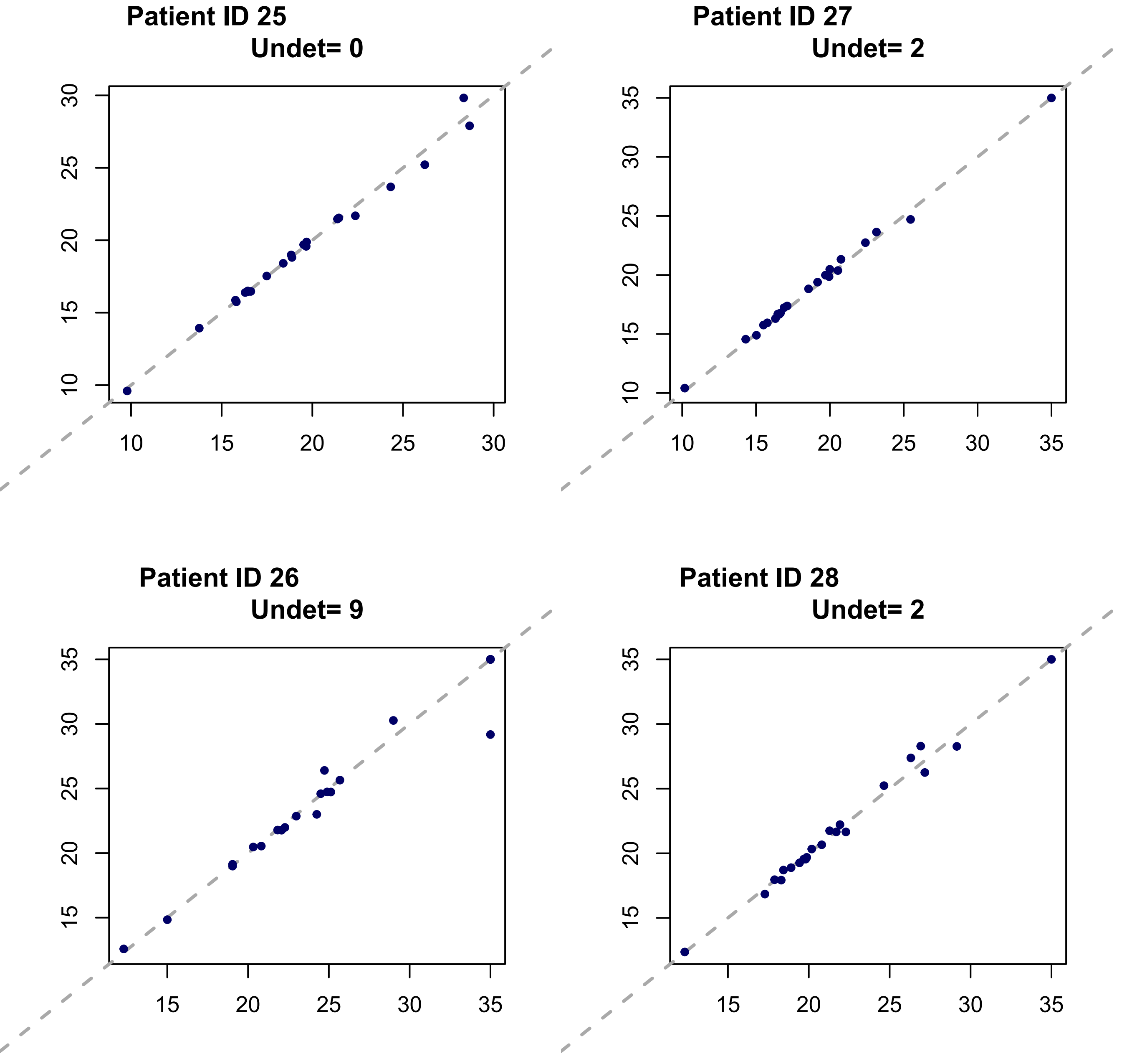
Patient ID 25
Undet= 0
Patient ID 27
Undet= 2
Patient ID 26
Undet= 9
Patient ID 28
Undet= 2

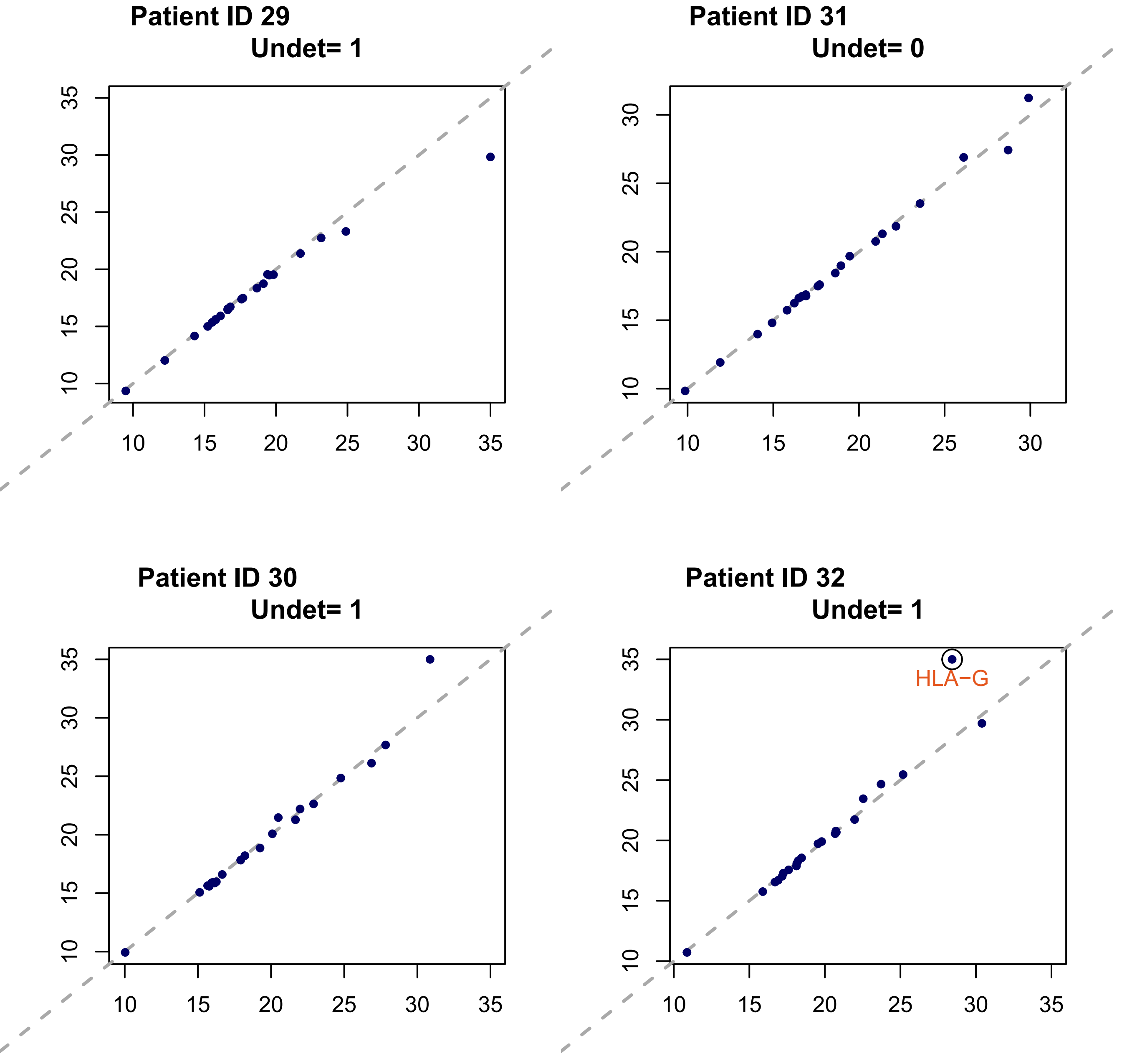
Patient ID 29
Undet= 1
Patient ID 31
Undet= 0
Patient ID 30
Undet= 1
Patient ID 32
Undet= 1
HLA−G

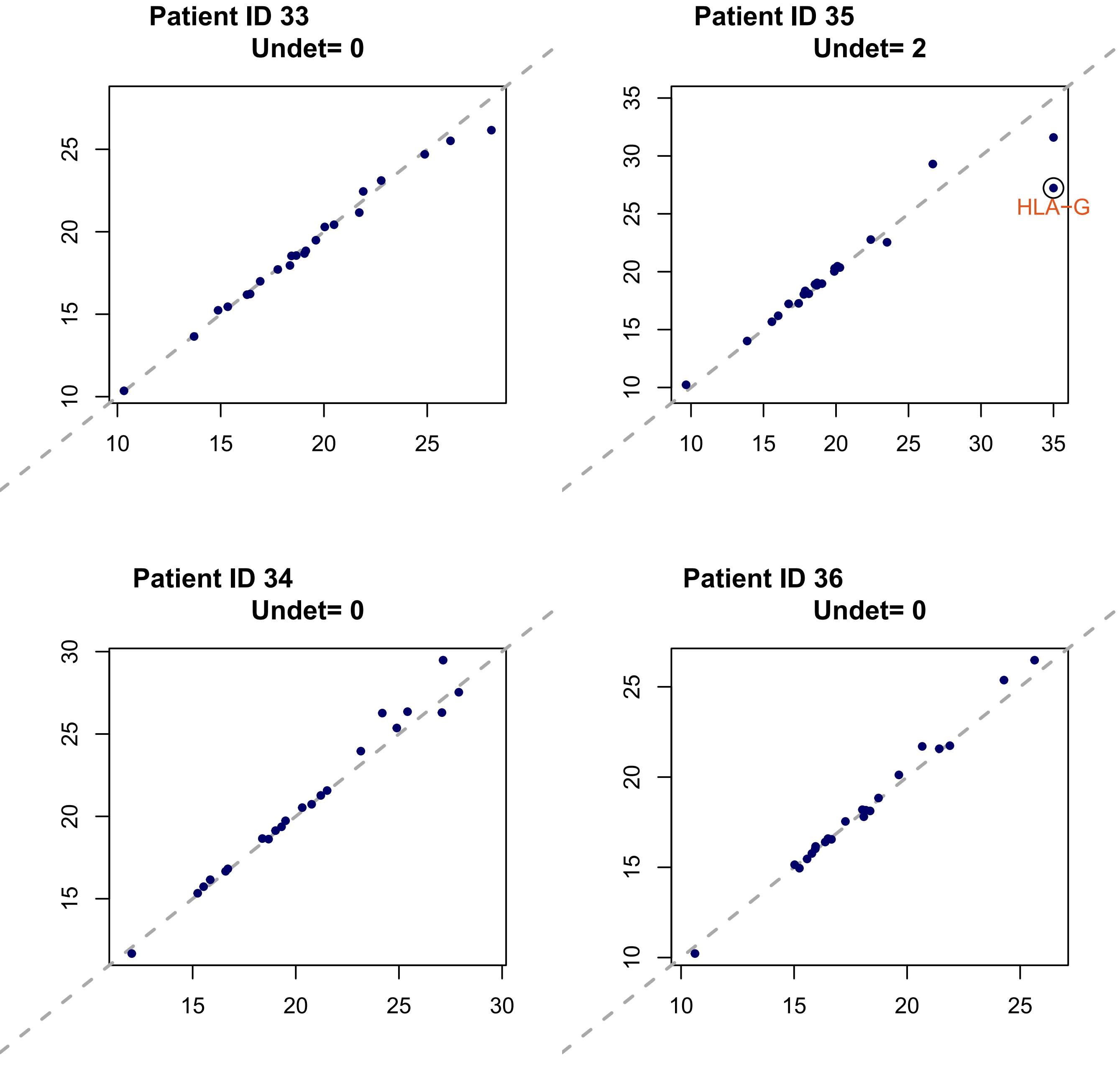
Patient ID 33
Undet= 0
10
15
20
25
10
15
20
25
Patient ID 35
Undet= 2
10
15
20
25
30
35
10
15
20
25
30
35
HLA-G
Patient ID 34
Undet= 0
15
20
25
30
15
20
25
30
Patient ID 36
Undet= 0
10
15
20
25
10
15
20
25

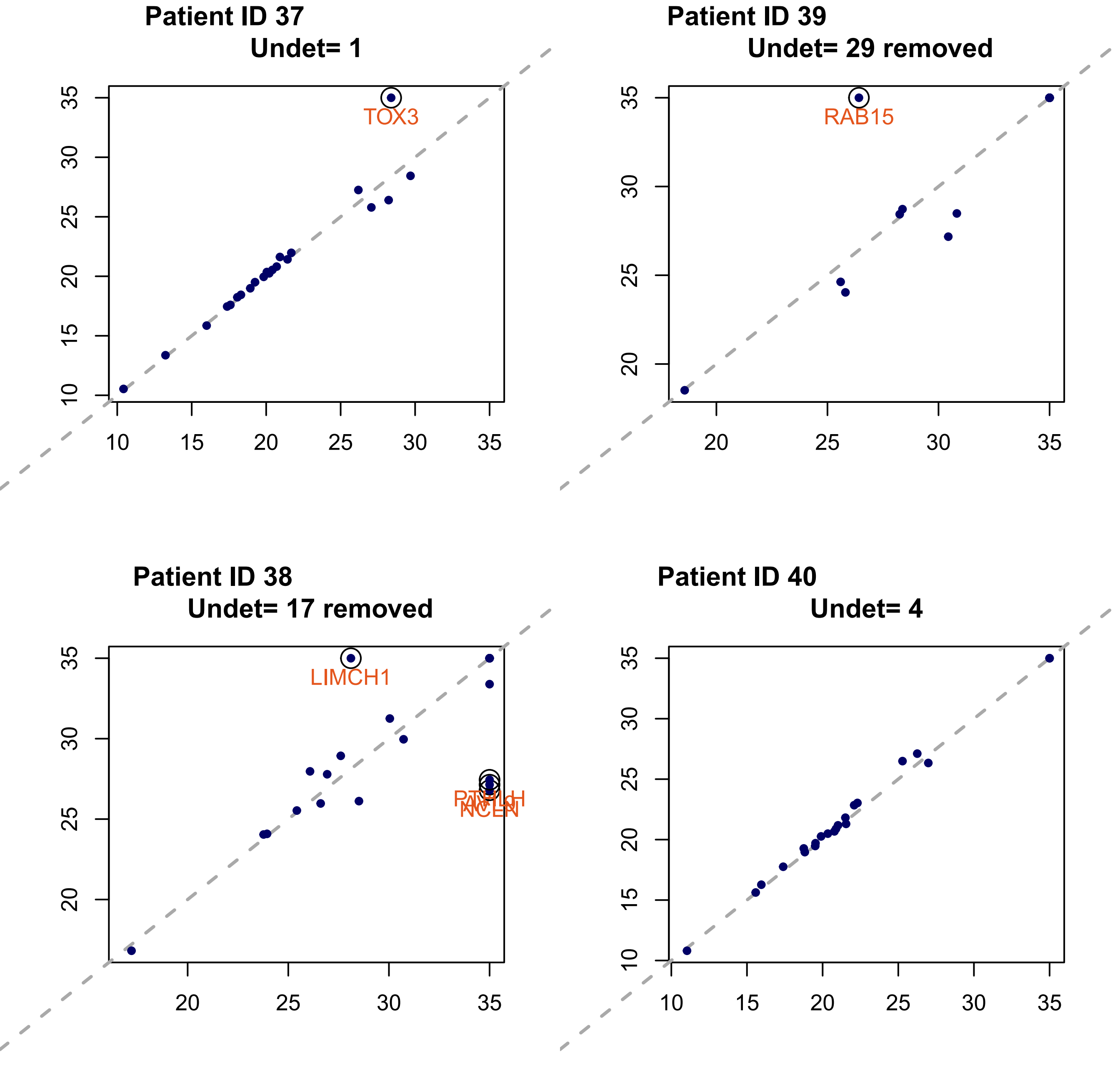
Patient ID 37
Undet= 1
TOX3
Patient ID 39
Undet= 29 removed
RAB15
Patient ID 38
Undet= 17 removed
LIMCH1
Patient ID 40
Undet= 4
10
15
20
25
30
35

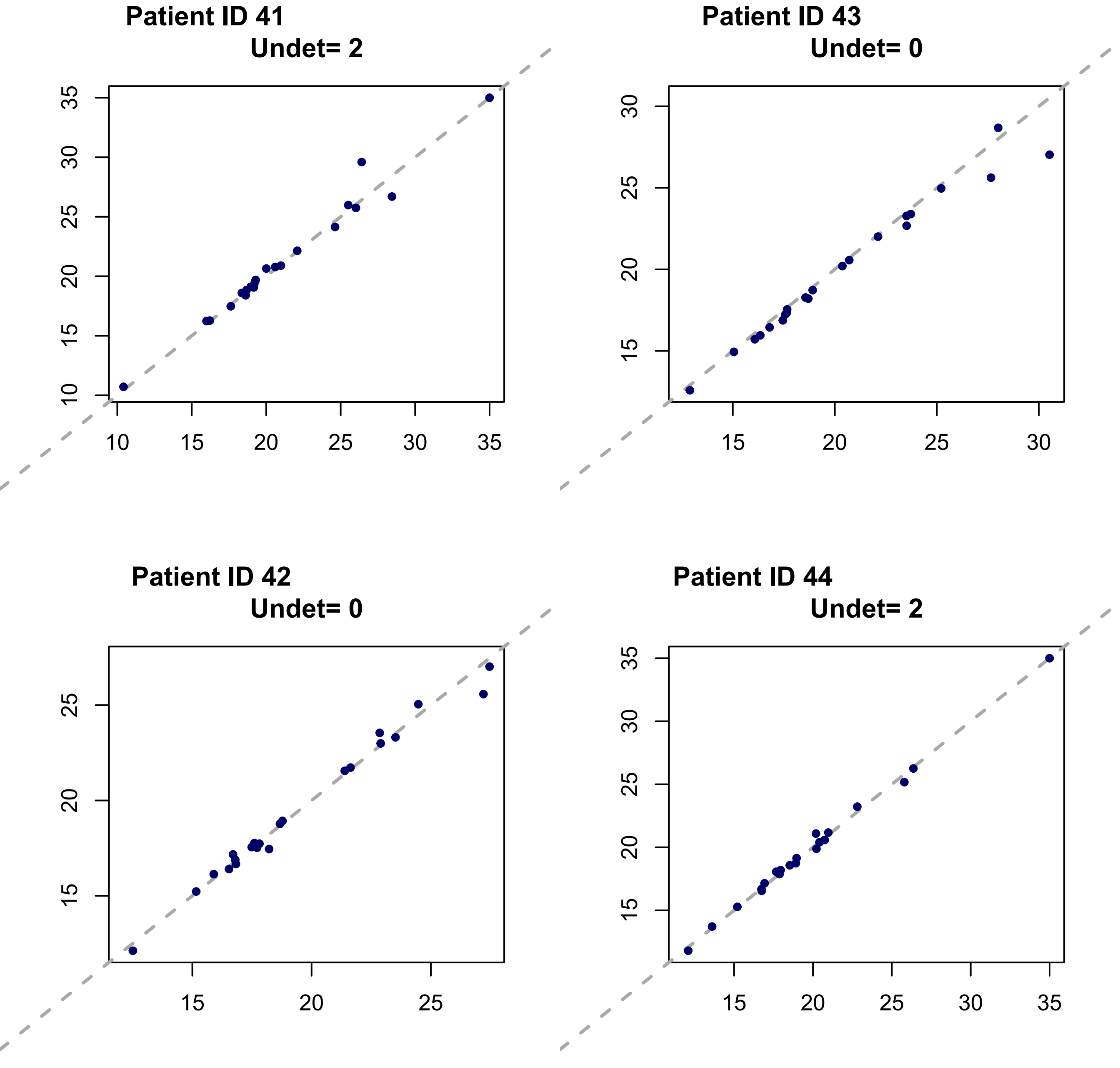
Patient ID 41
Undet= 2
Patient ID 43
Undet= 0
Patient ID 42
Undet= 0
Patient ID 44
Undet= 2
10
15
20
25
30
35

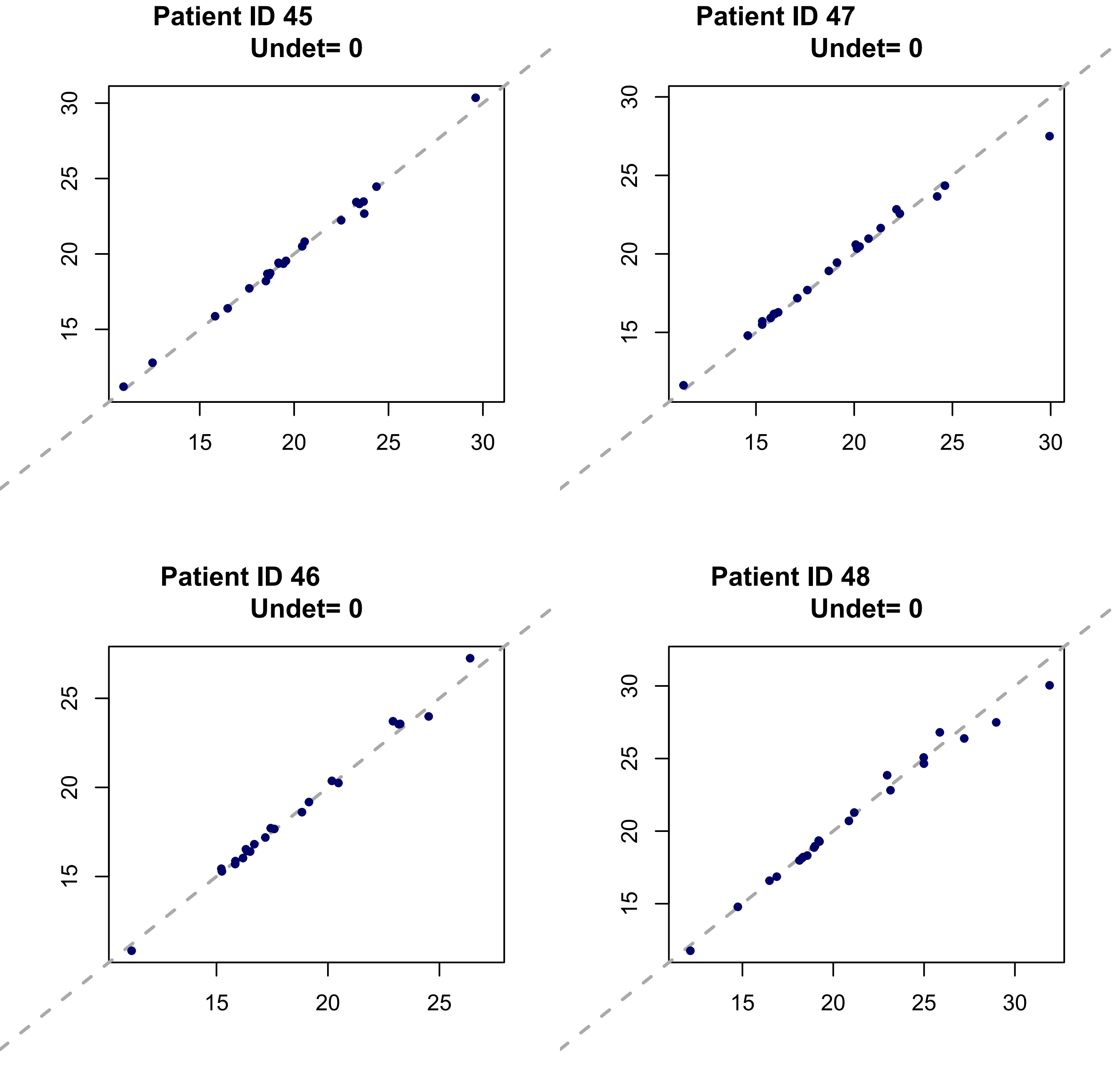

Patient ID 45
Undet= 0
15
20
25
30
Patient ID 47
Undet= 0
15
20
25
30
Patient ID 46
Undet= 0
15
20
25
Patient ID 48
Undet= 0
15
20
25
30

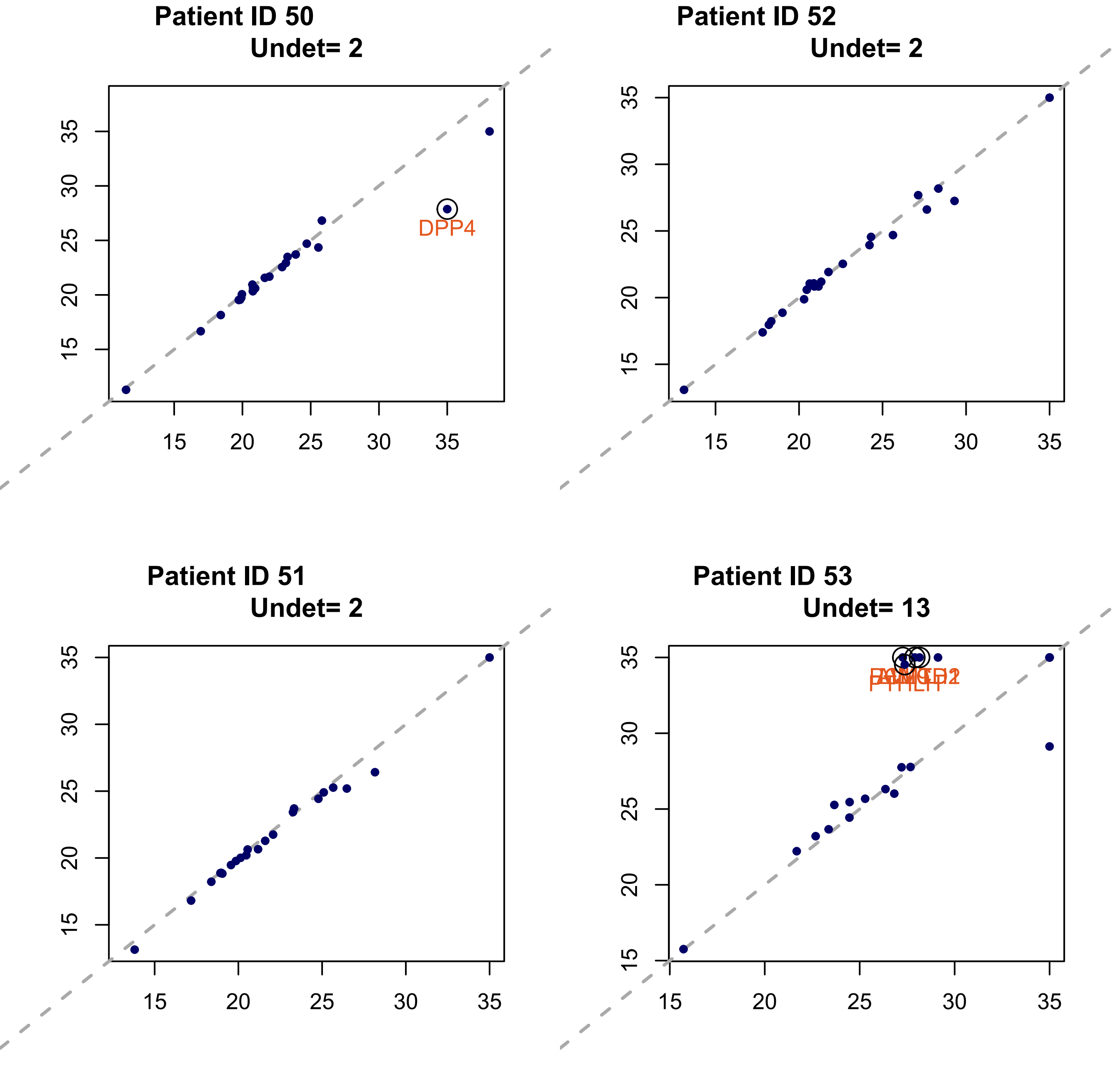
Patient ID 50
Undet= 2
DPP4
15 20 25 30 35
Patient ID 52
Undet= 2
15 20 25 30 35
Patient ID 51
Undet= 2
15 20 25 30 35
Patient ID 53
Undet= 13
15 20 25 30 35

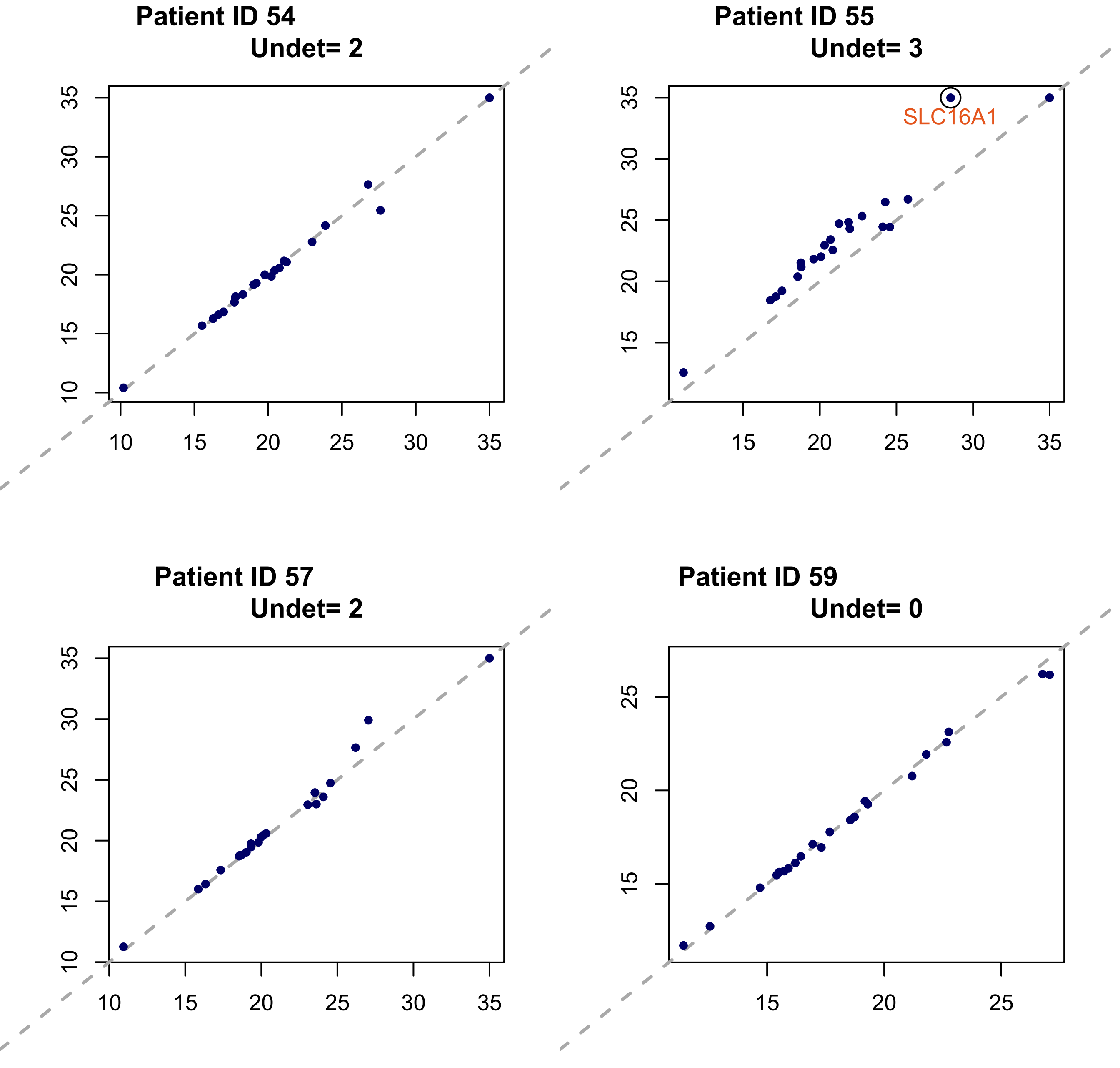

Patient ID 54
Undet= 2
Patient ID 55
Undet= 3
SLC16A1
Patient ID 57
Undet= 2
Patient ID 59
Undet= 0

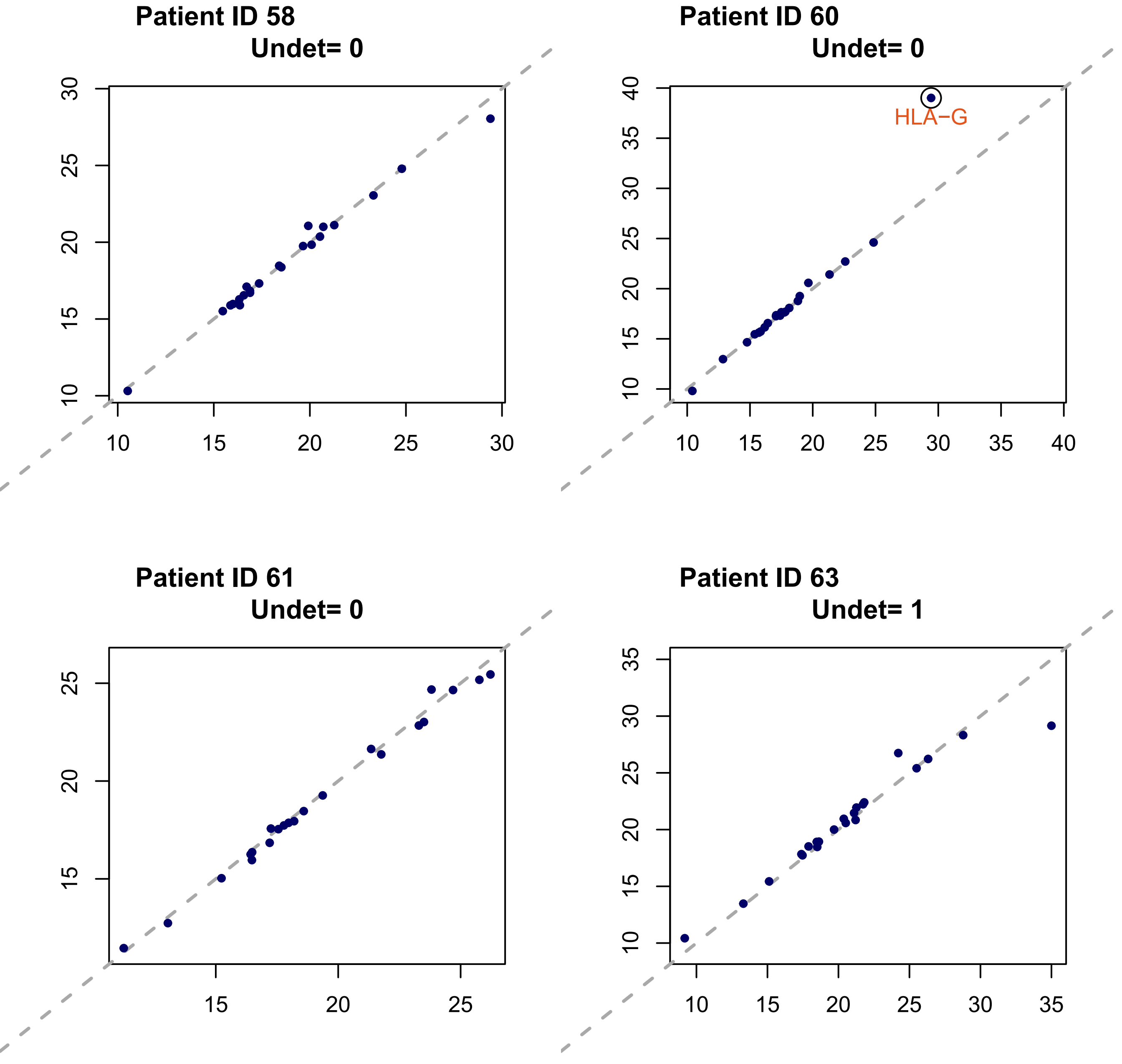
Patient ID 58
Undet= 0
Patient ID 60
Undet= 0
HLA−G
Patient ID 61
Undet= 0
Patient ID 63
Undet= 1

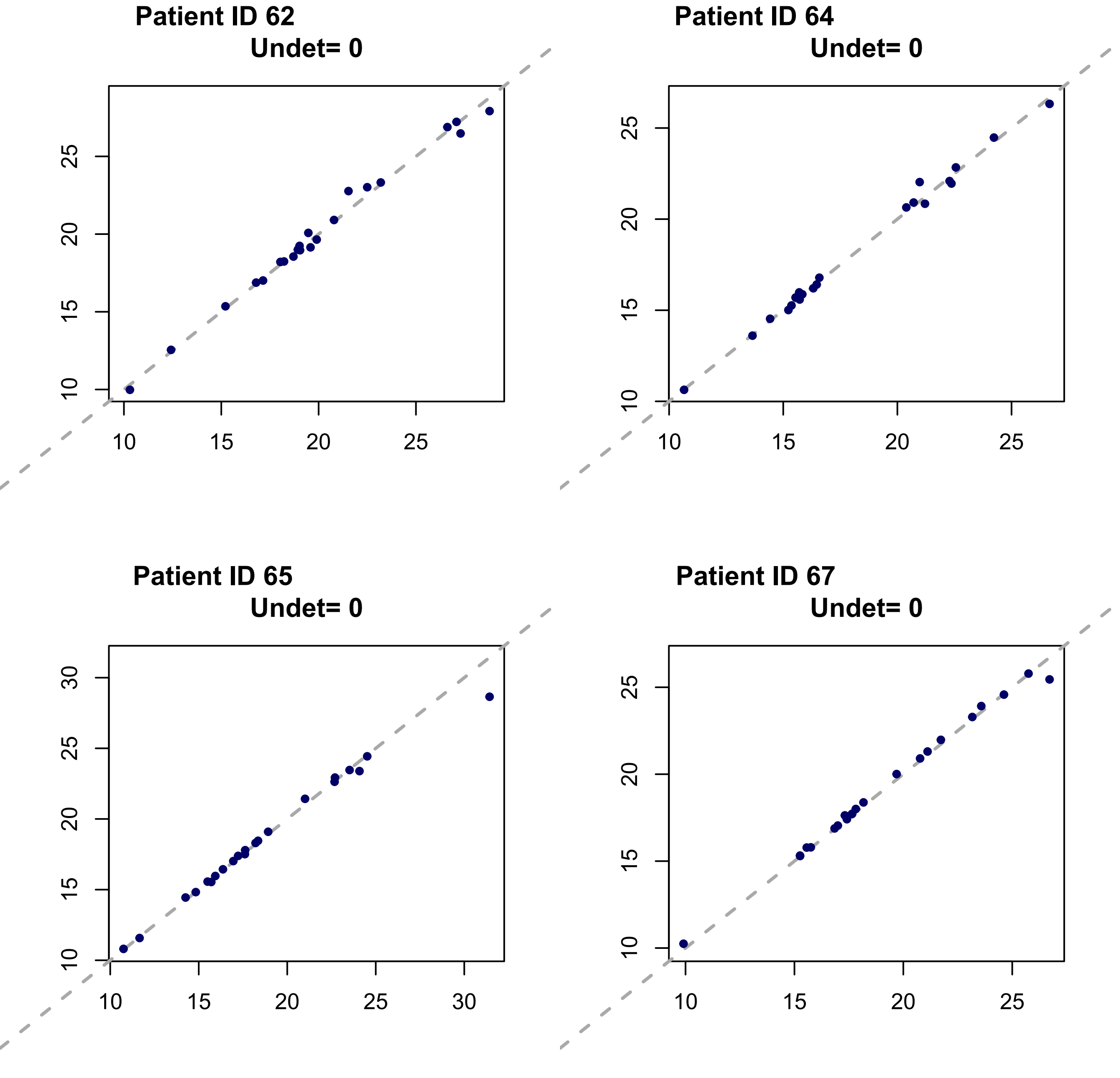

Patient ID 62
Undet= 0
10 15 20 25
10 15 20 25
Patient ID 64
Undet= 0
10 15 20 25
10 15 20 25
Patient ID 65
Undet= 0
10 15 20 25 30
10 15 20 25 30
Patient ID 67
Undet= 0
10 15 20 25
10 15 20 25

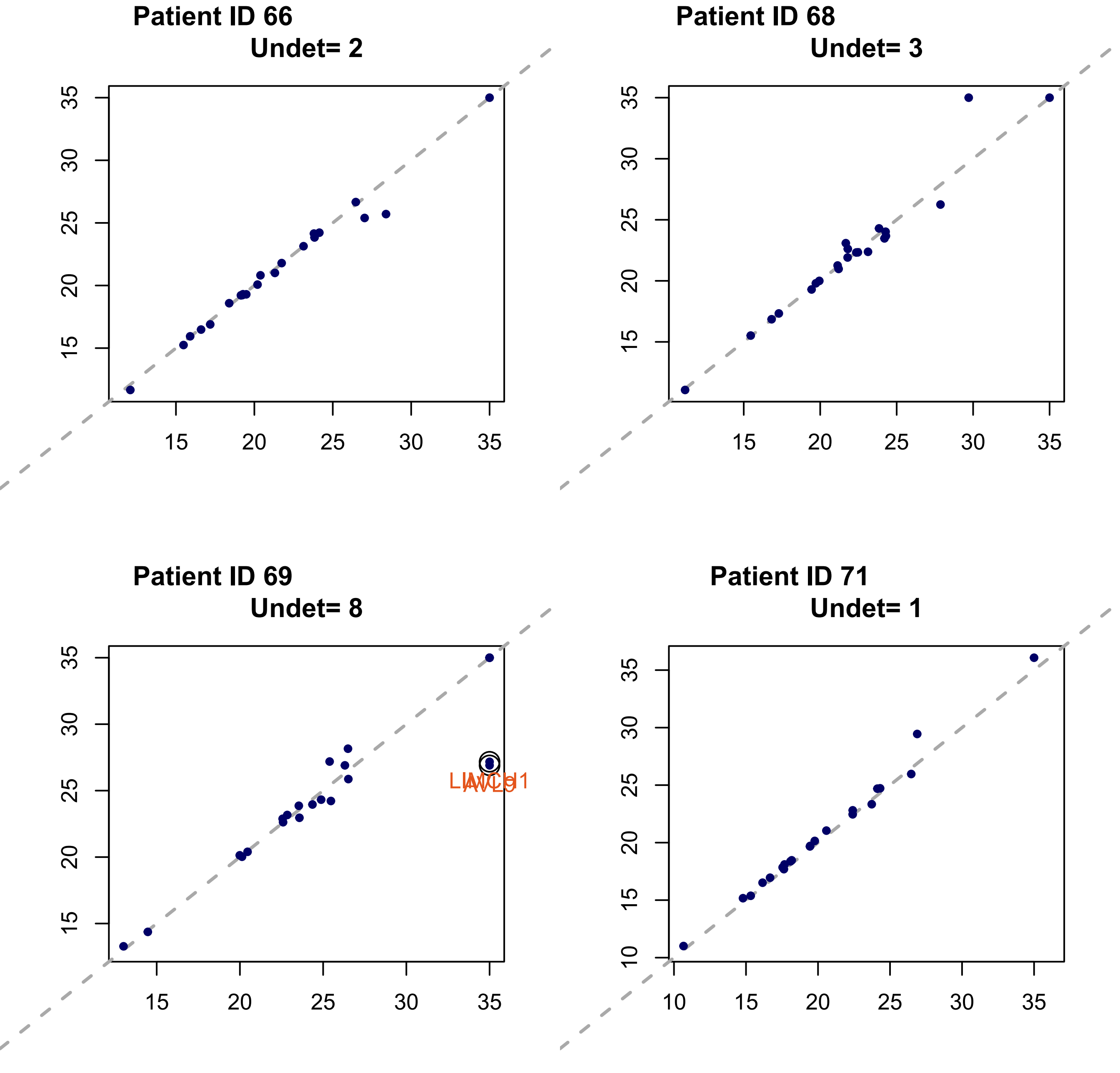
Patient ID 66
Undet= 2
15 20 25 30 35
15 20 25 30 35
Patient ID 68
Undet= 3
15 20 25 30 35
15 20 25 30 35
Patient ID 69
Undet= 8
15 20 25 30 35
15 20 25 30 35
Patient ID 71
Undet= 1
10 15 20 25 30 35
10 15 20 25 30 35

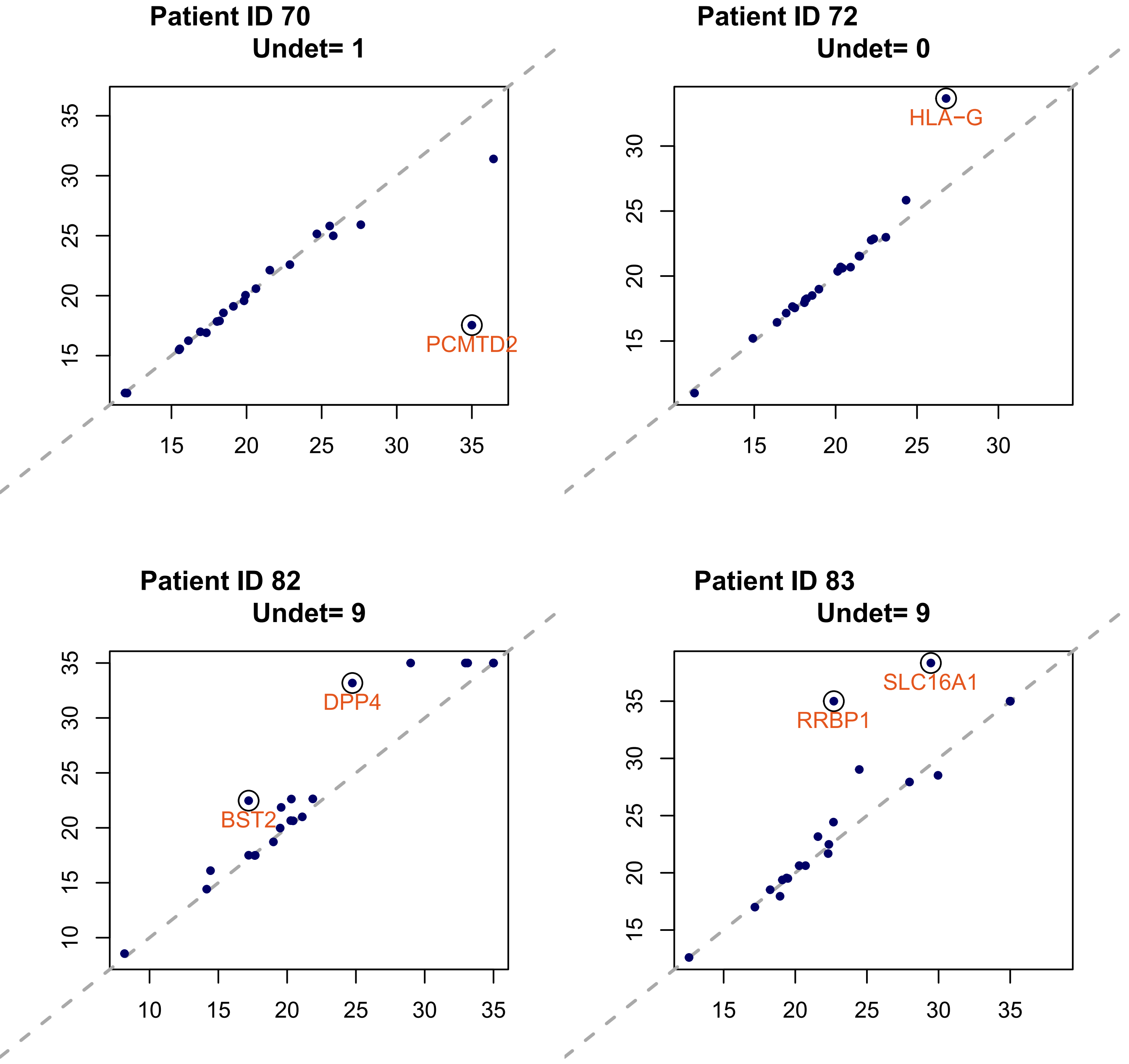
Patient ID 70
Undet= 1
PCMTD2
Patient ID 72
Undet= 0
HLA−G
Patient ID 82
Undet= 9
DPP4
BST2
Patient ID 83
Undet= 9
SLC16A1
RRBP1

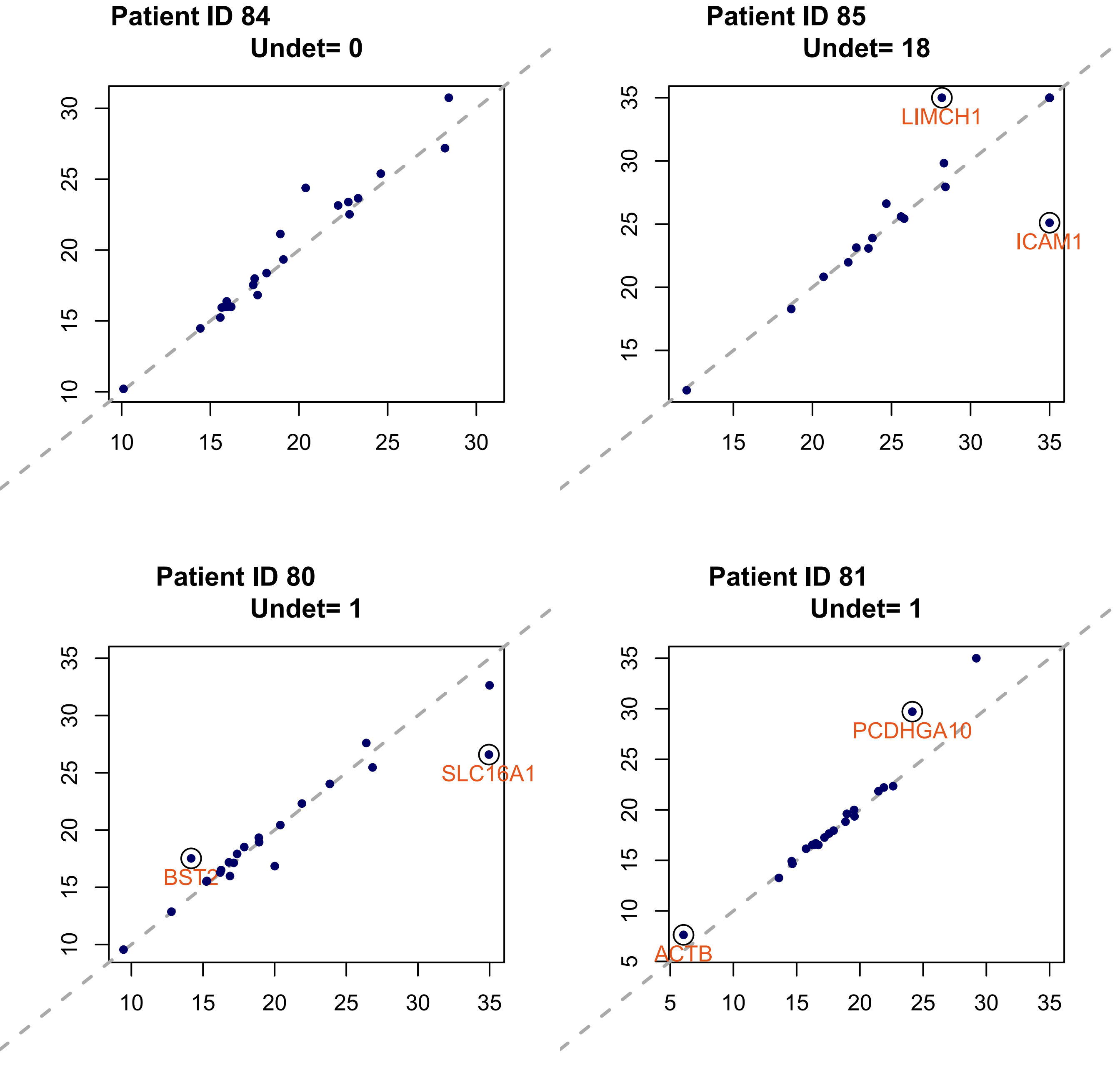

Patient ID 84
Undet= 0
Patient ID 85
Undet= 18
LIMCH1
ICAM1
Patient ID 80
Undet= 1
SLC16A1
BST2
Patient ID 81
Undet= 1
PCDHGA10
ACTB

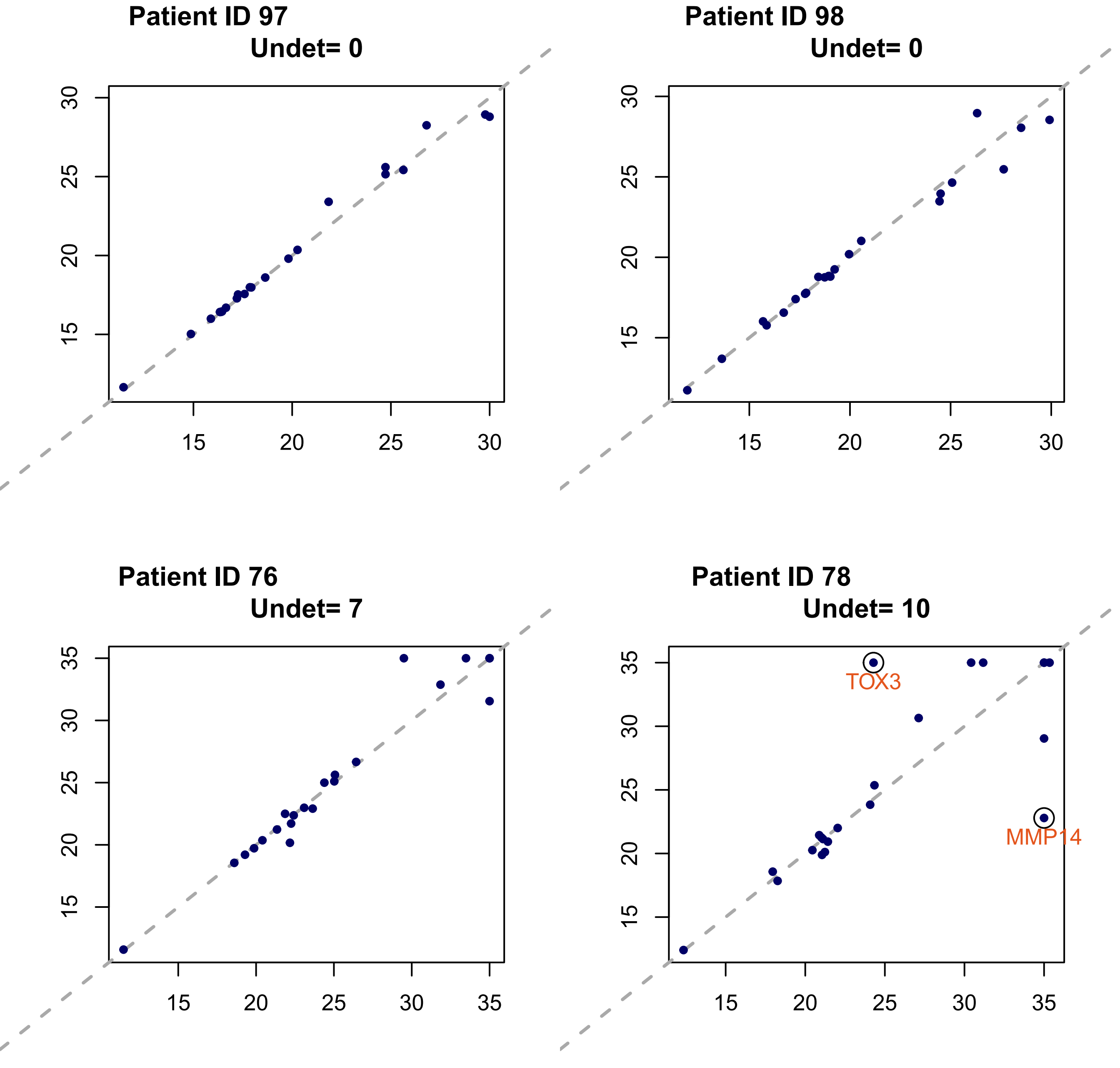

Patient ID 97
Undet= 0
Patient ID 98
Undet= 0
Patient ID 76
Undet= 7
Patient ID 78
Undet= 10
TOX3
MMP14
15
20
25
30
35

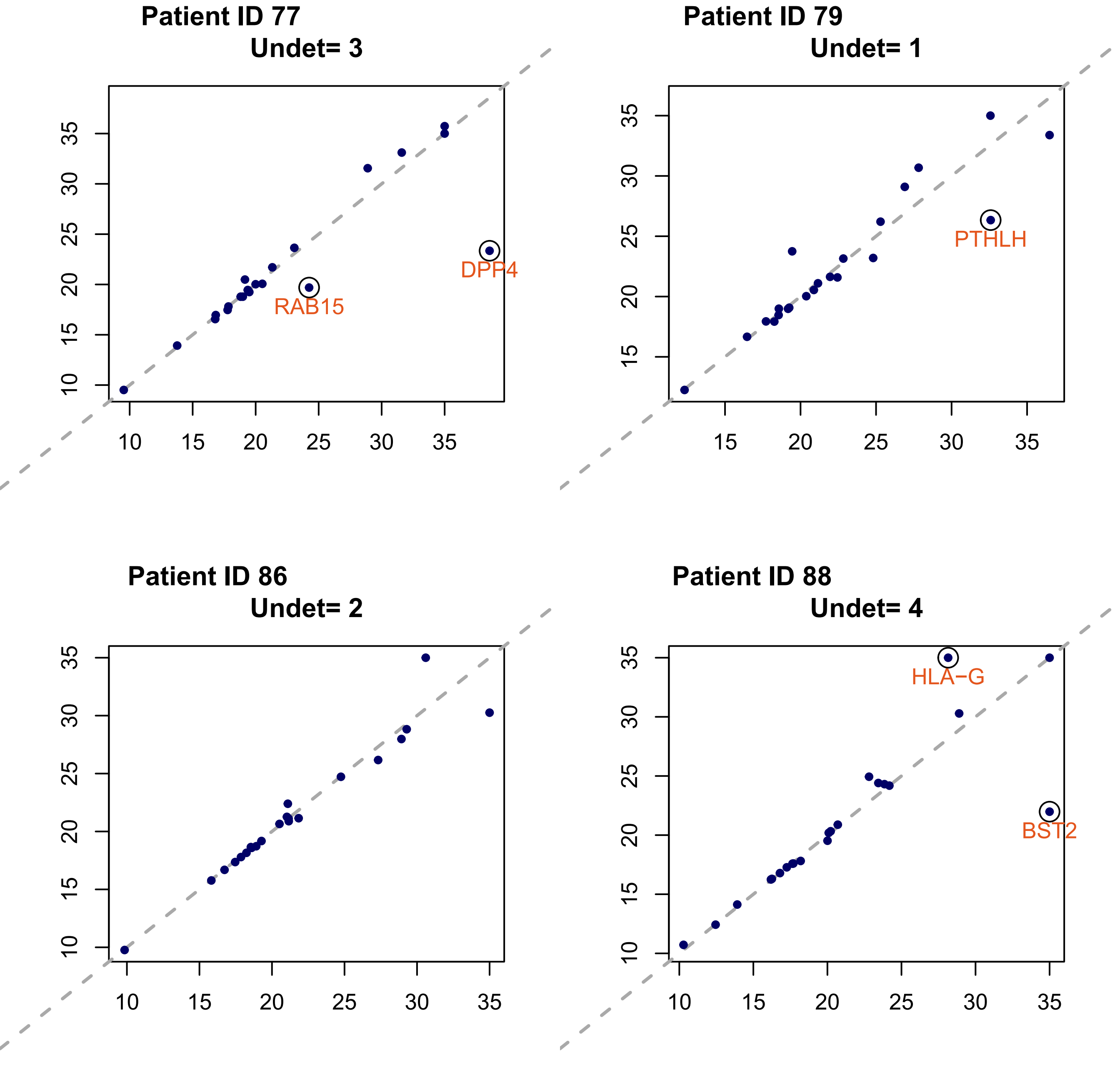
Patient ID 77
Undet= 3
RAB15
DPP4
Patient ID 79
Undet= 1
PTHLH
Patient ID 86
Undet= 2
Patient ID 88
Undet= 4
HLA−G
BST2

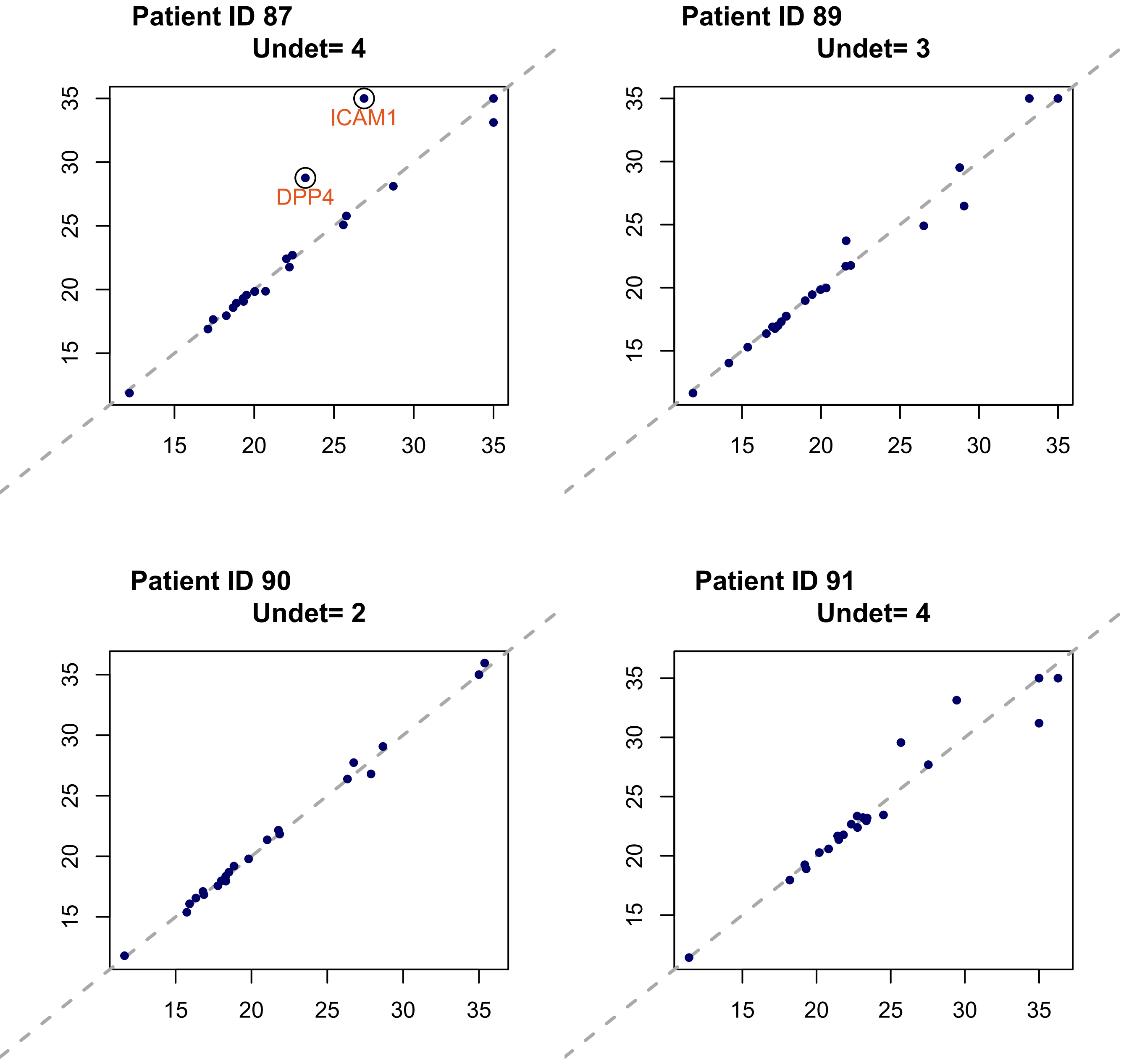
Patient ID 87
Undet= 4
ICAM1
DPP4
Patient ID 89
Undet= 3
Patient ID 90
Undet= 2
Patient ID 91
Undet= 4
15
20
25
30
35

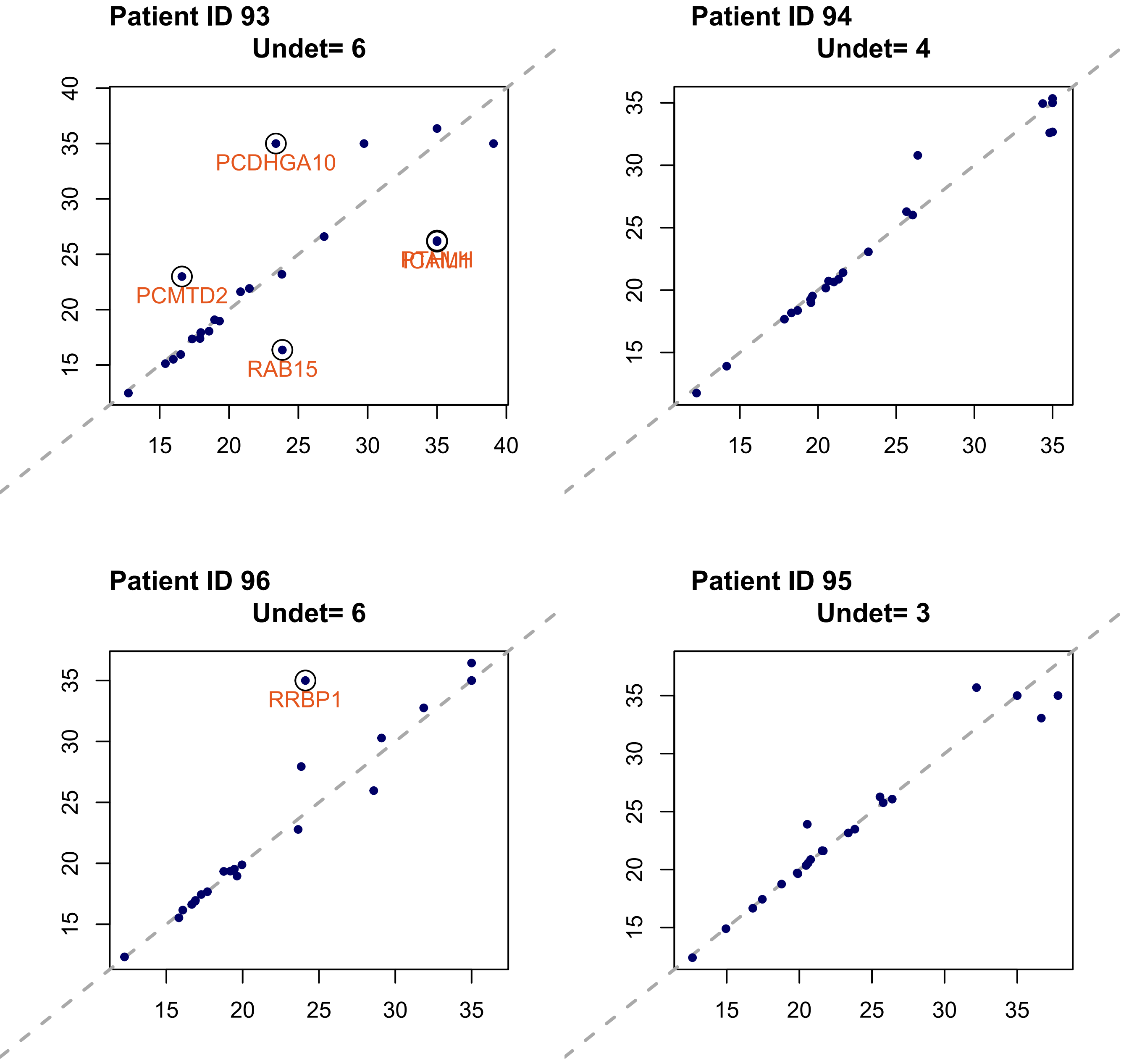
Patient ID 93
Undet= 6
PCDHGA10
PCMTD2
RAB15
Patient ID 94
Undet= 4
Patient ID 96
Undet= 6
RRBP1
Patient ID 95
Undet= 3

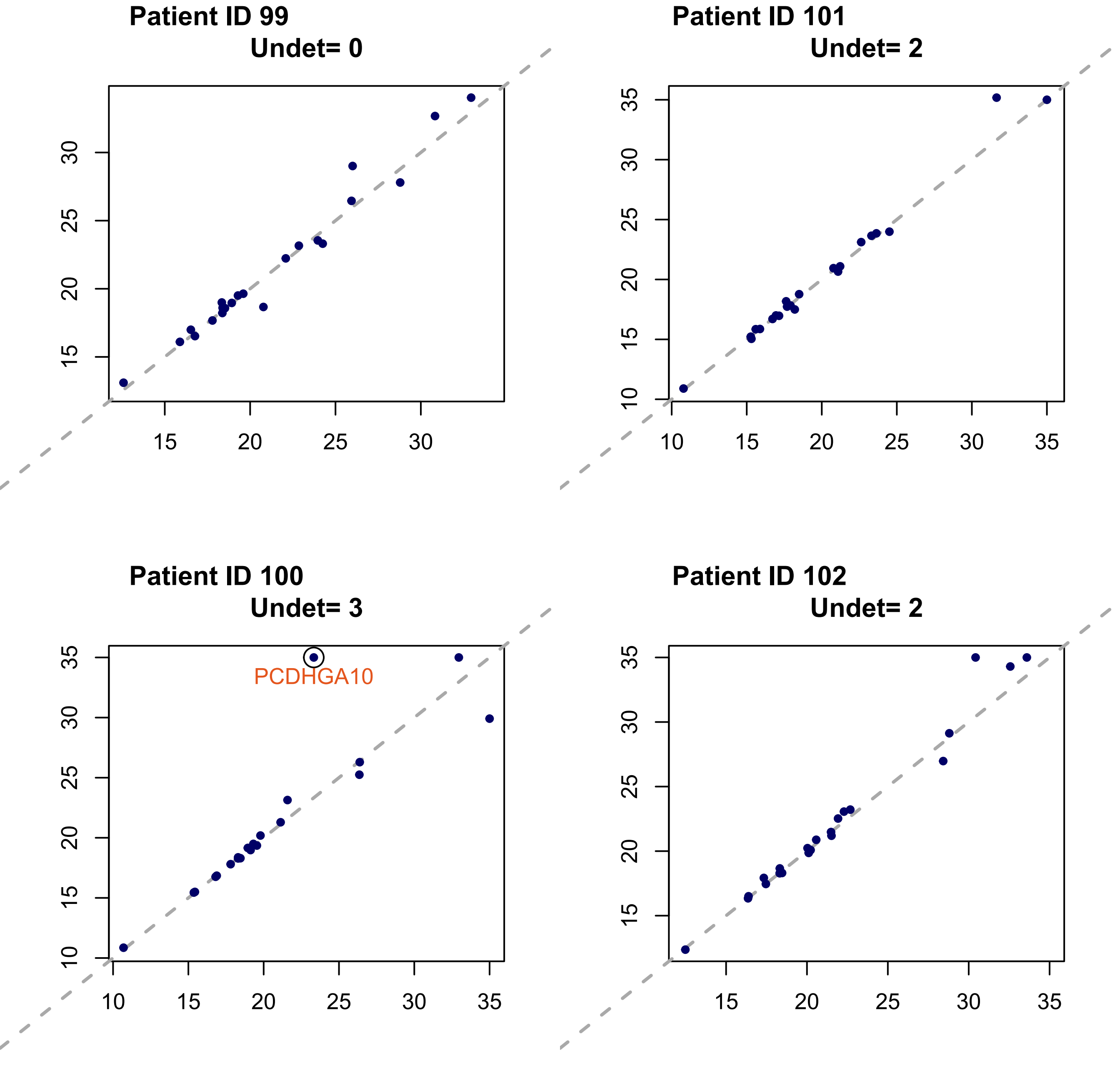
Patient ID 99
Undet= 0
15
20
25
30
Patient ID 101
Undet= 2
10
15
20
25
30
35
Patient ID 100
Undet= 3
PCDHGA10
10
15
20
25
30
35
Patient ID 102
Undet= 2
15
20
25
30
35

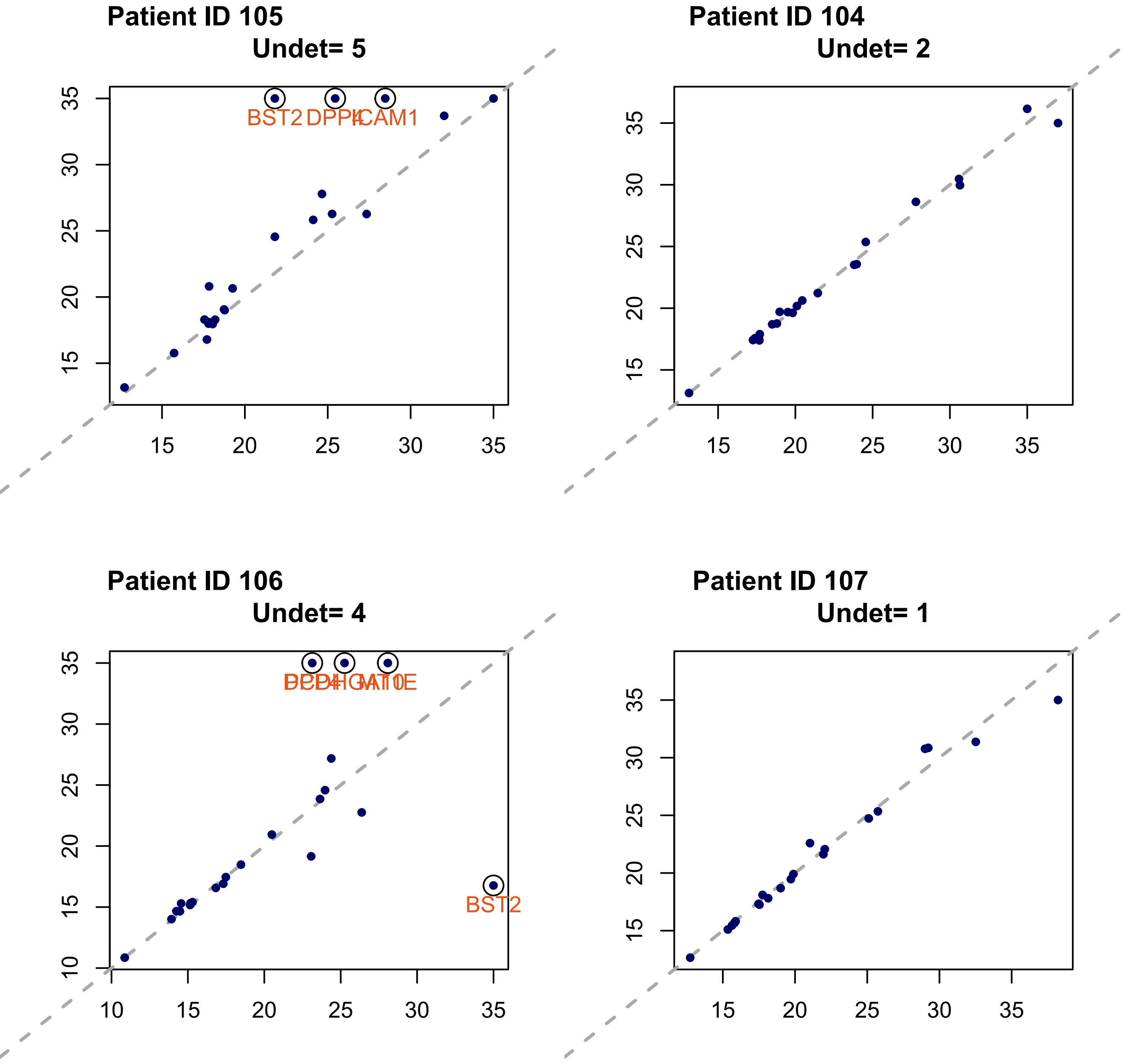
Patient ID 105
Undet= 5
BST2
DPP4
ICAM1
15
20
25
30
35
Patient ID 104
Undet= 2
15
20
25
30
35
Patient ID 106
Undet= 4
BST2
10
15
20
25
30
35
Patient ID 107
Undet= 1
15
20
25
30
35

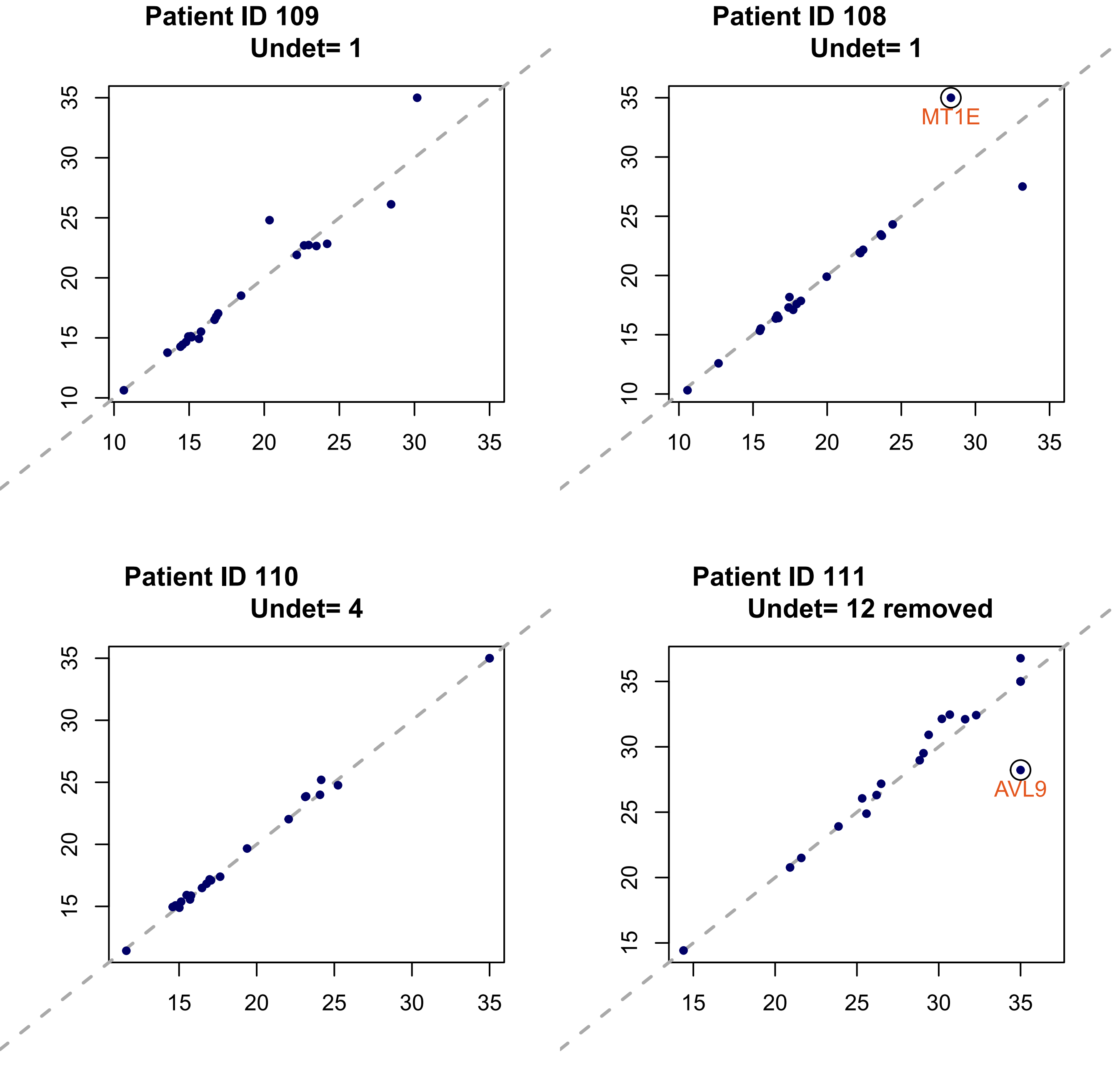
Patient ID 109
Undet= 1
Patient ID 108
Undet= 1
MT1E
Patient ID 110
Undet= 4
Patient ID 111
Undet= 12 removed
AVL9

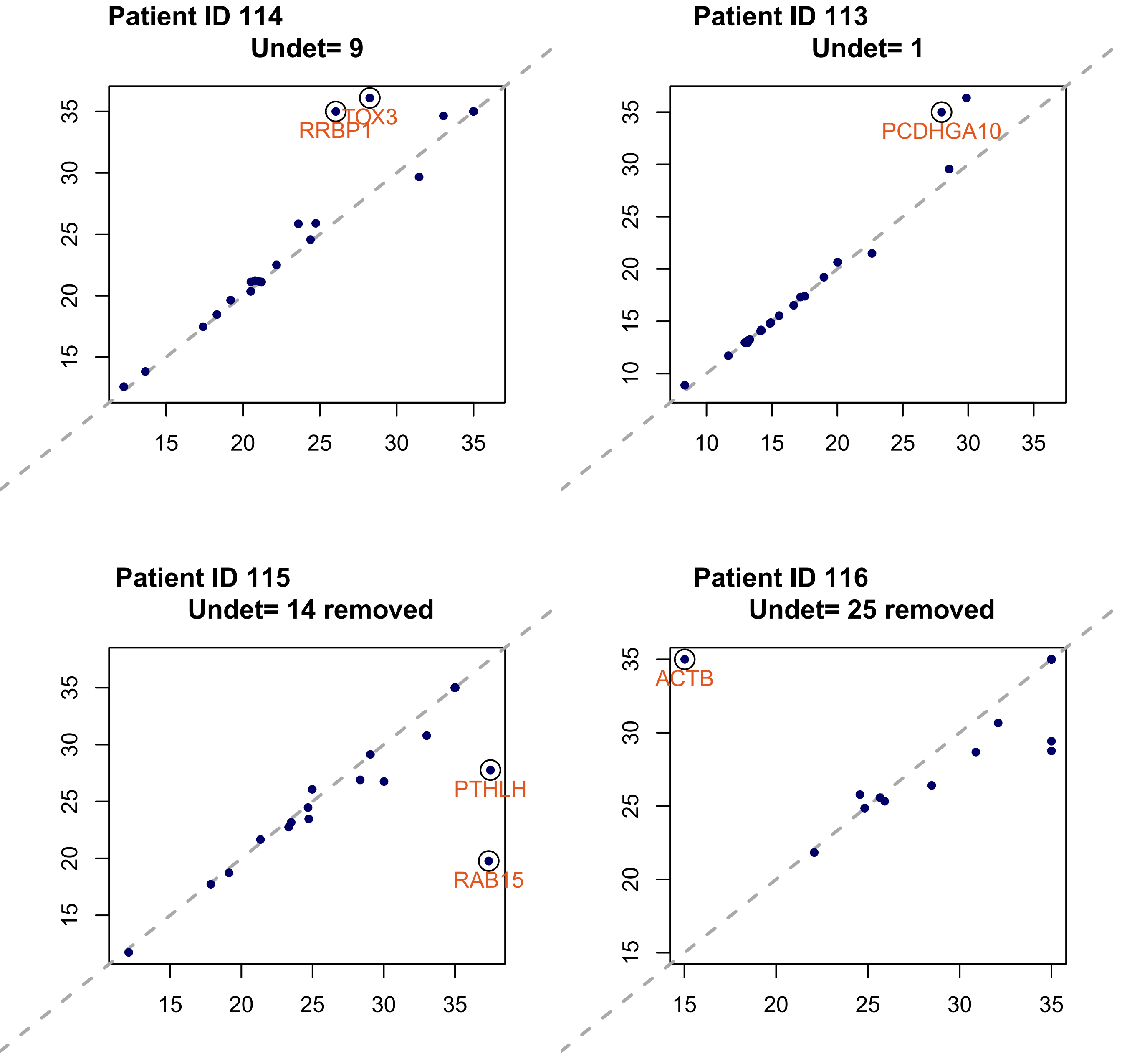
Patient ID 114
Undet= 9
TOX3
RRBP1
15
20
25
30
35
15
20
25
30
35
Patient ID 113
Undet= 1
PCDHGA10
10
15
20
25
30
35
10
15
20
25
30
35
Patient ID 115
Undet= 14 removed
PTHLH
RAB15
15
20
25
30
35
15
20
25
30
35
Patient ID 116
Undet= 25 removed
ACTB
15
20
25
30
35
15
20
25
30
35

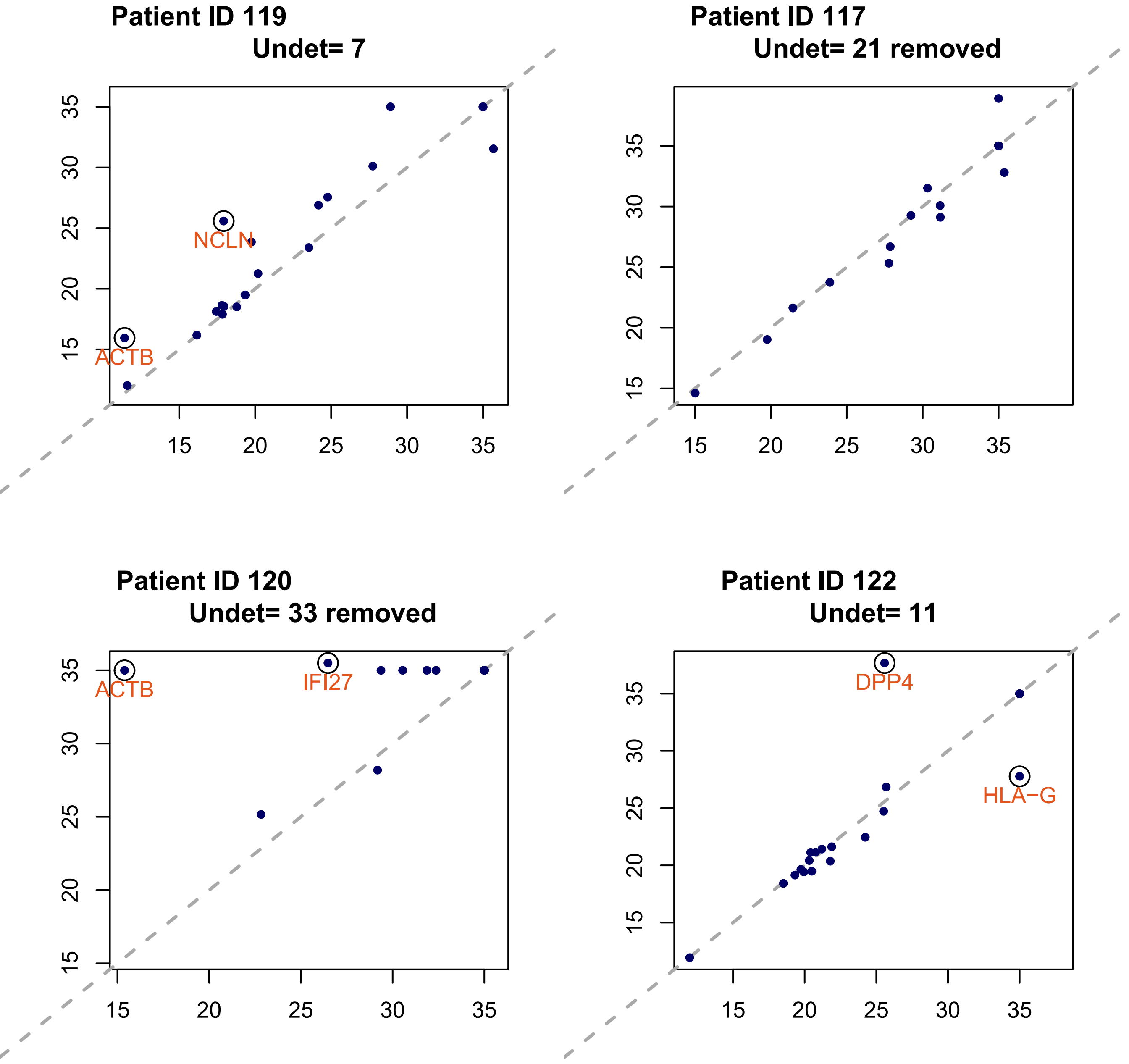
Patient ID 119
Undet= 7
NCLN
ACTB
Patient ID 117
Undet= 21 removed
Patient ID 120
Undet= 33 removed
ACTB
IFI27
Patient ID 122
Undet= 11
DPP4
HLA−G

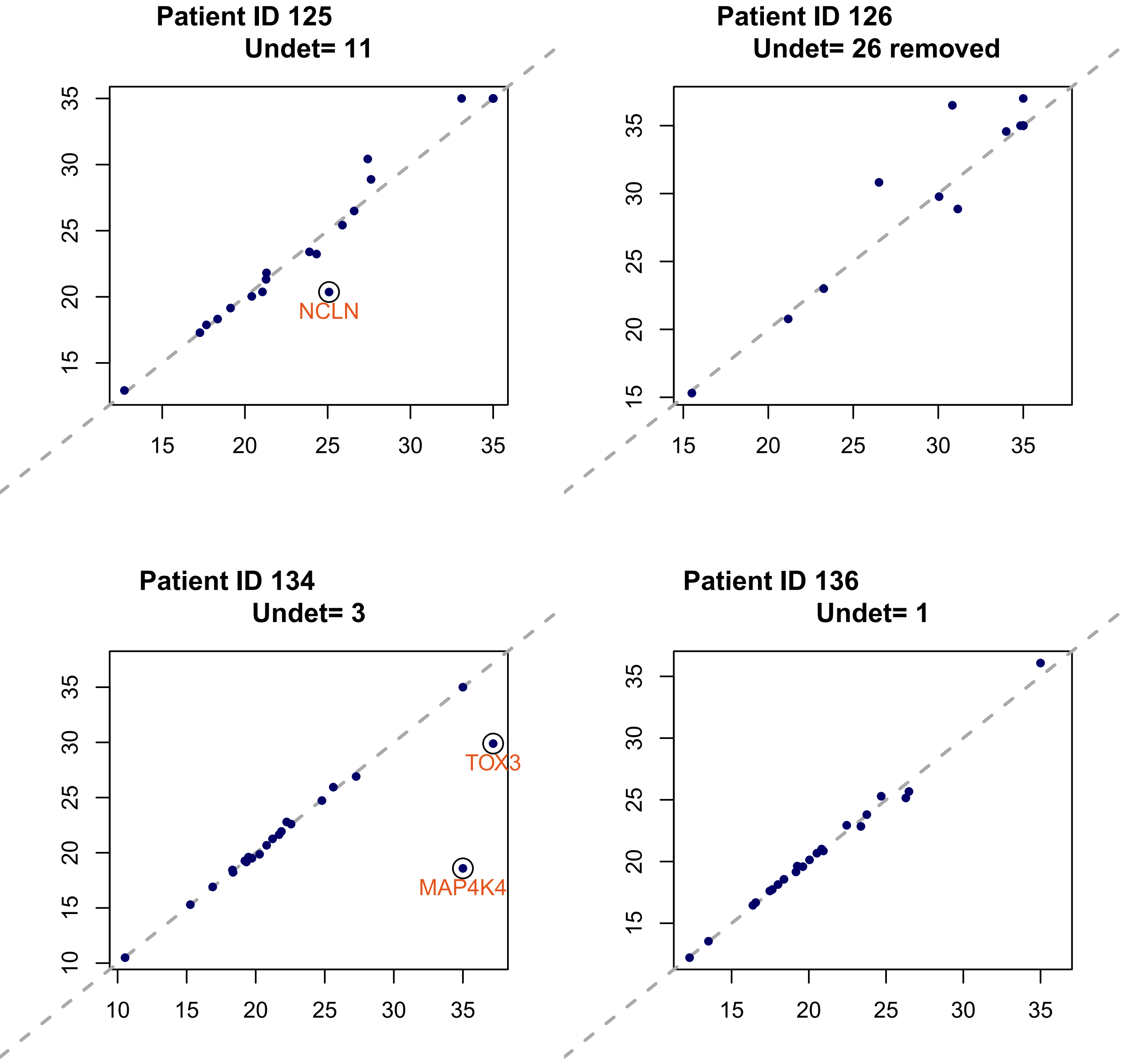
Patient ID 125
Undet= 11
NCLN
15
20
25
30
35
15
20
25
30
35
Patient ID 126
Undet= 26 removed
15
20
25
30
35
15
20
25
30
35
Patient ID 134
Undet= 3
TOX3
MAP4K4
10
15
20
25
30
35
10
15
20
25
30
35
Patient ID 136
Undet= 1
15
20
25
30
35
15
20
25
30
35

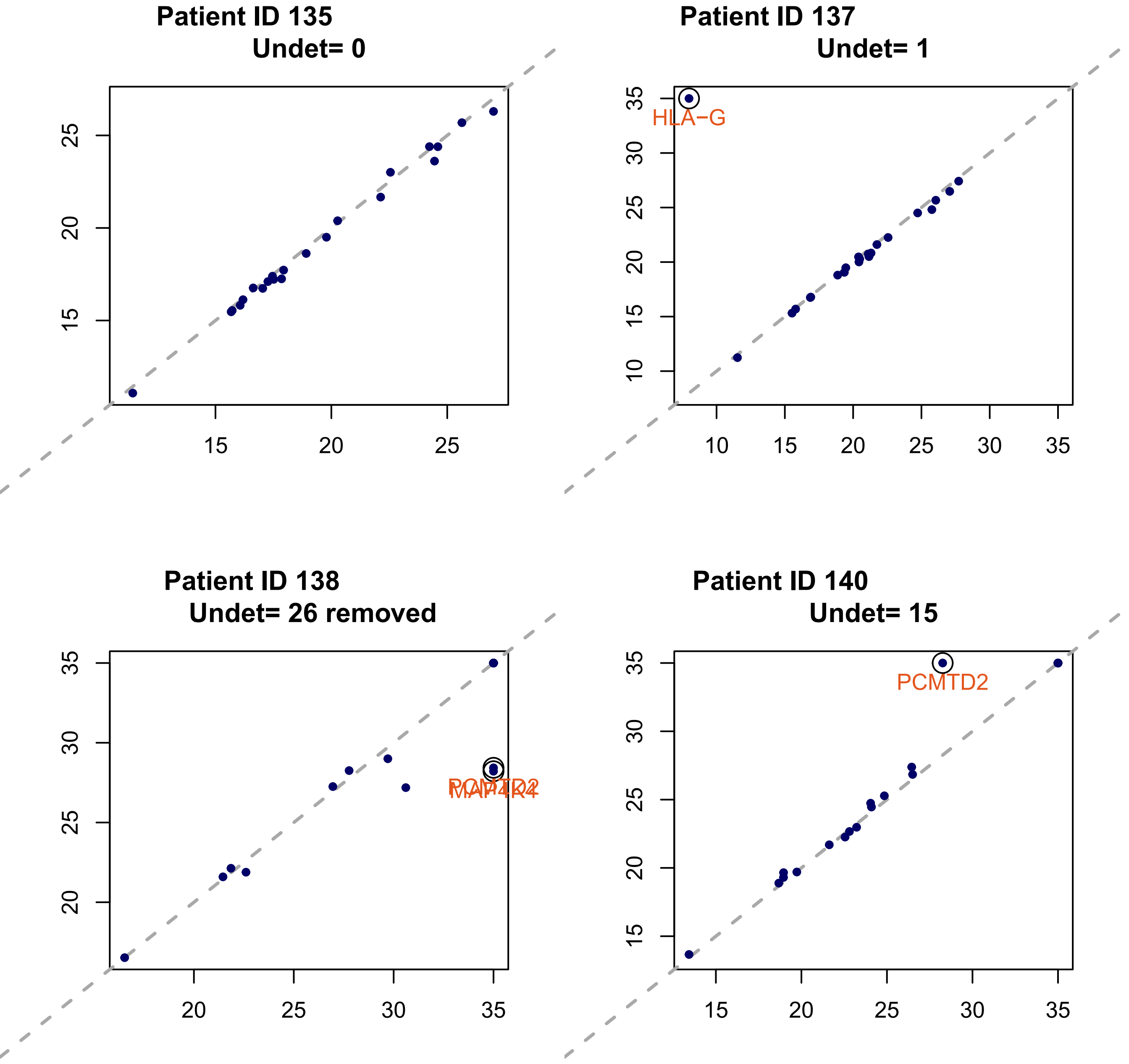

Patient ID 135
Undet= 0
Patient ID 137
Undet= 1
HLA−G
Patient ID 138
Undet= 26 removed
PCMTD2
MAP4K4
Patient ID 140
Undet= 15
PCMTD2

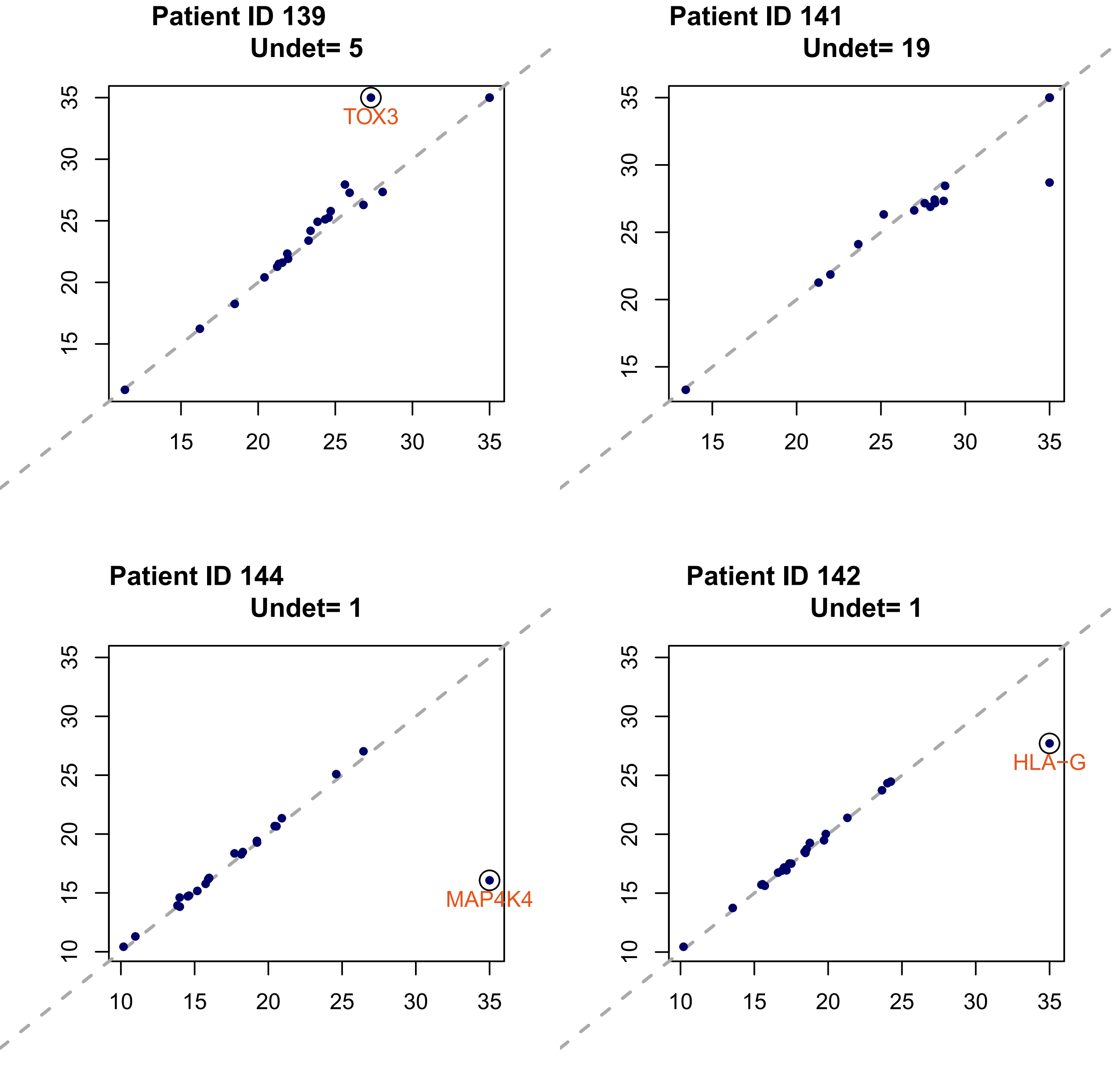

Patient ID 139
Undet= 5
TOX3
Patient ID 141
Undet= 19
Patient ID 144
Undet= 1
MAP4K4
Patient ID 142
Undet= 1
HLA-G
10
15
20
25
30
35

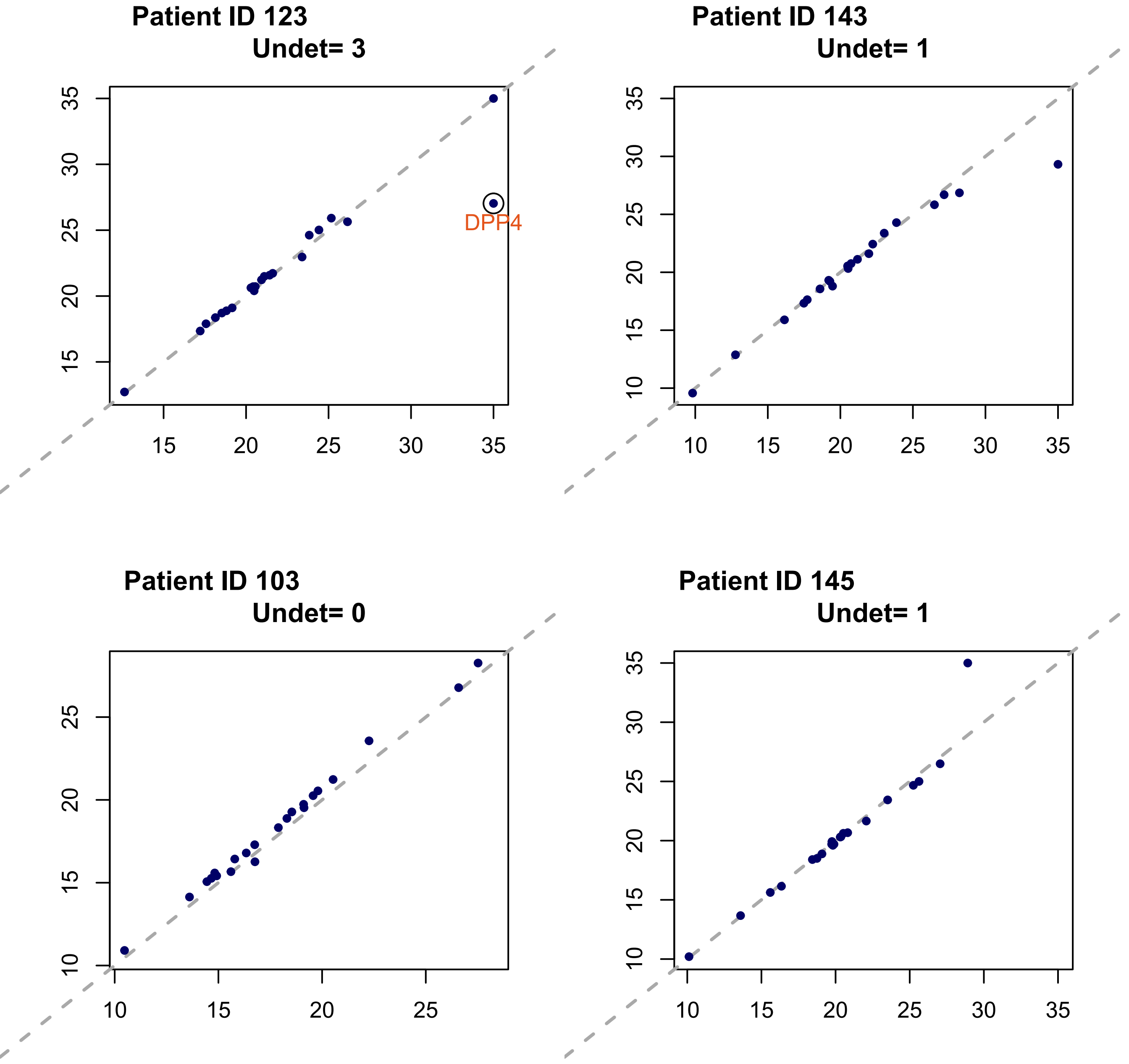

Patient ID 123
Undet= 3
DPP4
Patient ID 143
Undet= 1
Patient ID 103
Undet= 0
Patient ID 145
Undet= 1

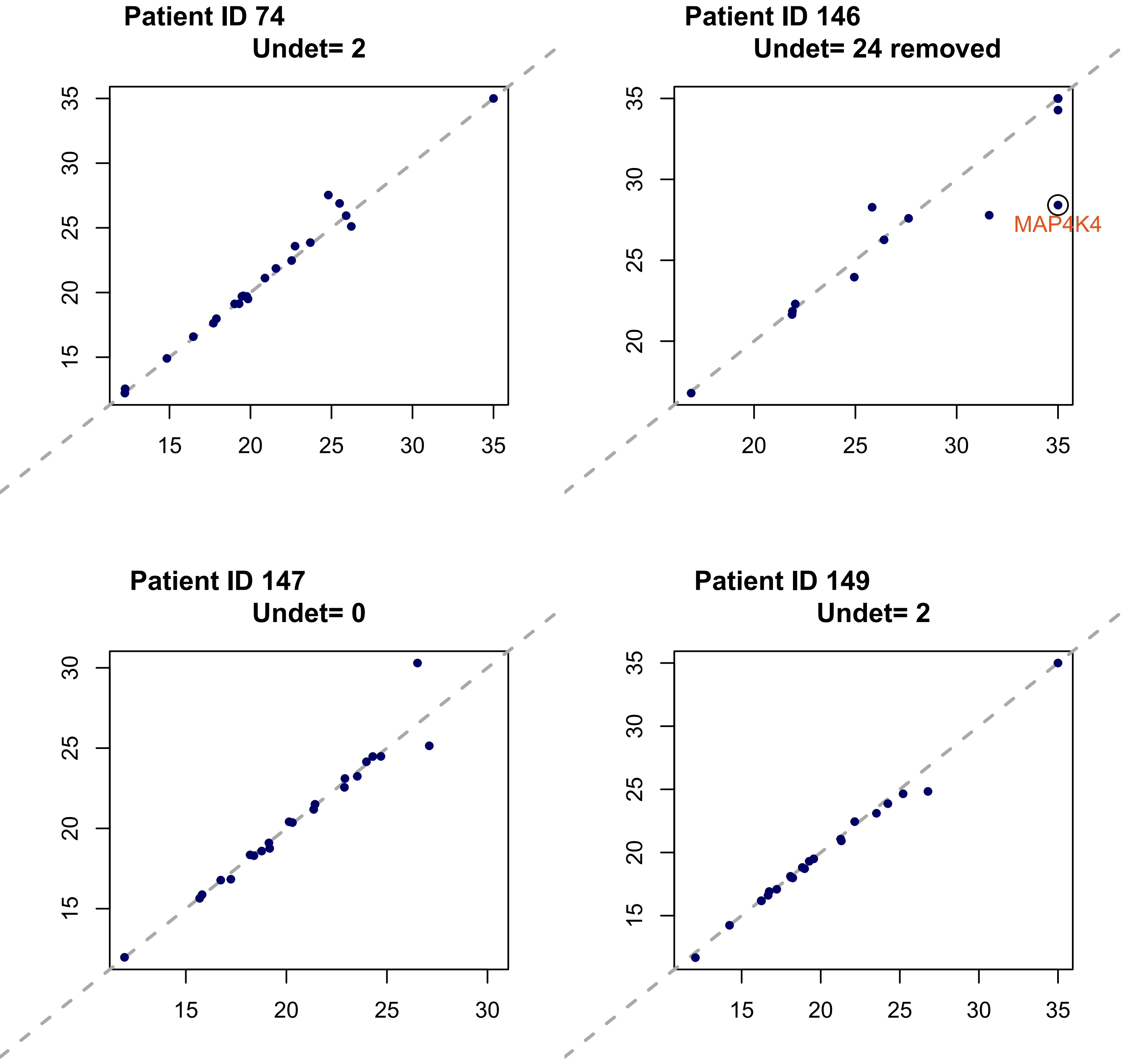
Patient ID 74
Undet= 2
Patient ID 146
Undet= 24 removed
MAP4K4
Patient ID 147
Undet= 0
Patient ID 149
Undet= 2
15
20
25
30
35

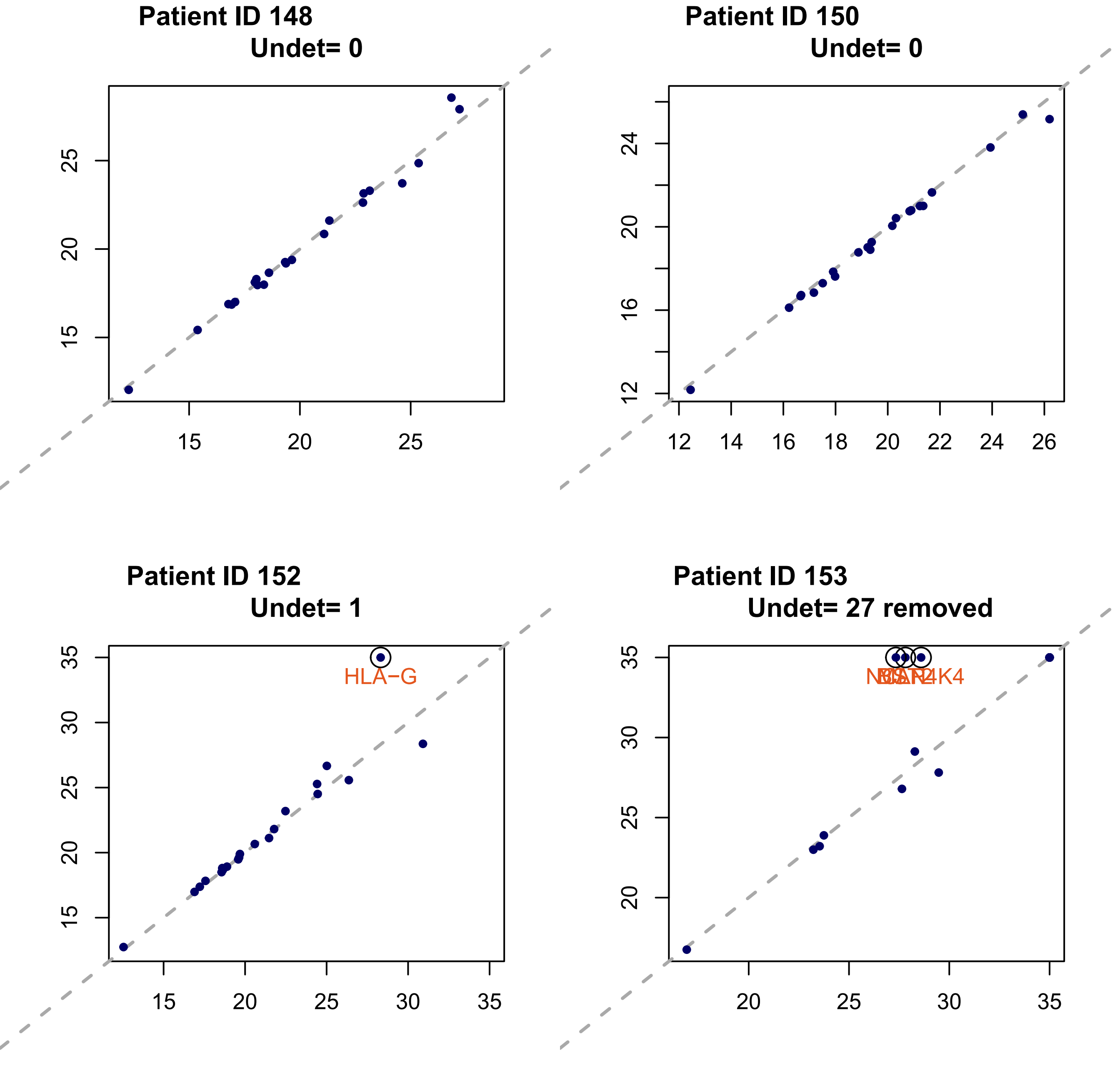

Patient ID 148
Undet= 0
15 20 25
15 20 25
Patient ID 150
Undet= 0
12 14 16 18 20 22 24 26
12 16 20 24
Patient ID 152
Undet= 1
HLA−G
15 20 25 30 35
15 20 25 30 35
Patient ID 153
Undet= 27 removed
20 25 30 35
20 25 30 35

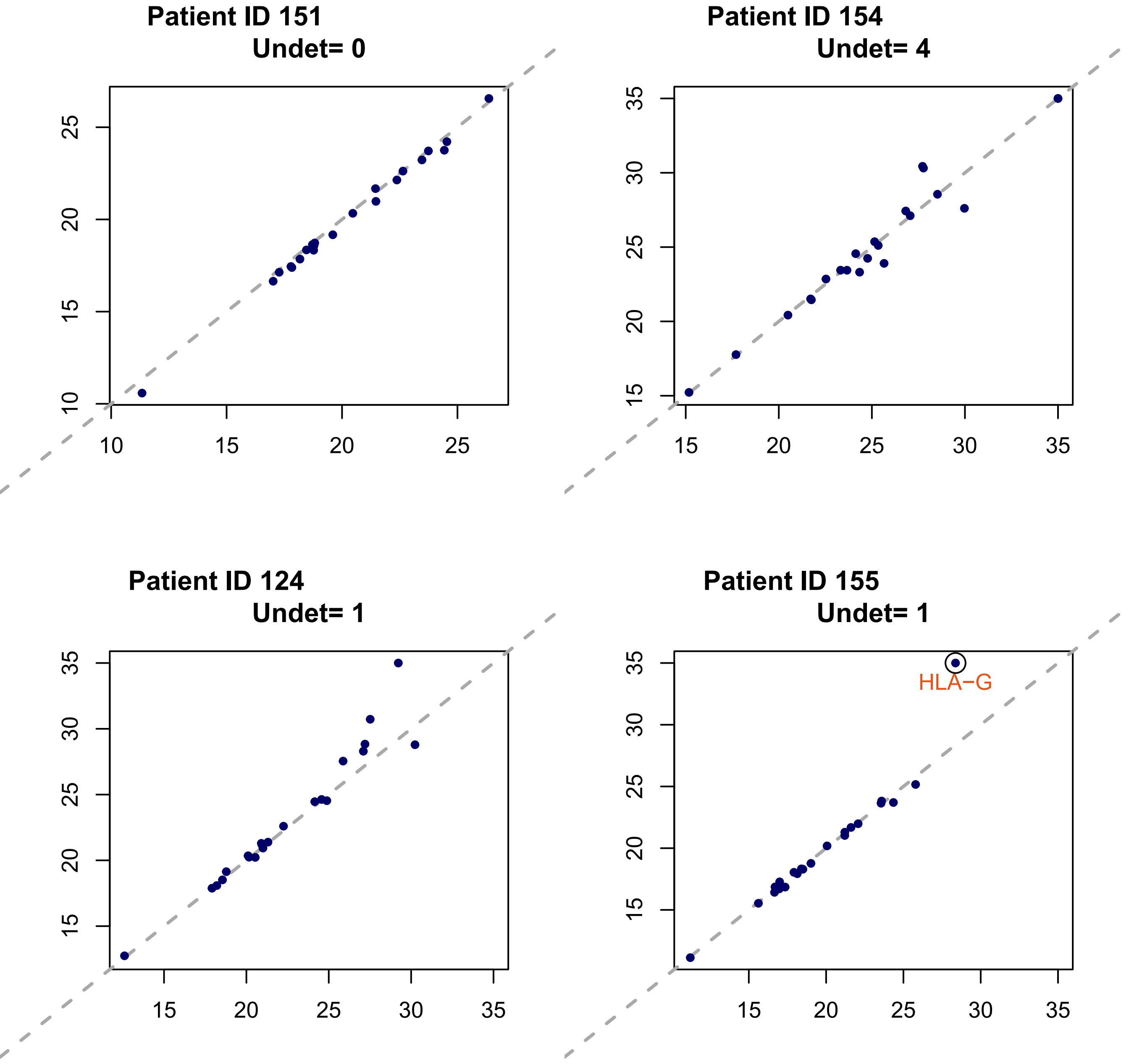
Patient ID 151
Undet= 0
Patient ID 154
Undet= 4
Patient ID 124
Undet= 1
Patient ID 155
Undet= 1
HLA−G

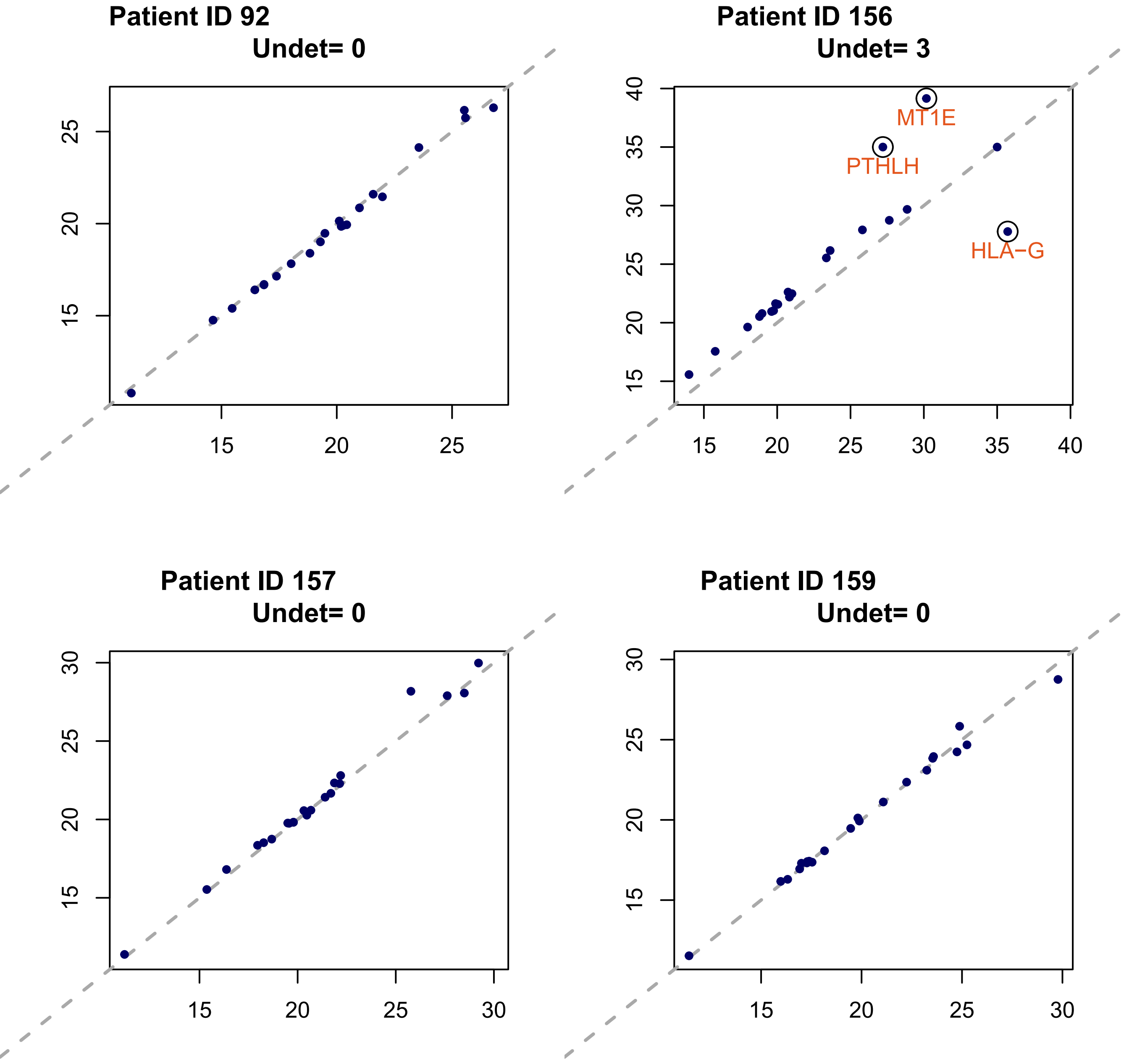

Patient ID 92
Undet= 0
Patient ID 156
Undet= 3
MT1E
PTHLH
HLA−G
Patient ID 157
Undet= 0
Patient ID 159
Undet= 0

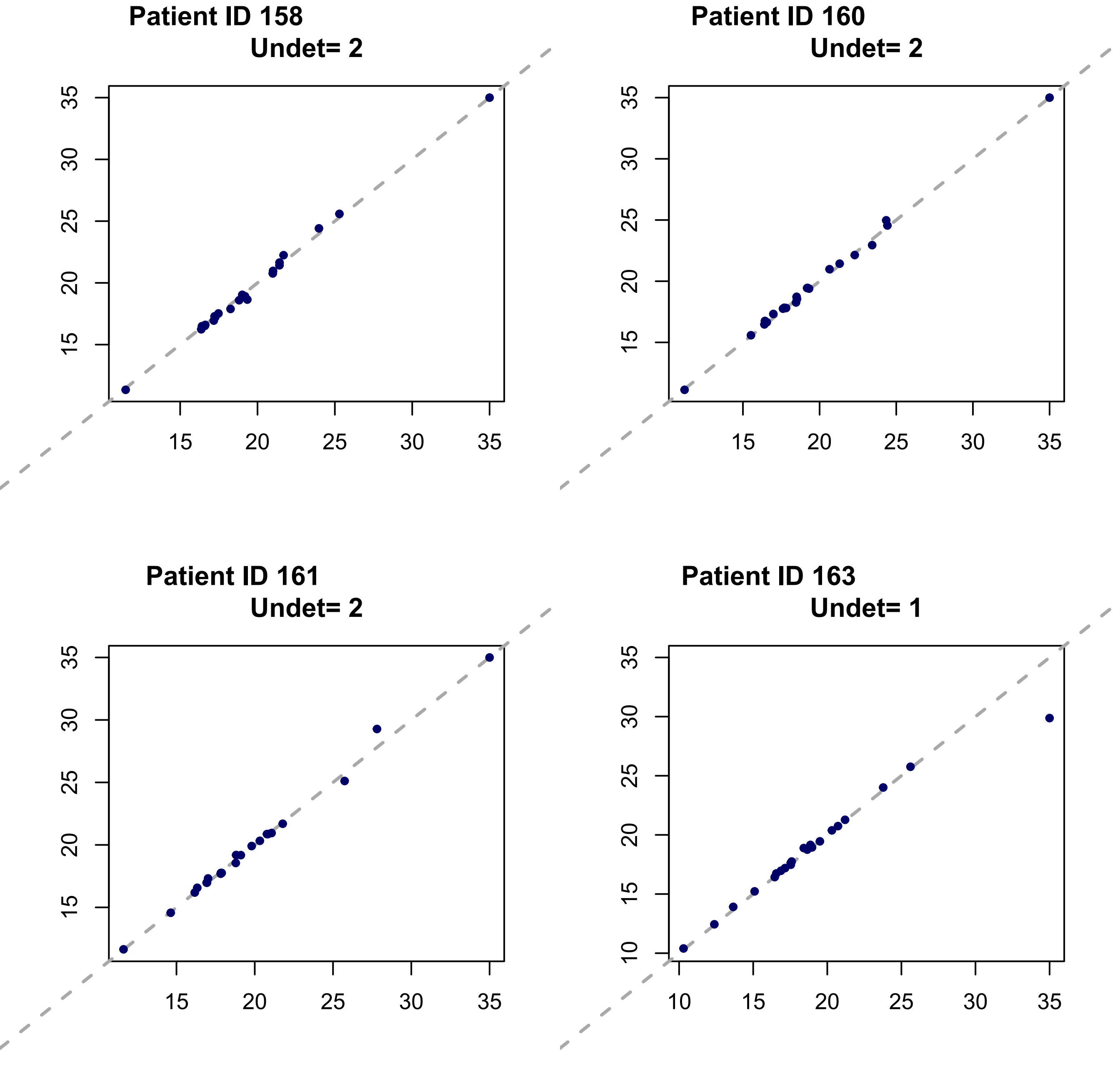
Patient ID 158
Undet= 2
Patient ID 160
Undet= 2
Patient ID 161
Undet= 2
Patient ID 163
Undet= 1
10
15
20
25
30
35

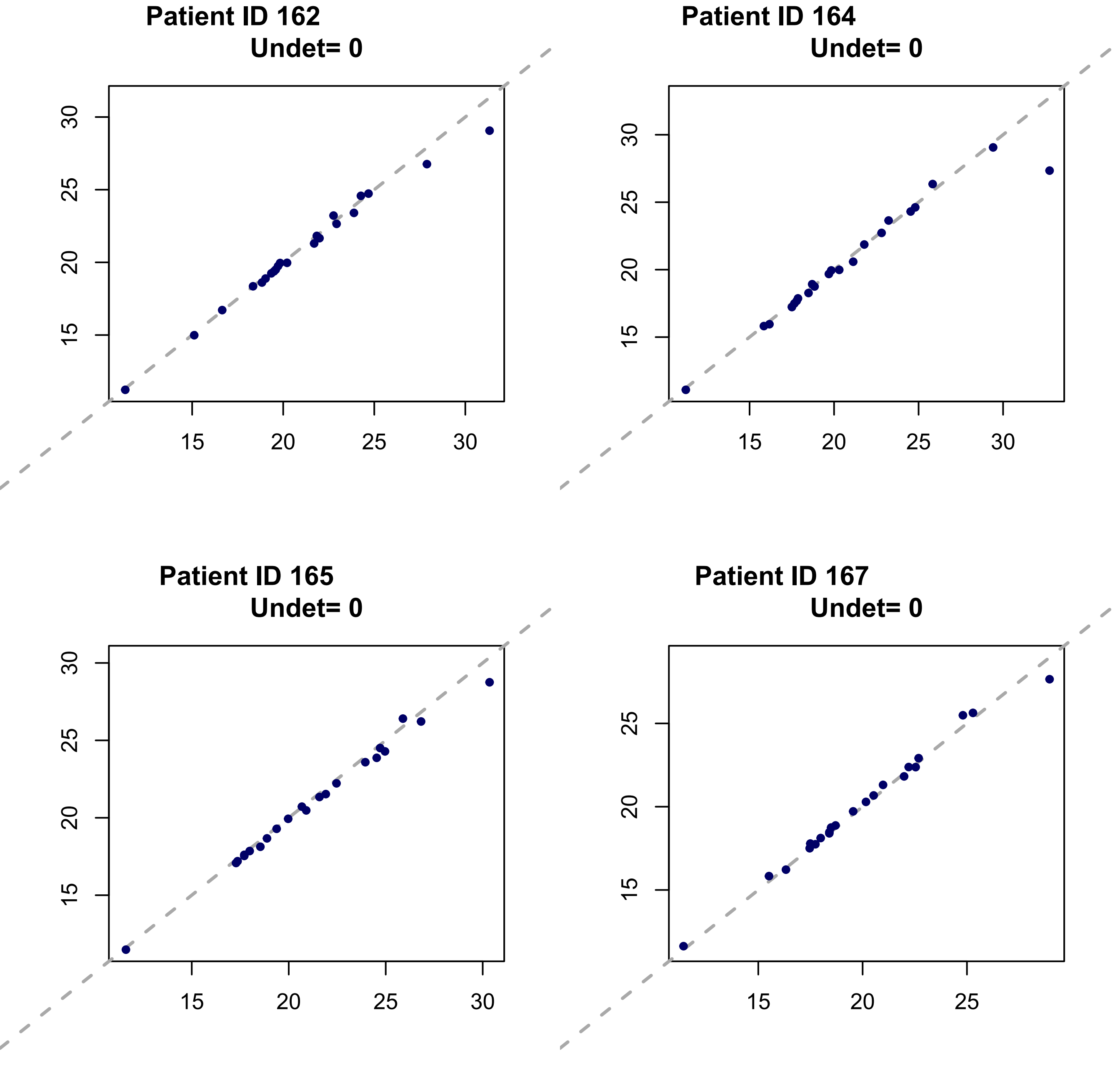
Patient ID 162
Undet= 0
Patient ID 164
Undet= 0
Patient ID 165
Undet= 0
Patient ID 167
Undet= 0

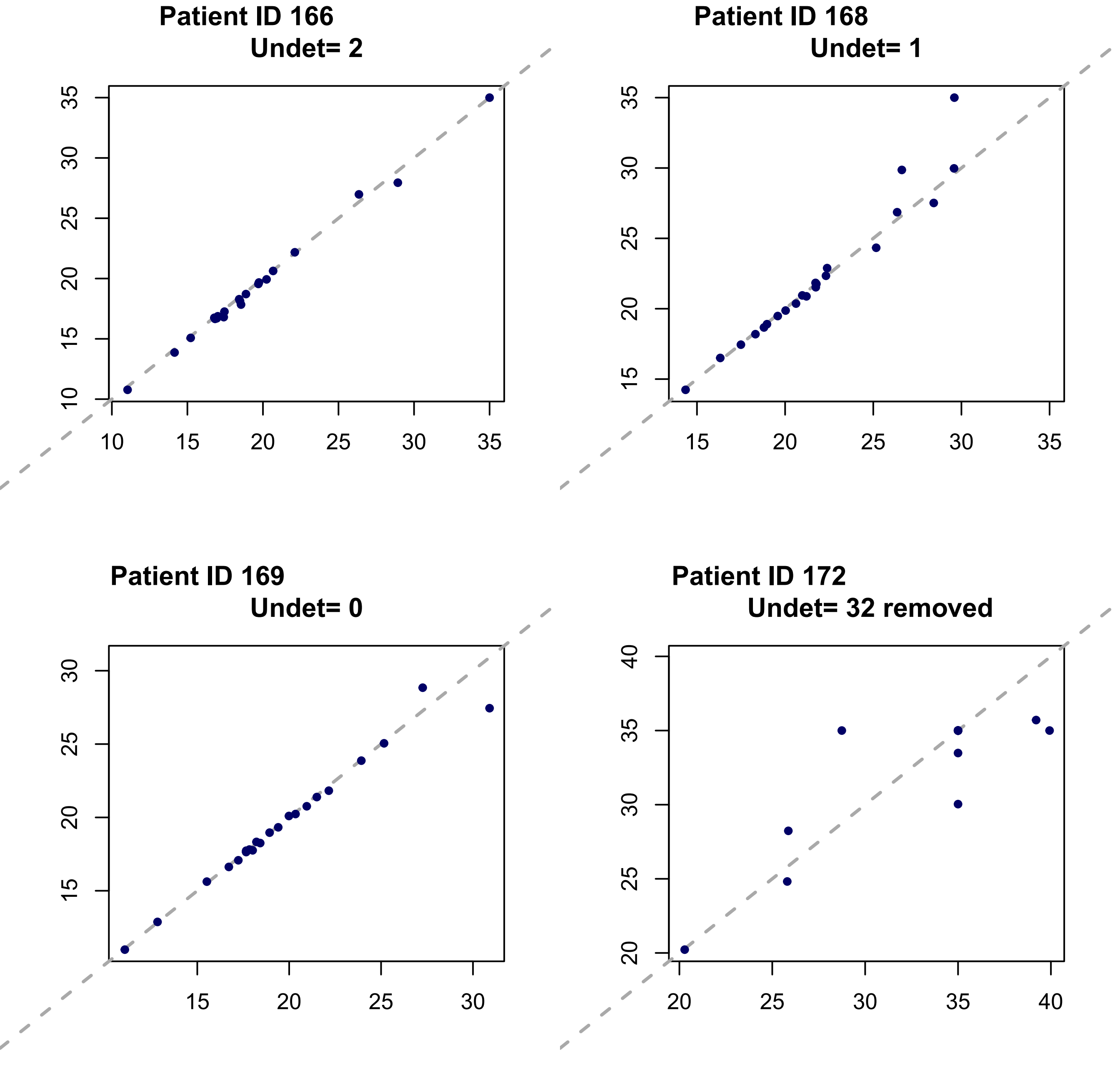
Patient ID 166
Undet= 2
10 15 20 25 30 35
10 15 20 25 30 35
Patient ID 168
Undet= 1
15 20 25 30 35
15 20 25 30 35
Patient ID 169
Undet= 0
15 20 25 30
15 20 25 30
Patient ID 172
Undet= 32 removed
20 25 30 35 40
20 25 30 35 40

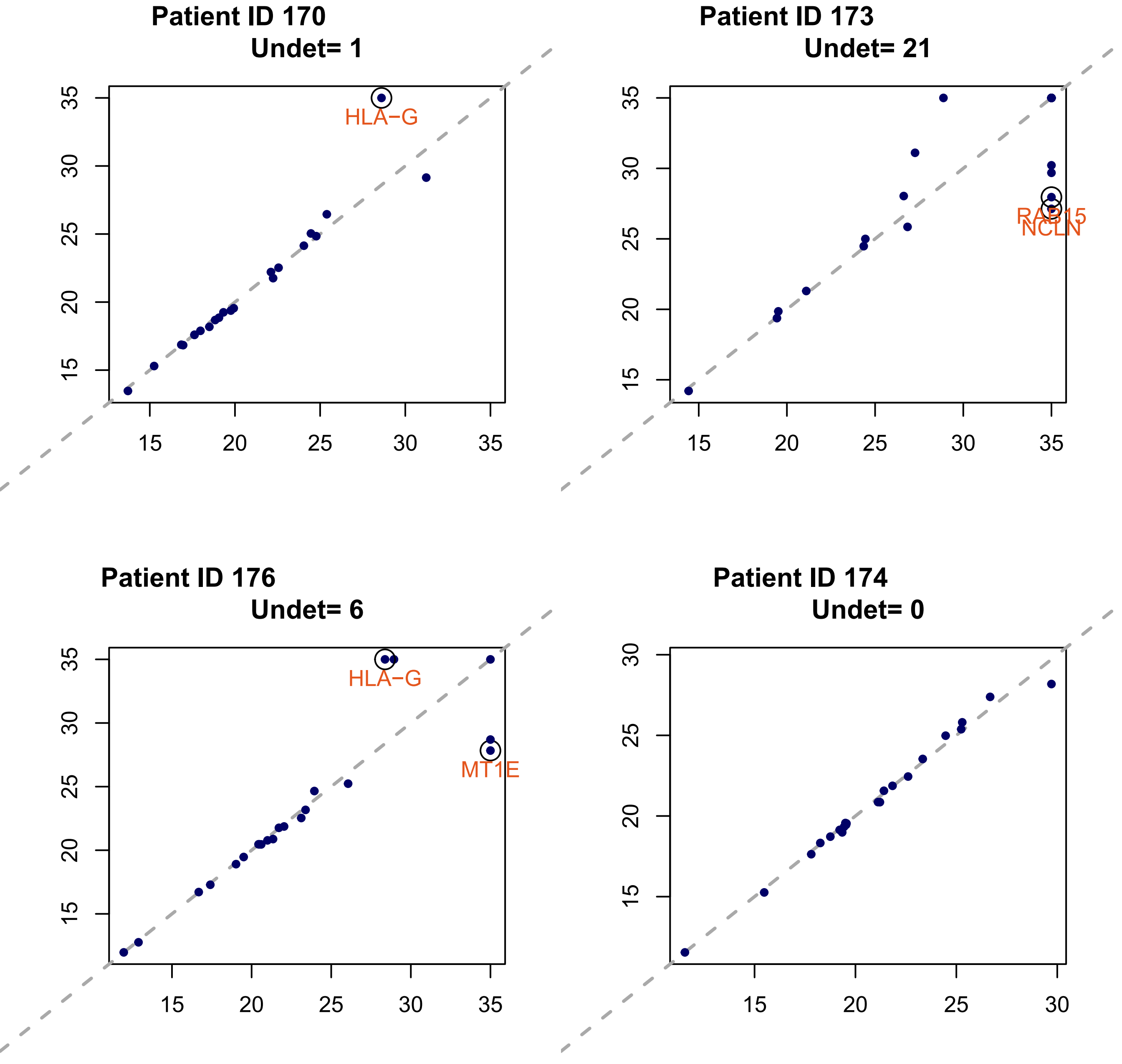
Patient ID 170
Undet= 1
HLA−G
Patient ID 173
Undet= 21
RAB15
NCLN
Patient ID 176
Undet= 6
HLA−G
MT1E
Patient ID 174
Undet= 0

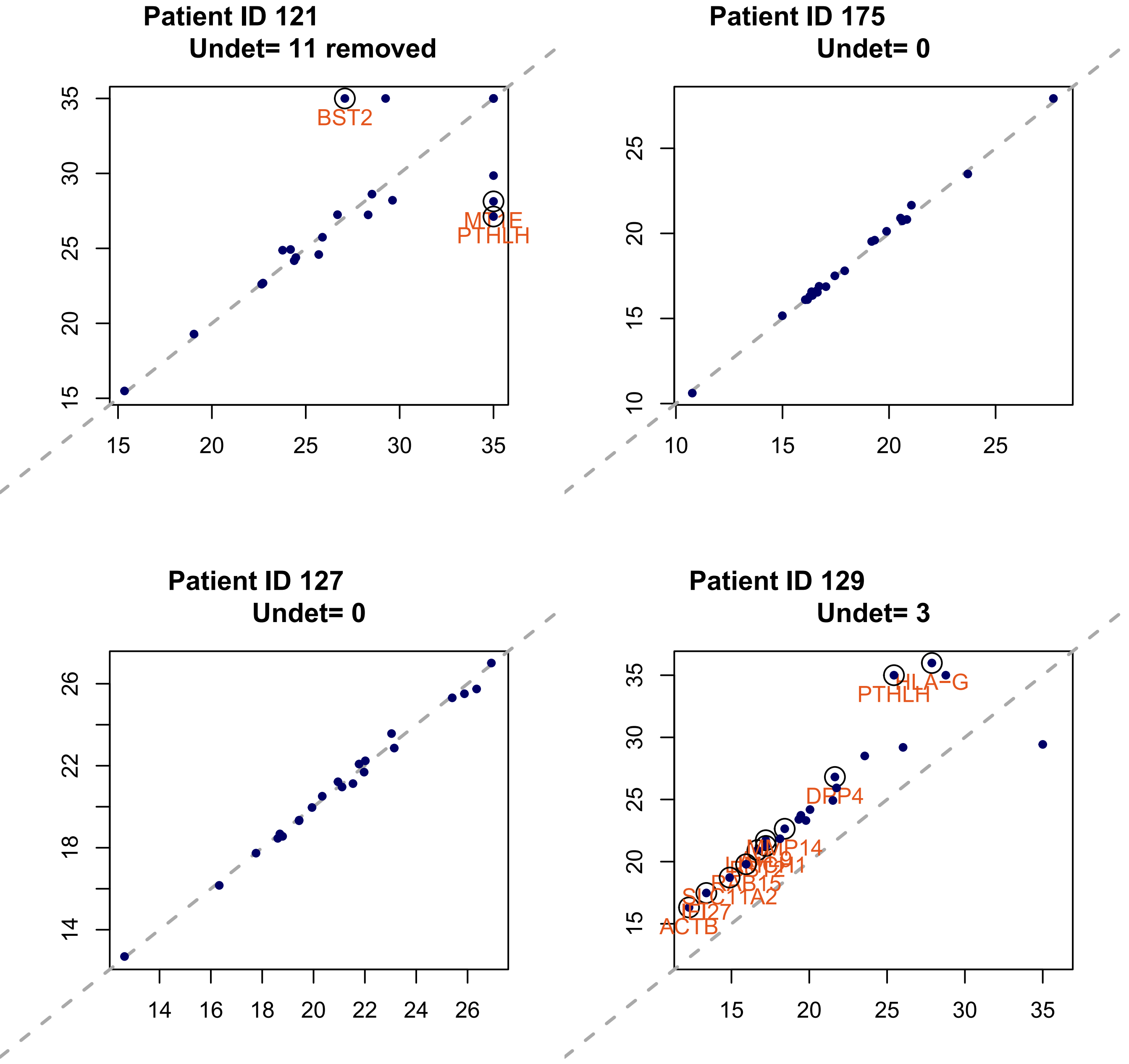
Patient ID 121
Undet= 11 removed
BST2
PTHLH
Patient ID 175
Undet= 0
Patient ID 127
Undet= 0
Patient ID 129
Undet= 3
HLA−G
PTHLH
DPP4
MMP14
RAB15
ACTB

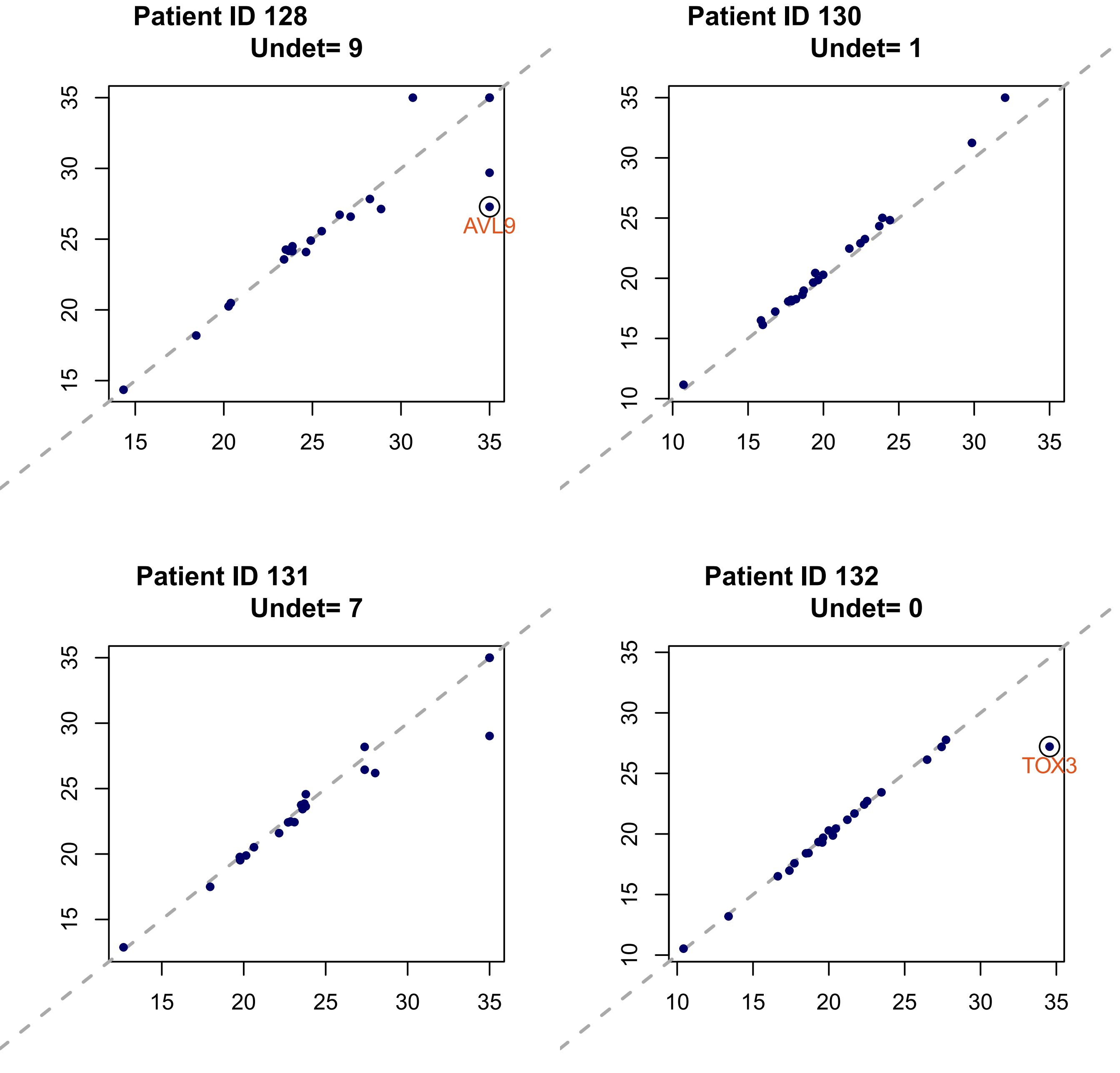

Patient ID 128
Undet= 9
AVL9
Patient ID 130
Undet= 1
Patient ID 131
Undet= 7
Patient ID 132
Undet= 0
TOX3
