# Supplementary material for: A reported 20-gene expression signature to predict lymph node-positive disease at radical cystectomy for muscle-invasive bladder cancer is clinically not applicable
Source: PLoS One. 2017 Mar 20;12(3):e0174039. doi: 10.1371/journal.pone.0174039 (PMC5358850; doi:10.1371/journal.pone.0174039)
Supplement: S3 Fig — The box indicates the upper and lower quartiles of distribution, with the solid line indicating the median and the dotted line indicating the mean ΔCt value. (PDF) [file pone.0174039.s003.pdf]

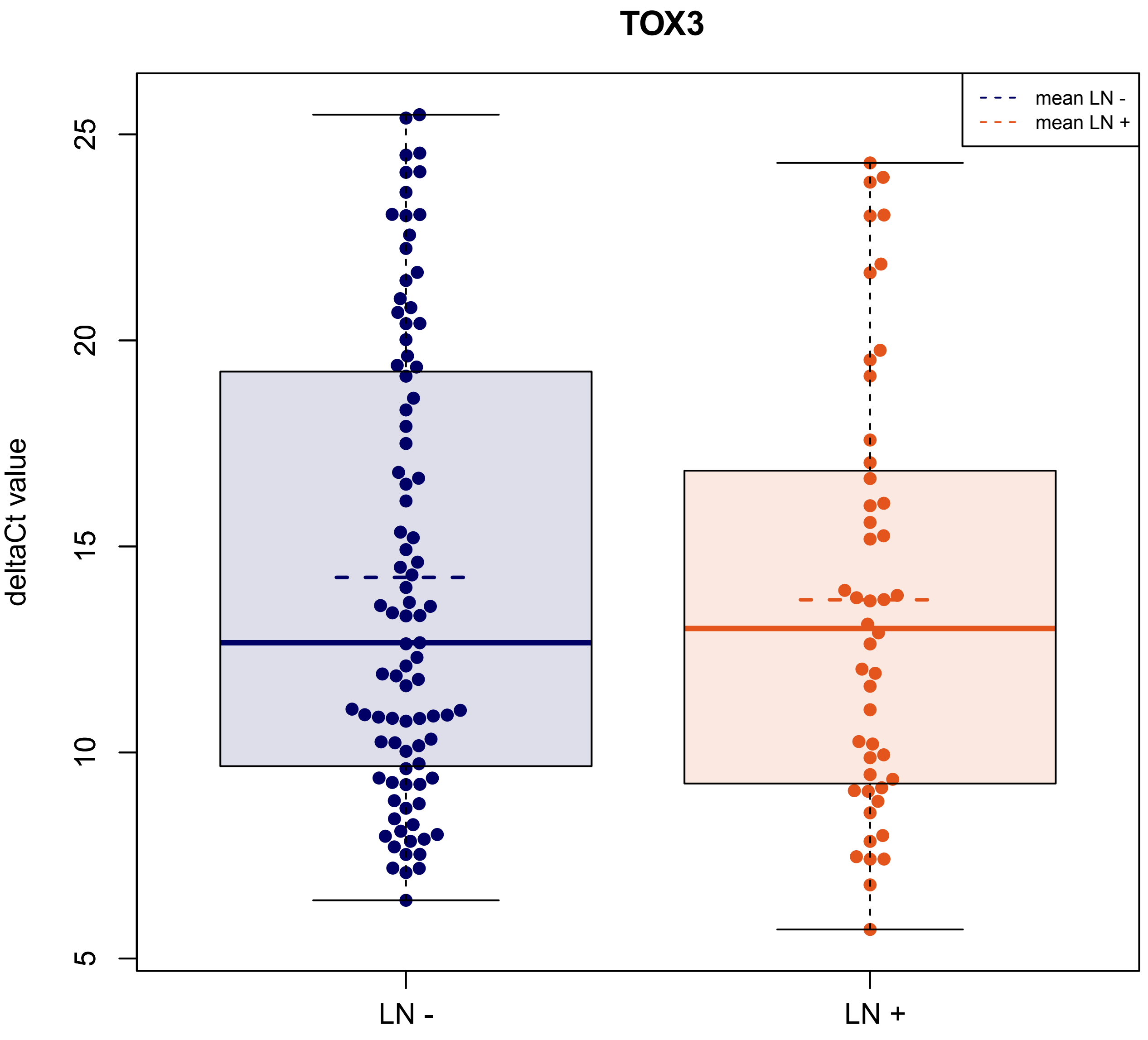

TOX3
mean LN -
mean LN +
deltaCt value
25
20
15
10
5
LN -
LN +

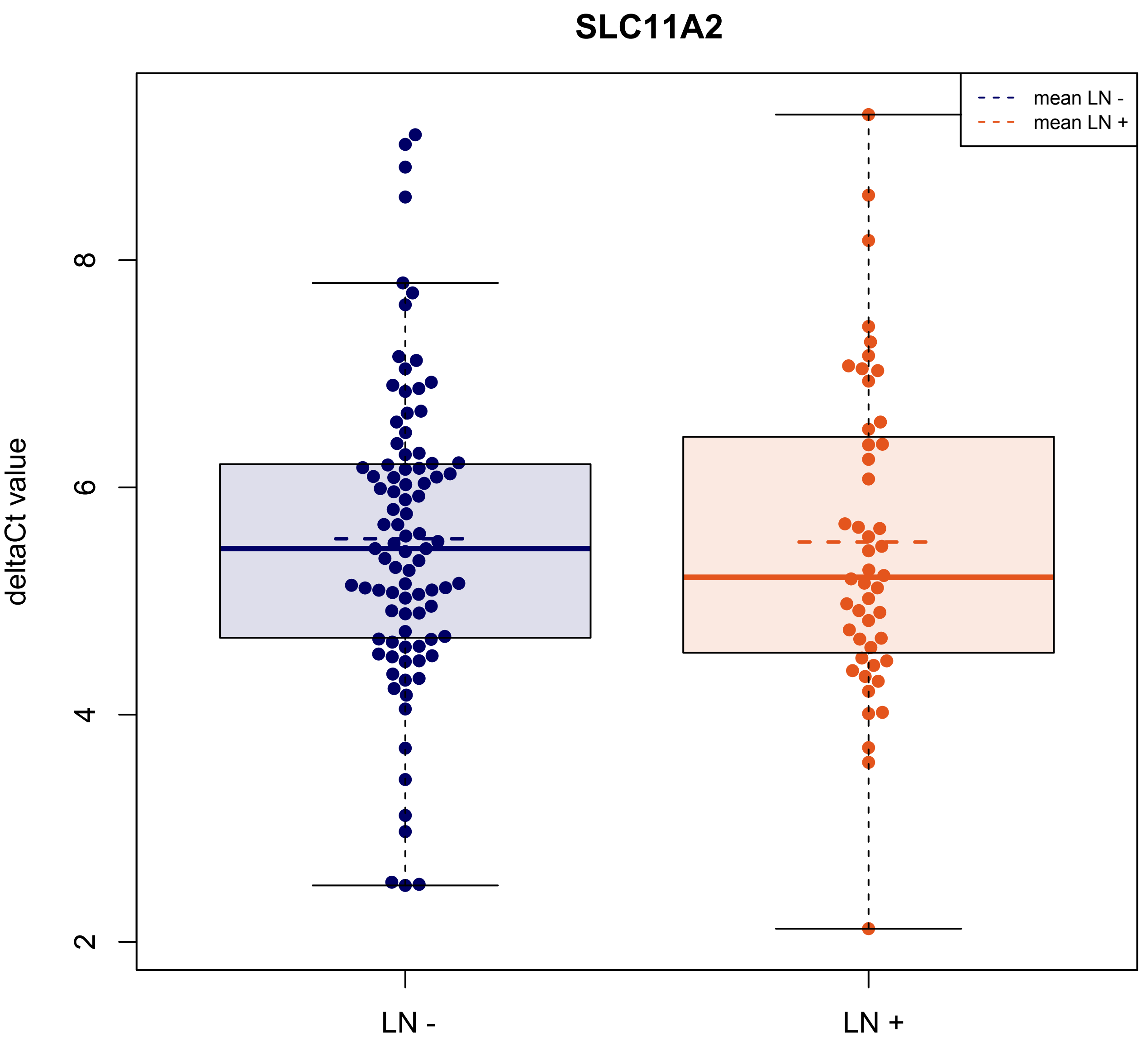

SLC11A2
mean LN -
mean LN +
deltaCt value
2
4
6
8
LN -
LN +

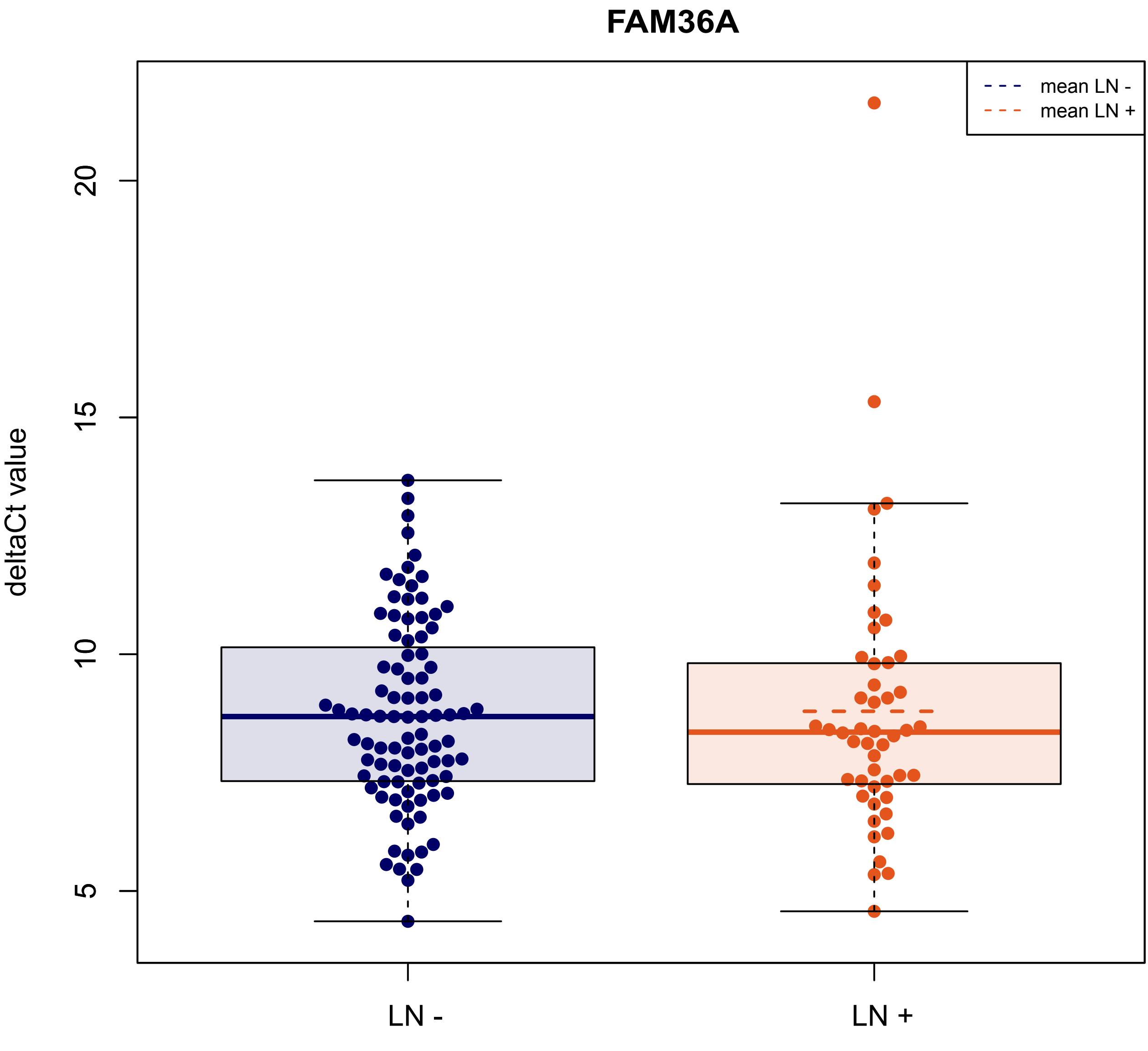
FAM36A
mean LN -
mean LN +
deltaCt value
20
15
10
5
LN -
LN +

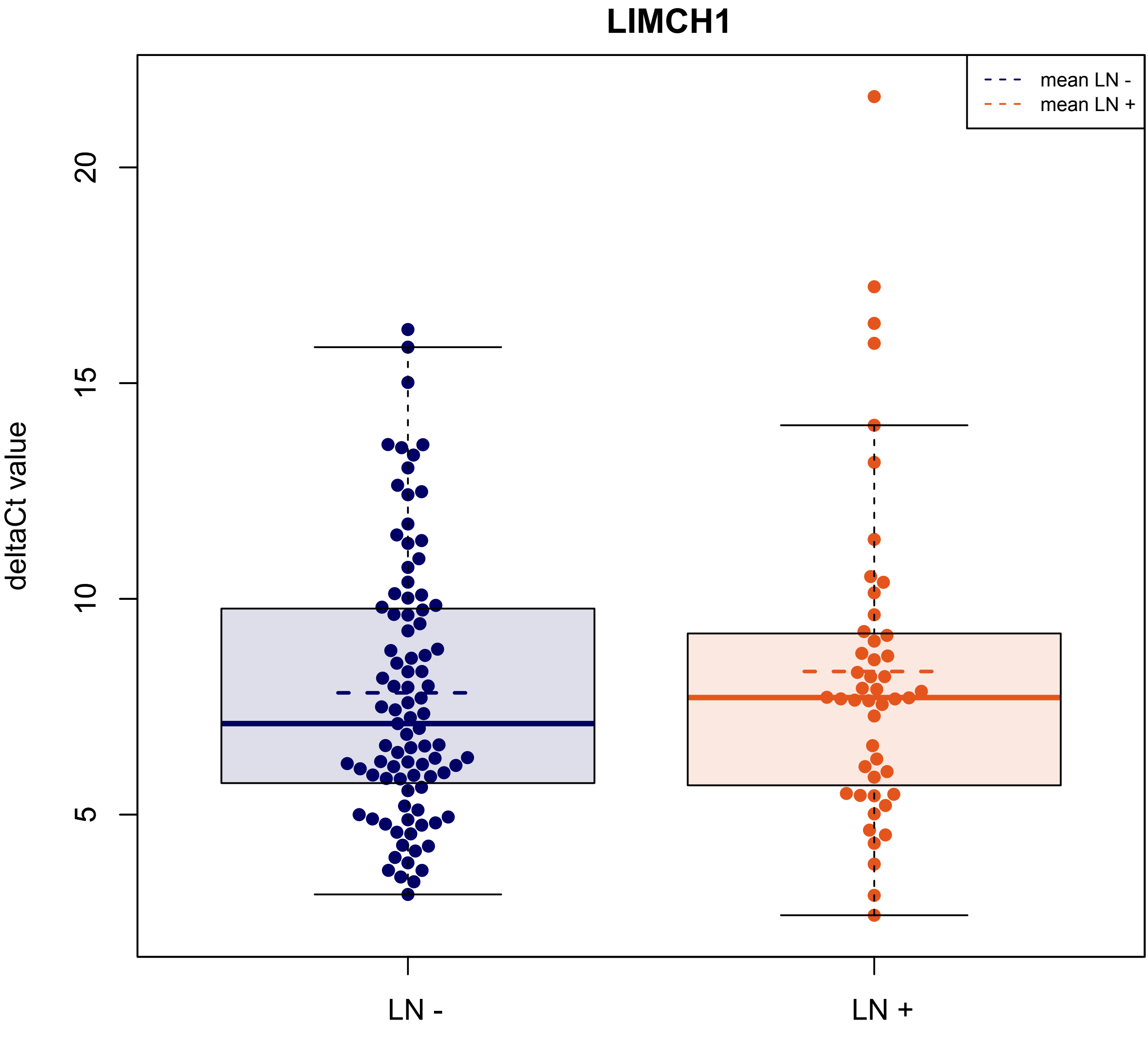
LIMCH1
mean LN -
mean LN +
deltaCt value
20
15
10
5
LN -
LN +

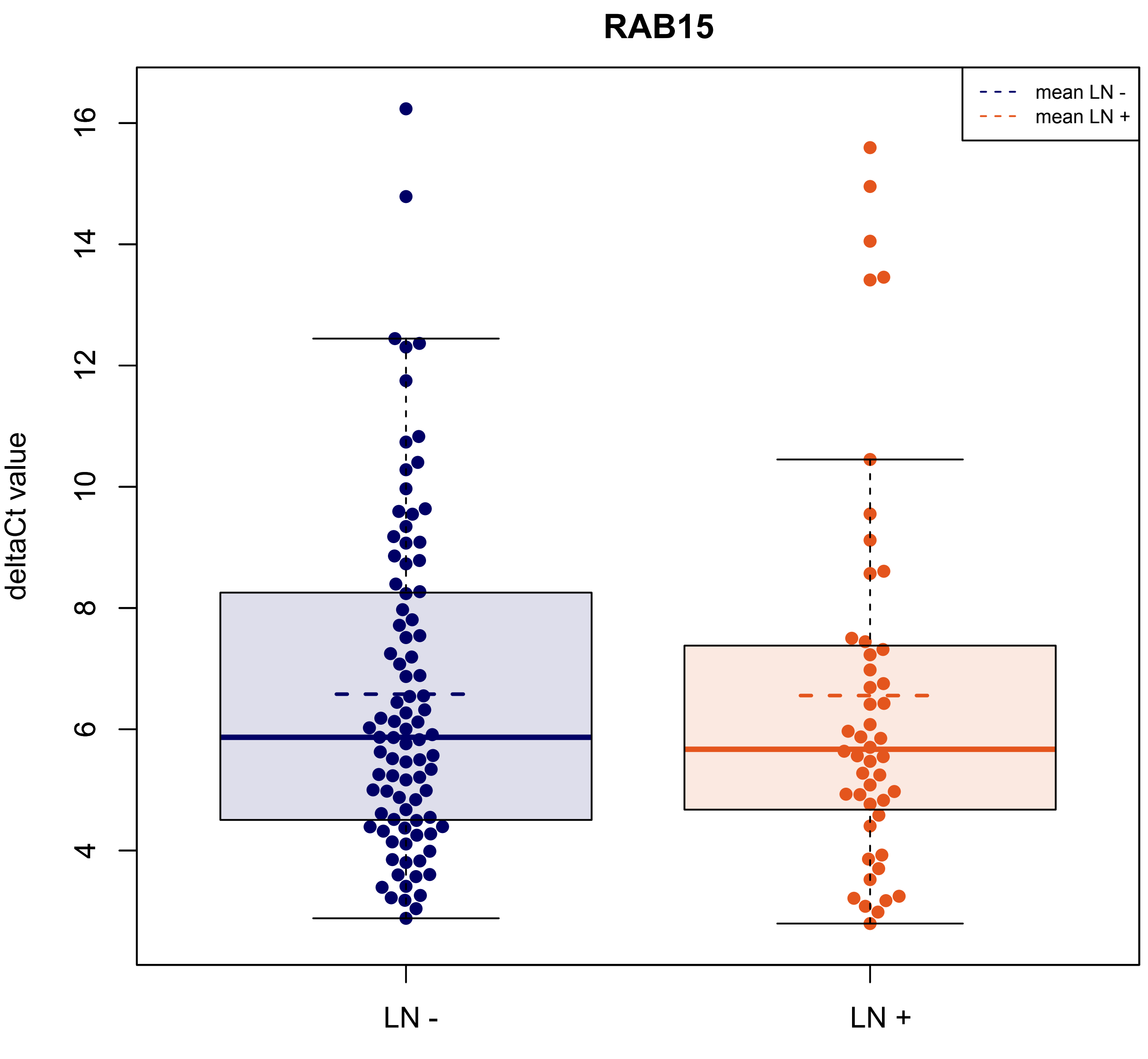
RAB15
deltaCt value
4
6
8
10
12
14
16
LN -
LN +
mean LN -
mean LN +

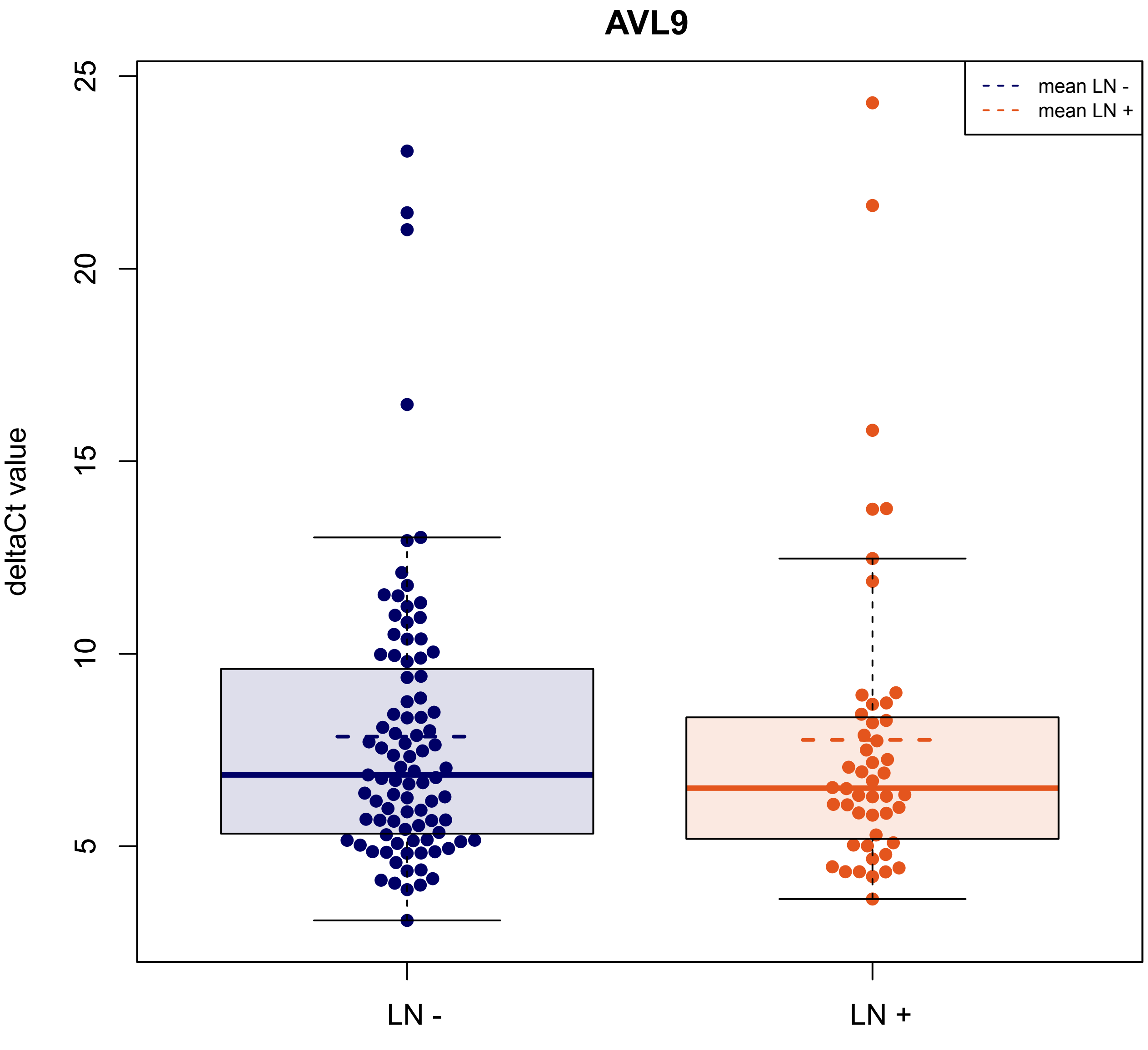

AVL9
mean LN -
mean LN +
25
20
15
10
5
deltaCt value
LN -
LN +

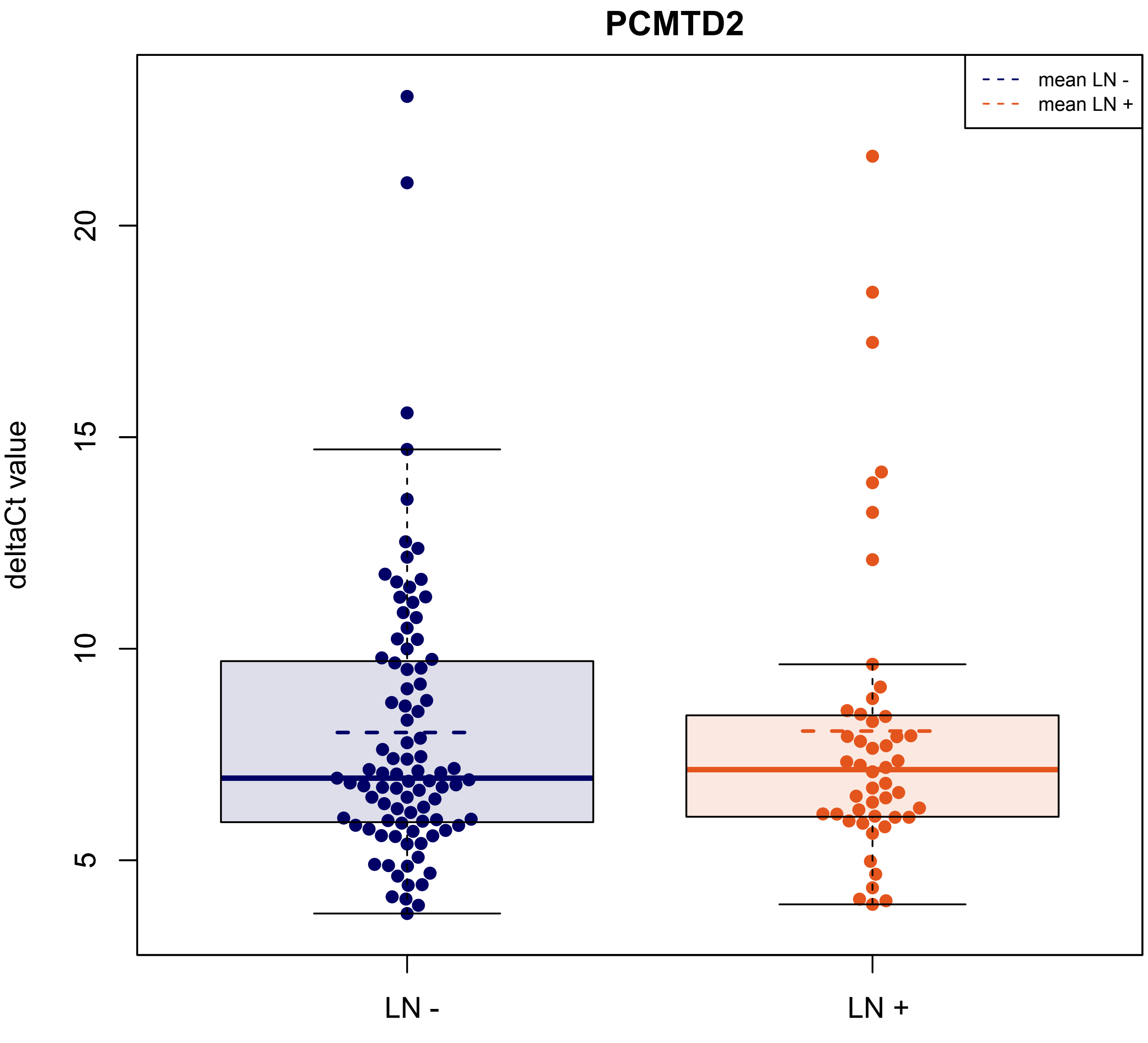

PCMTD2
mean LN -
mean LN +
deltaCt value
5
10
15
20
LN -
LN +

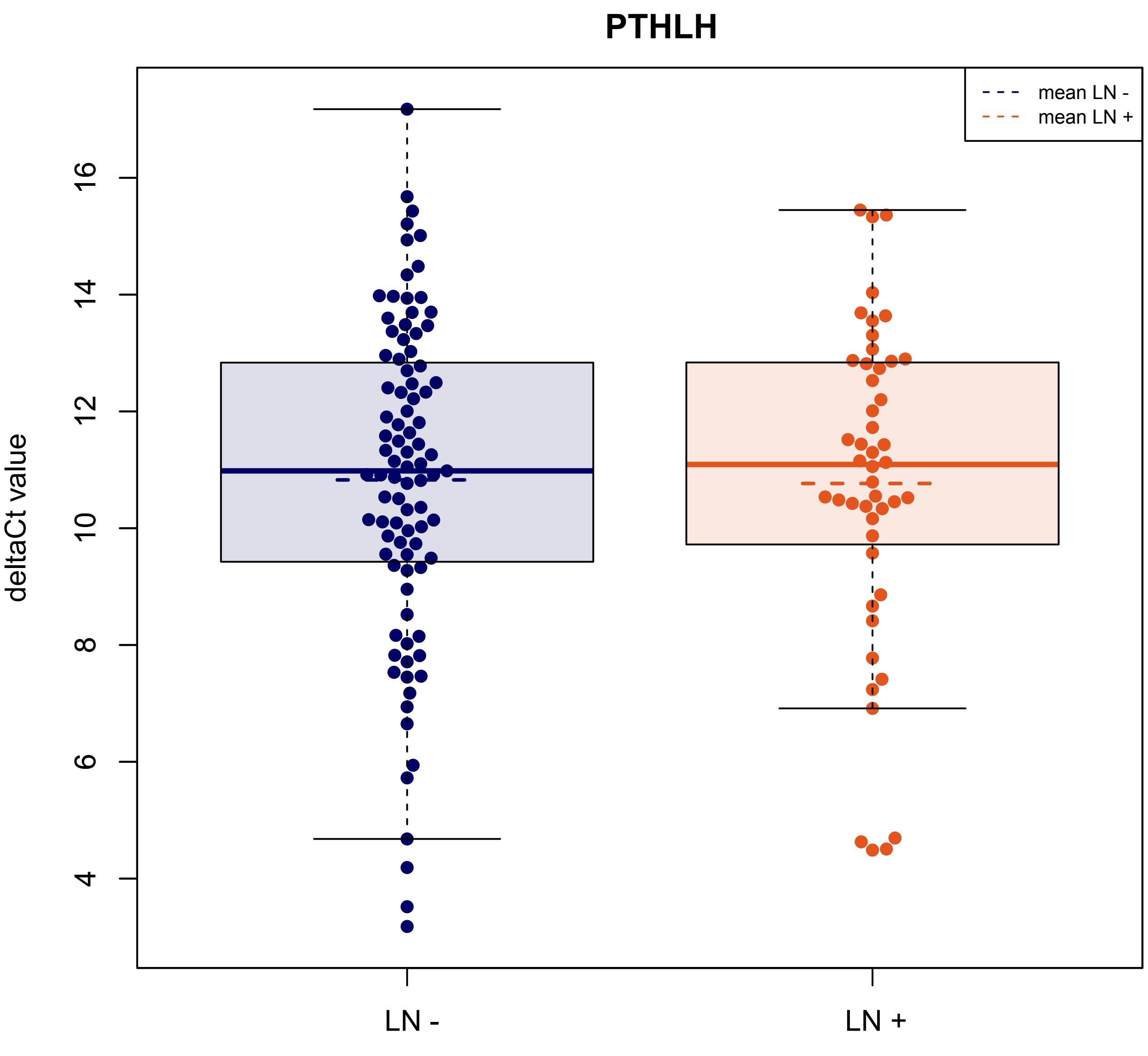

PTHLH
mean LN -
mean LN +
deltaCt value
4
6
8
10
12
14
16
LN -
LN +

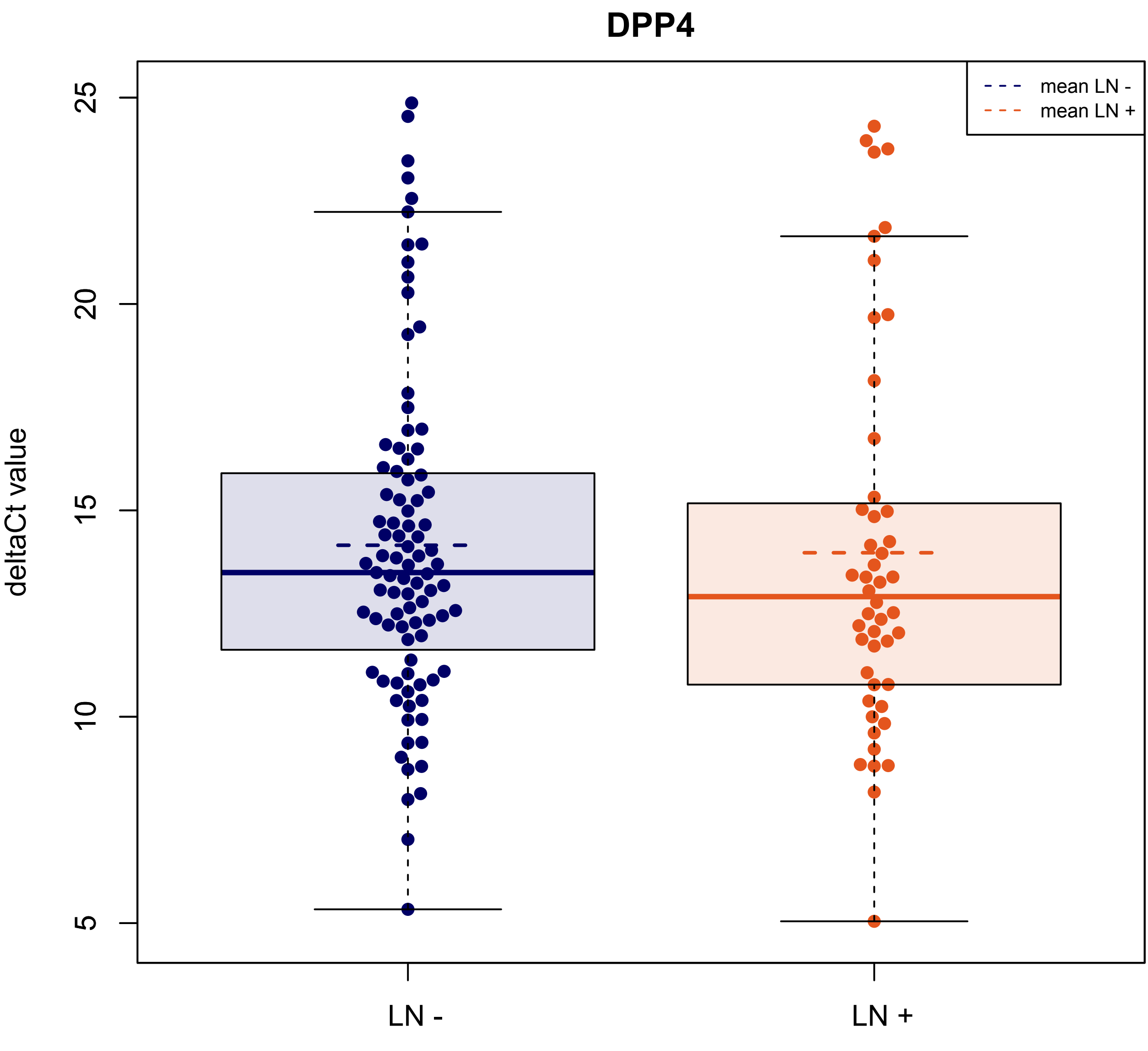

DPP4
mean LN -
mean LN +
deltaCt value
25
20
15
10
5
LN -
LN +

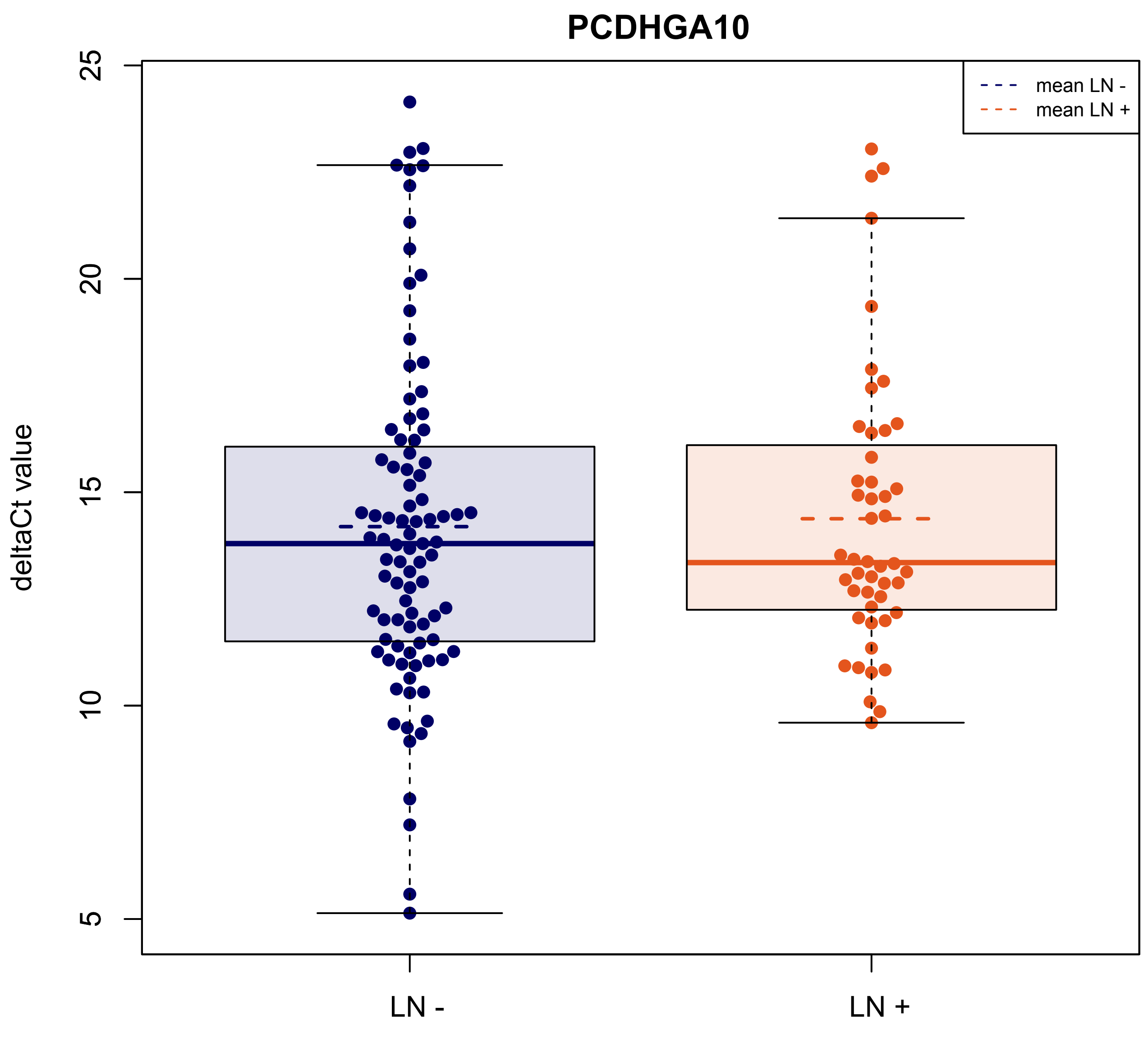

PCDHGA10
deltaCt value
25
20
15
10
5
mean LN -
mean LN +
LN -
LN +

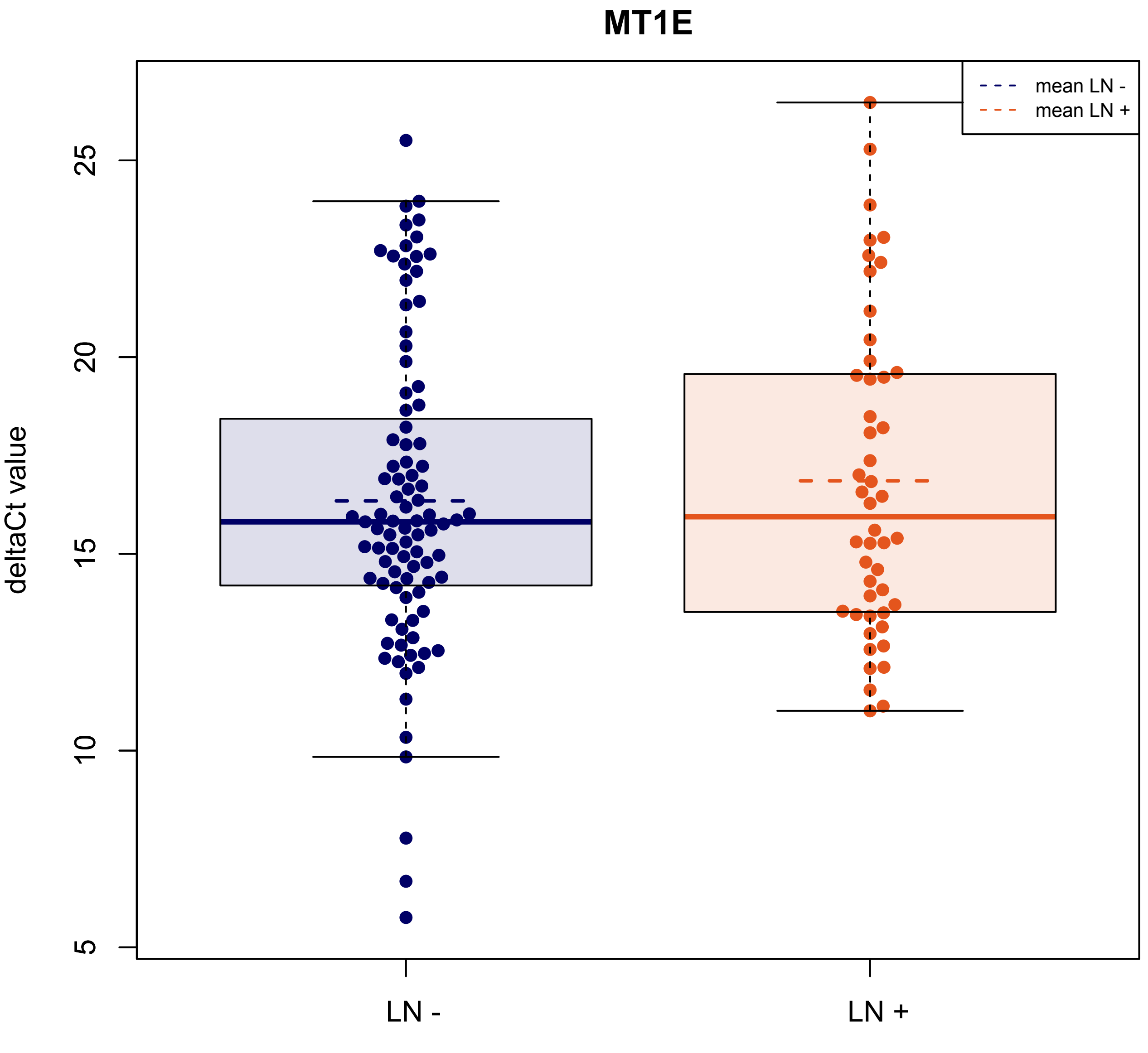

MT1E
mean LN -
mean LN +
deltaCt value
5
10
15
20
25
LN -
LN +

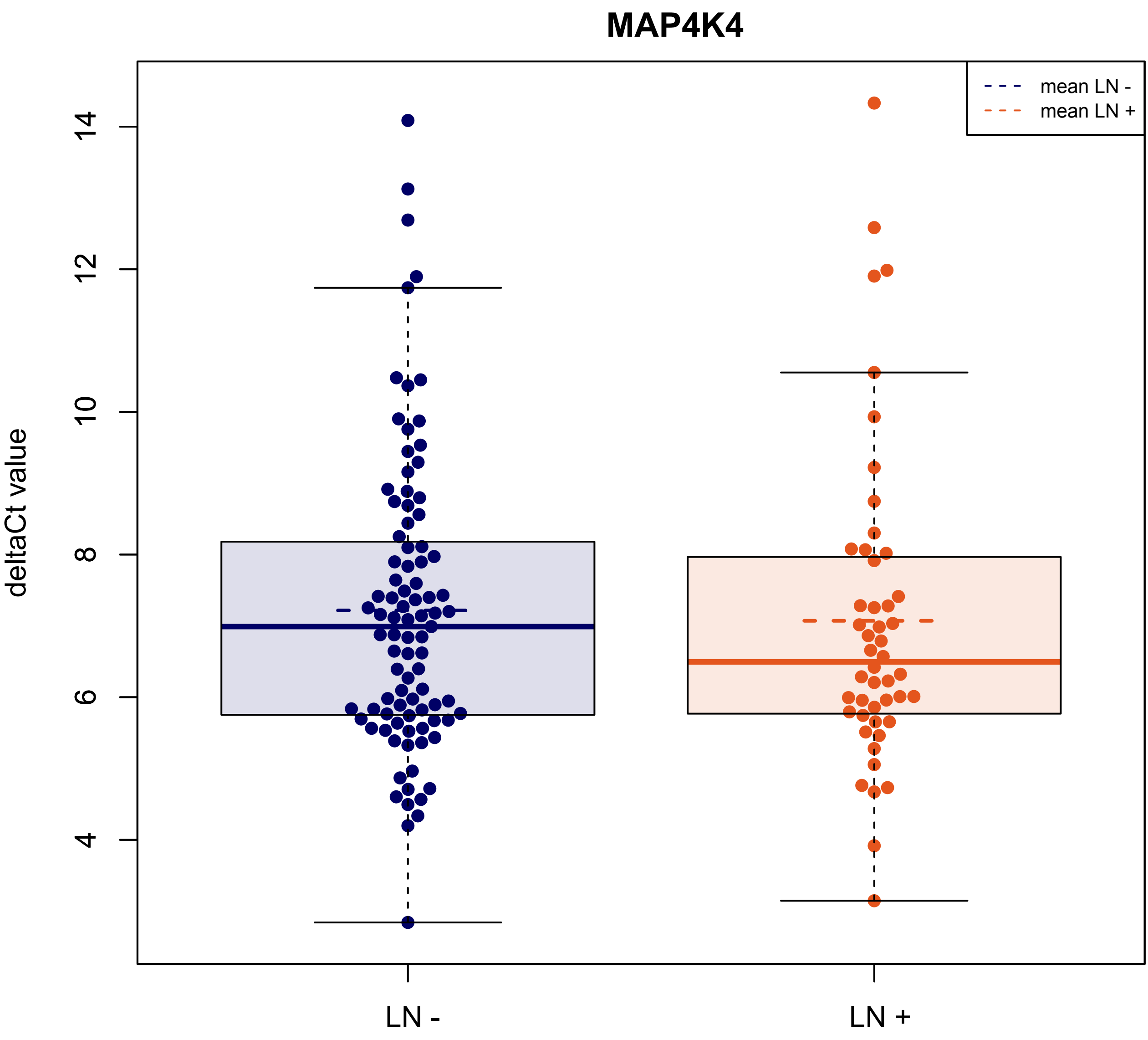

MAP4K4
mean LN -
mean LN +
deltaCt value
14
12
10
8
6
4
LN -
LN +

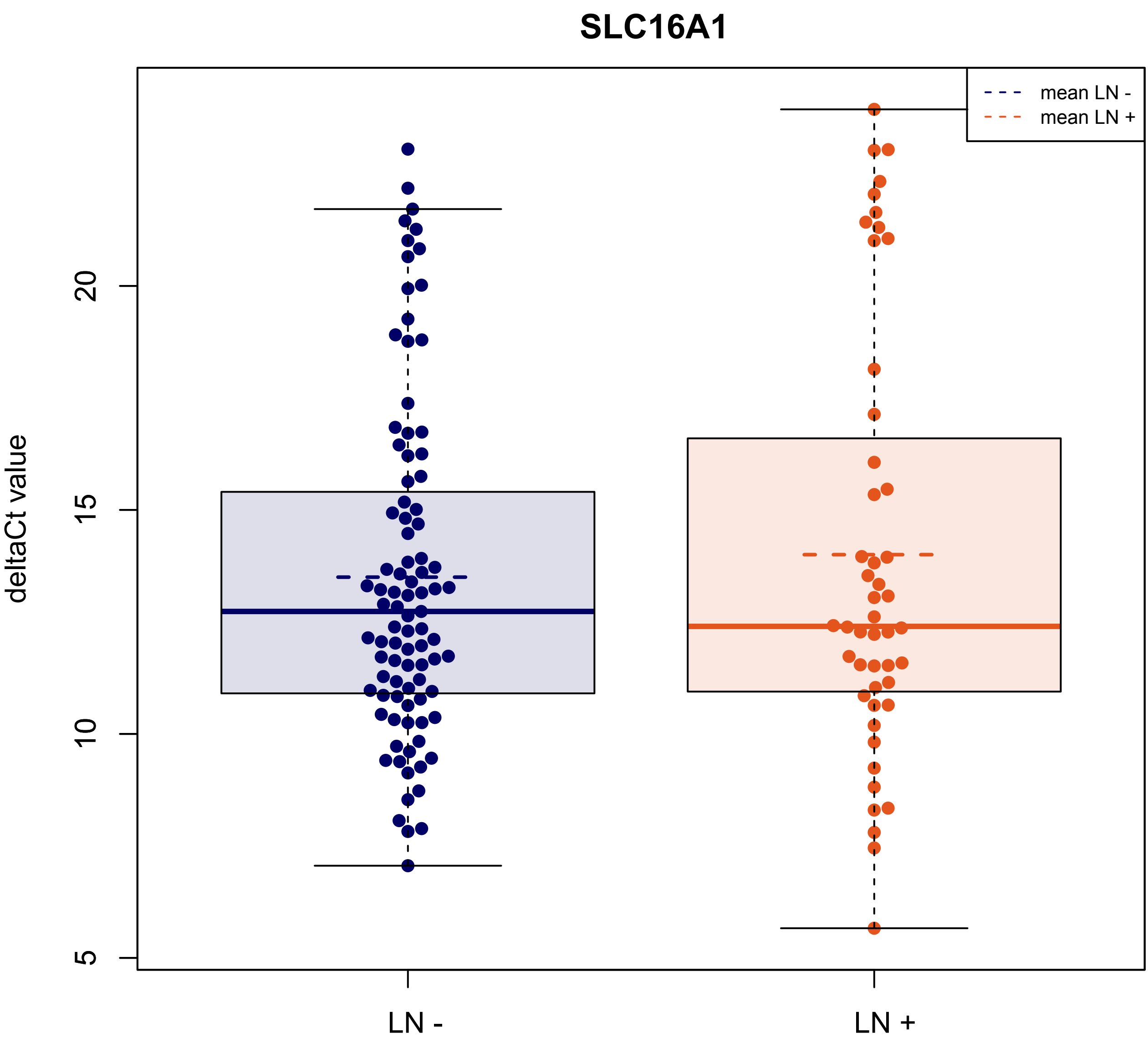

SLC16A1
mean LN -
mean LN +
deltaCt value
5
10
15
20
LN -
LN +

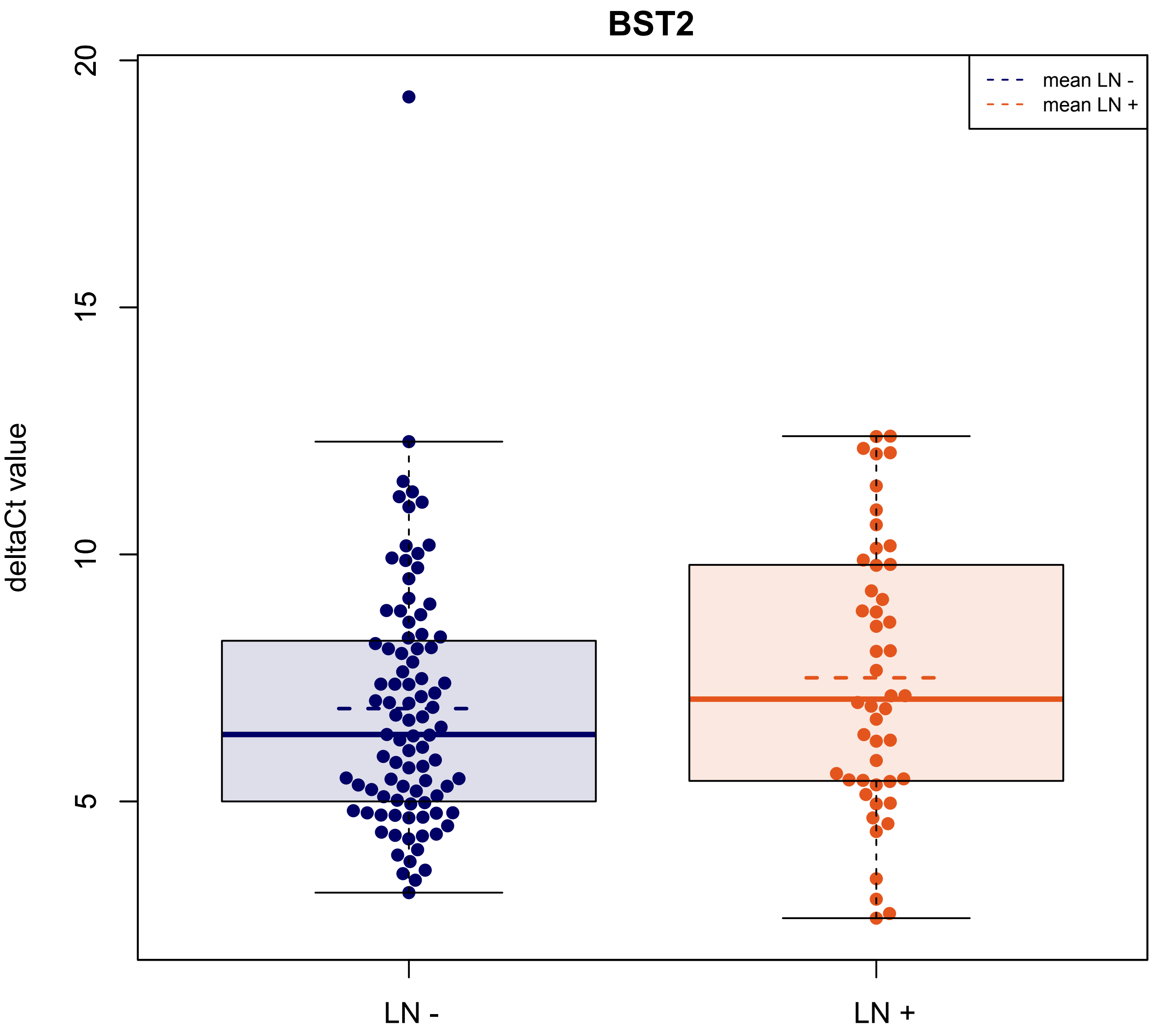
BST2
deltaCt value
20
15
10
5
mean LN -
mean LN +
LN -
LN +

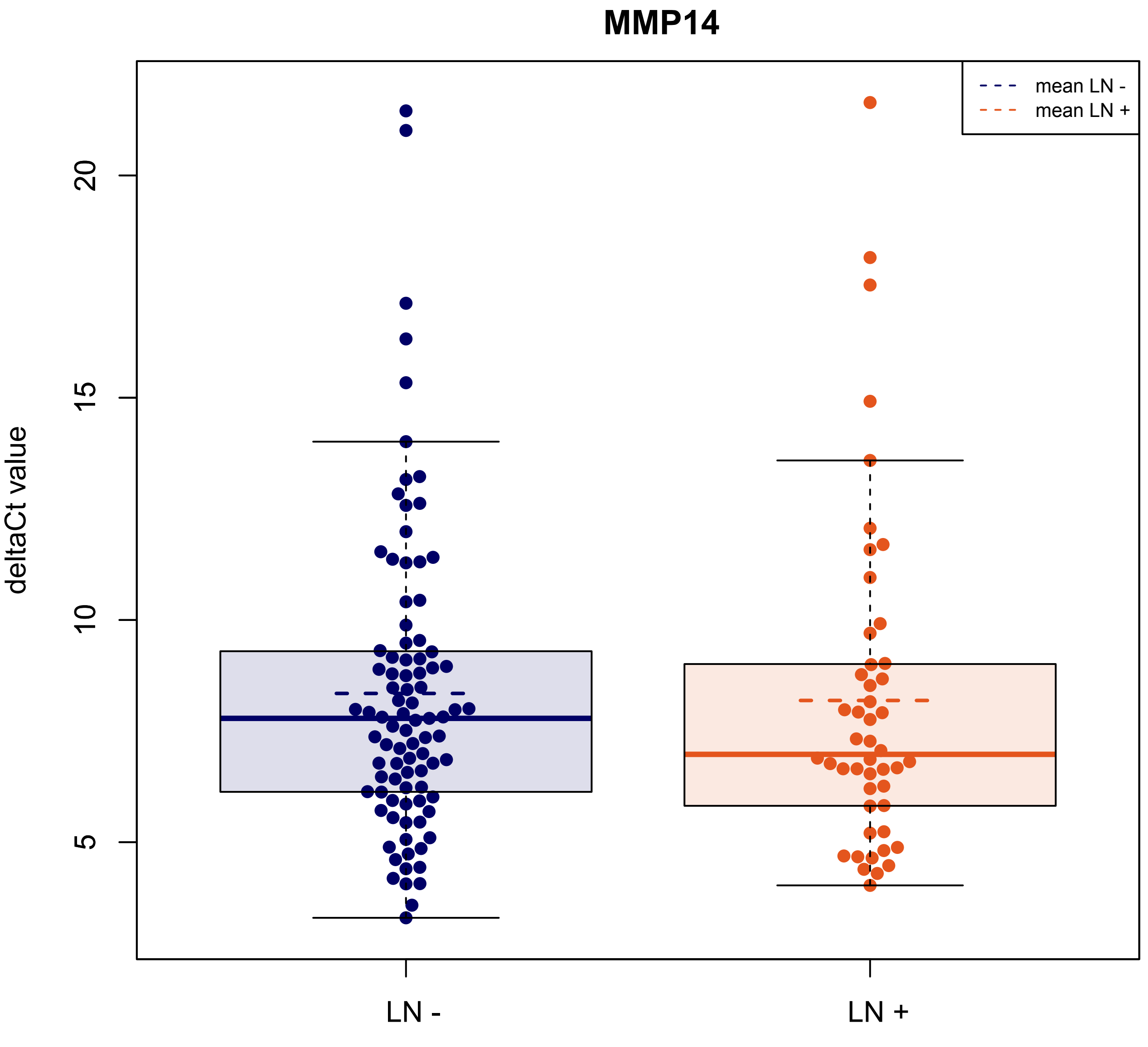
MMP14
mean LN -
mean LN +
deltaCt value
5
10
15
20
LN -
LN +

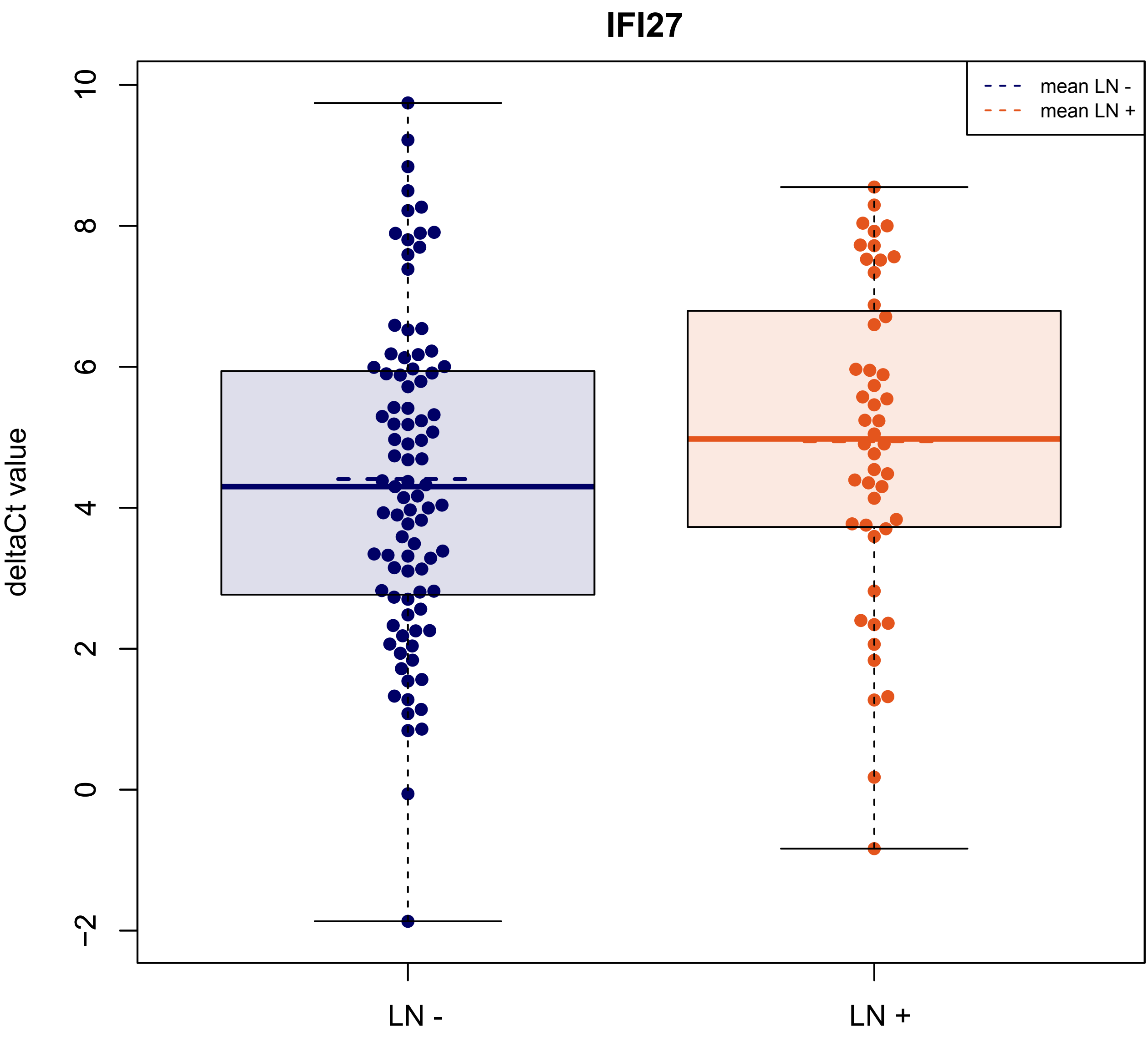

IFI27
mean LN -
mean LN +
deltaCt value
10
8
6
4
2
0
−2
LN -
LN +

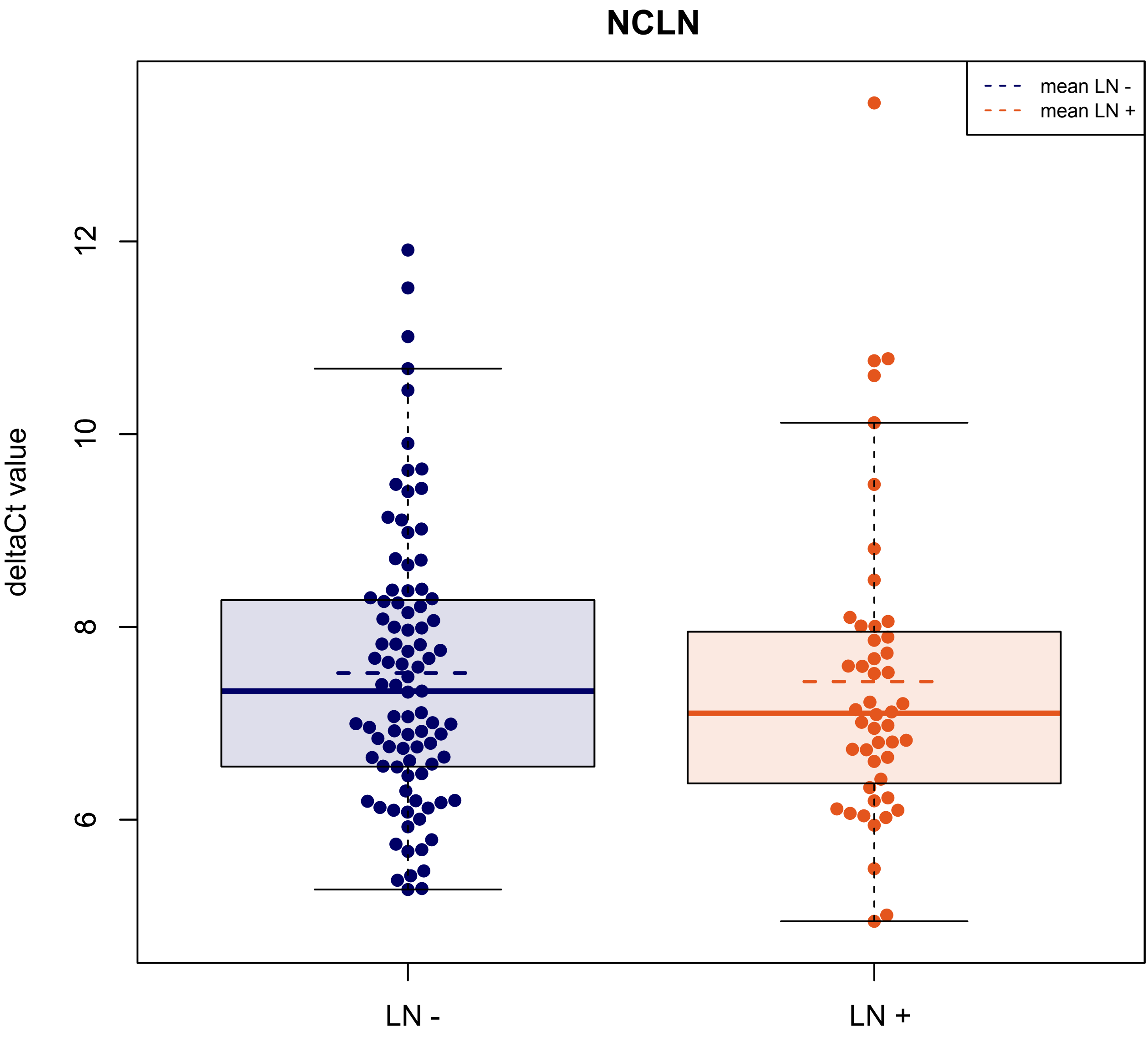

NCLN
mean LN -
mean LN +
deltaCt value
12
10
8
6
LN -
LN +

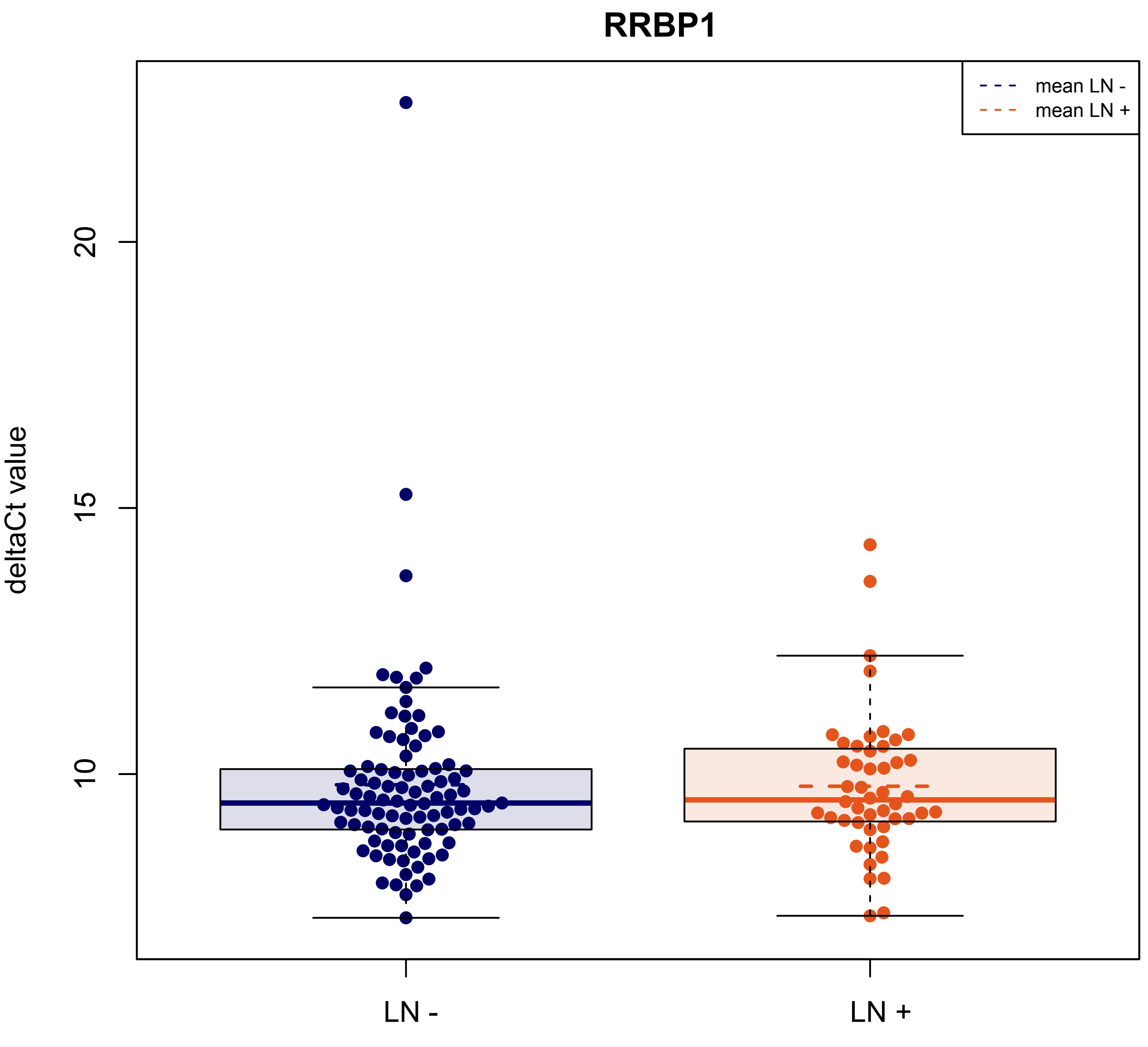
RRBP1
mean LN -
mean LN +
deltaCt value
20
15
10
LN -
LN +

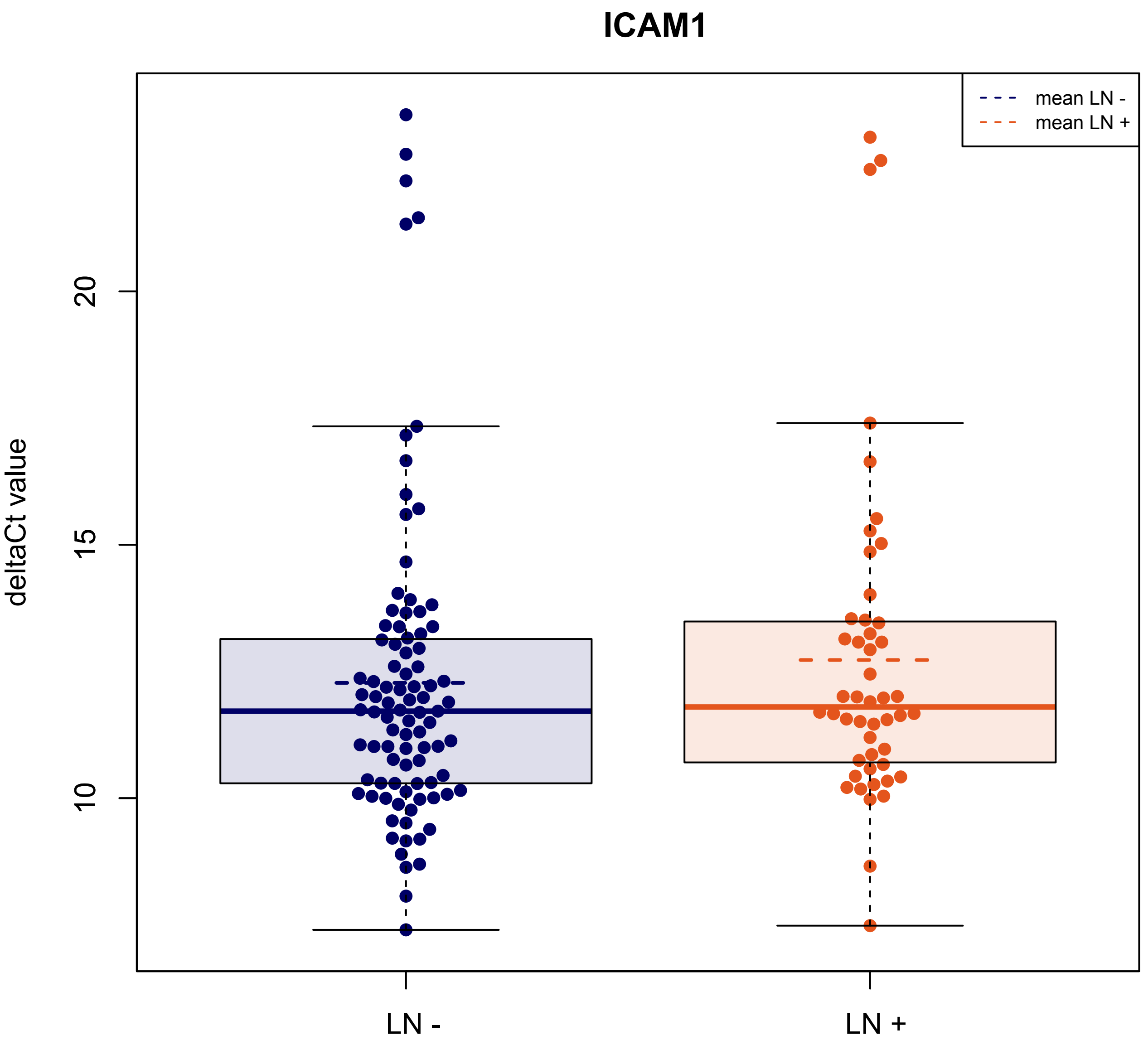
ICAM1
mean LN -
mean LN +
deltaCt value
20
15
10
LN -
LN +

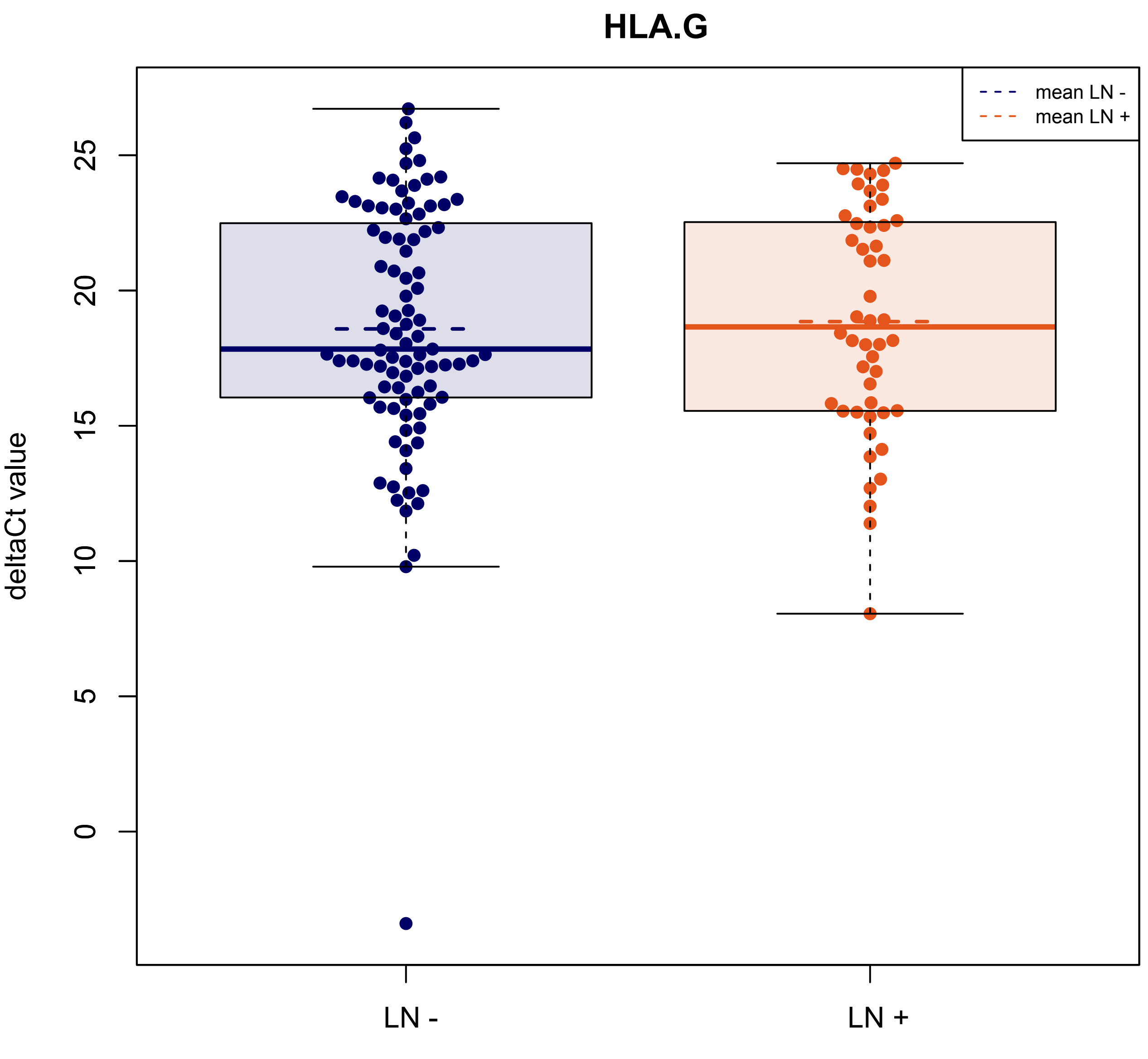

HLA.G
mean LN -
mean LN +
deltaCt value
0
5
10
15
20
25
LN -
LN +

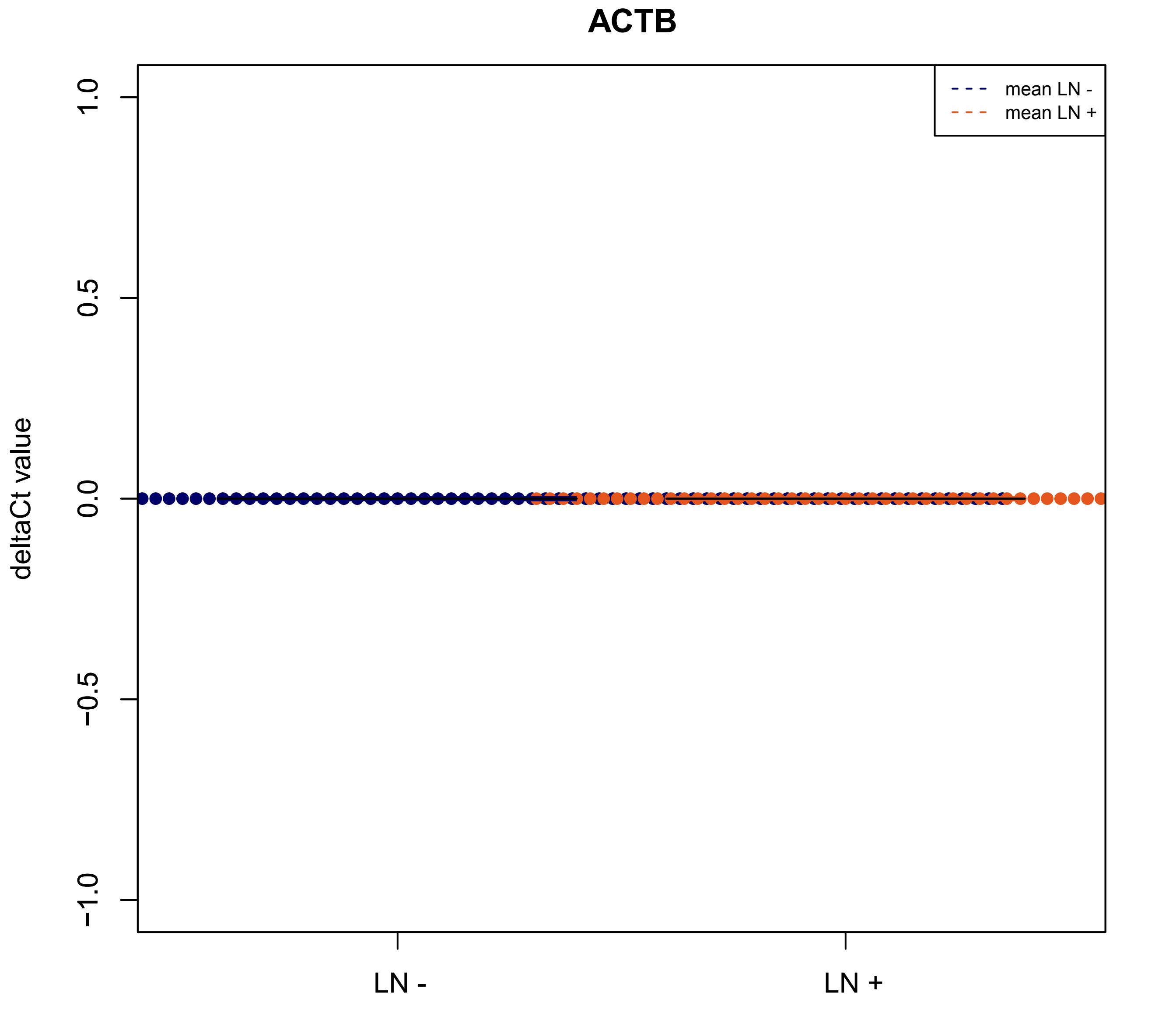
ACTB
mean LN -
mean LN +
deltaCt value
1.0
0.5
0.0
−0.5
−1.0
LN -
LN +

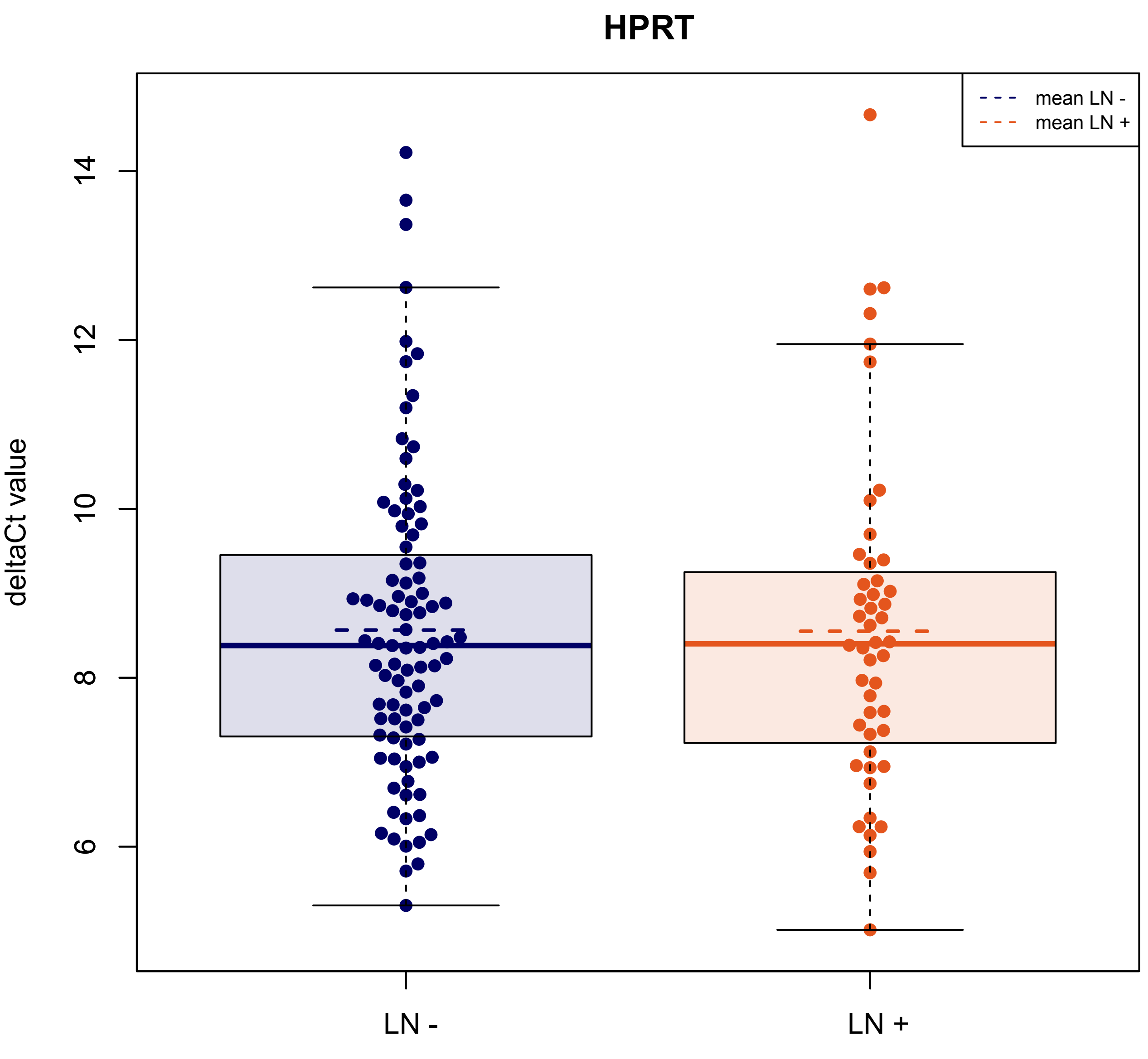

HPRT
mean LN -
mean LN +
deltaCt value
14
12
10
8
6
LN -
LN +
