# Supplementary material for: A reported 20-gene expression signature to predict lymph node-positive disease at radical cystectomy for muscle-invasive bladder cancer is clinically not applicable
Source: PLoS One. 2017 Mar 20;12(3):e0174039. doi: 10.1371/journal.pone.0174039 (PMC5358850; doi:10.1371/journal.pone.0174039)
Supplement: S4 Fig — The box indicates the upper and lower quartiles of distribution, with the solid line indicating the median and the dotted line indicating the mean ΔCt value. (PDF) [file pone.0174039.s004.pdf]

TCGA TOX3 N= 365
Mann−Whitney U p-value 0.71

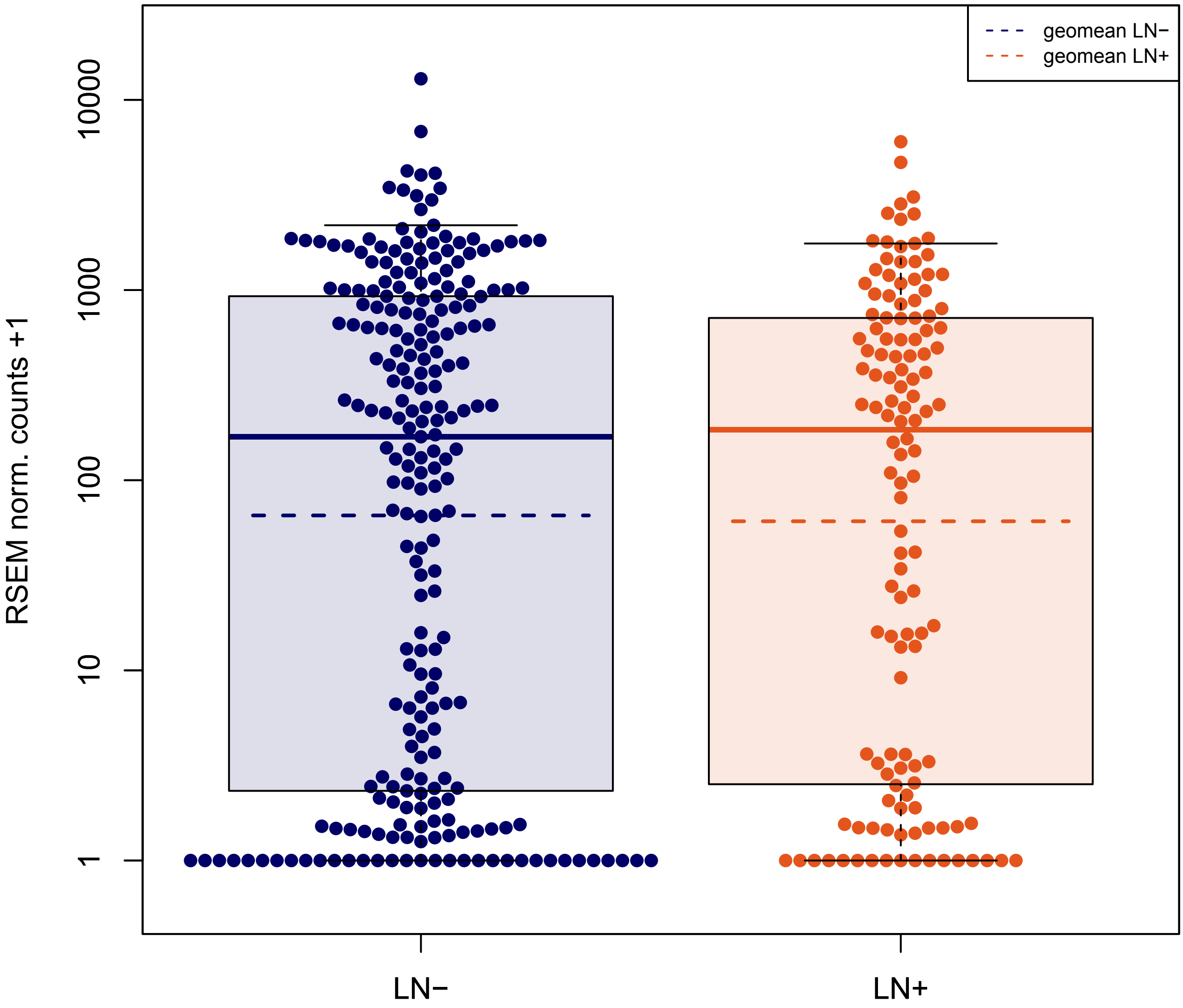

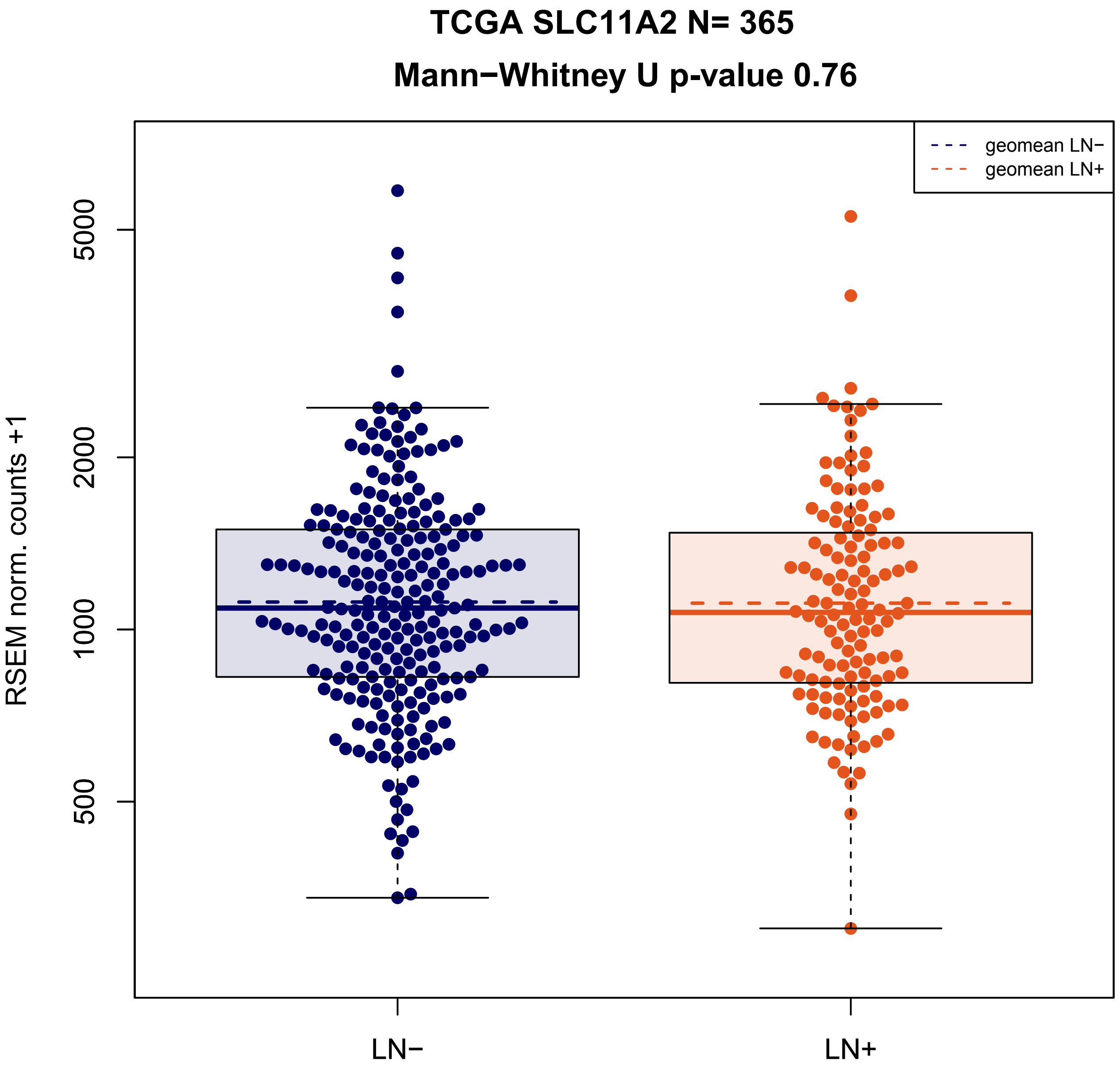

TCGA SLC11A2 N= 365
Mann−Whitney U p-value 0.76
geomean LN−
geomean LN+
RSEM norm. counts +1
5000
2000
1000
500
LN−
LN+

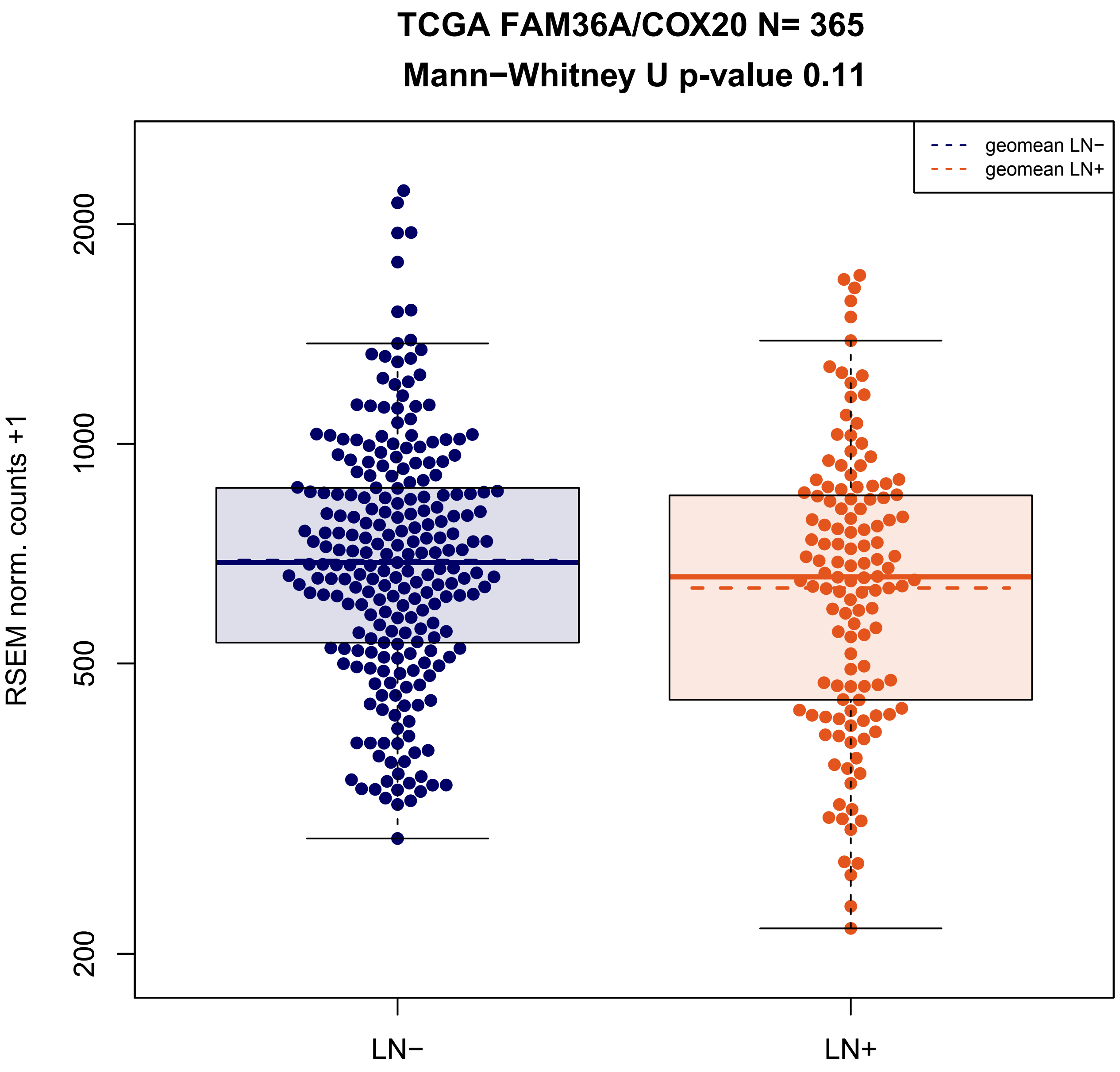
TCGA FAM36A/COX20 N= 365
Mann−Whitney U p-value 0.11
geomean LN−
geomean LN+
RSEM norm. counts +1
2000
1000
500
200
LN−
LN+

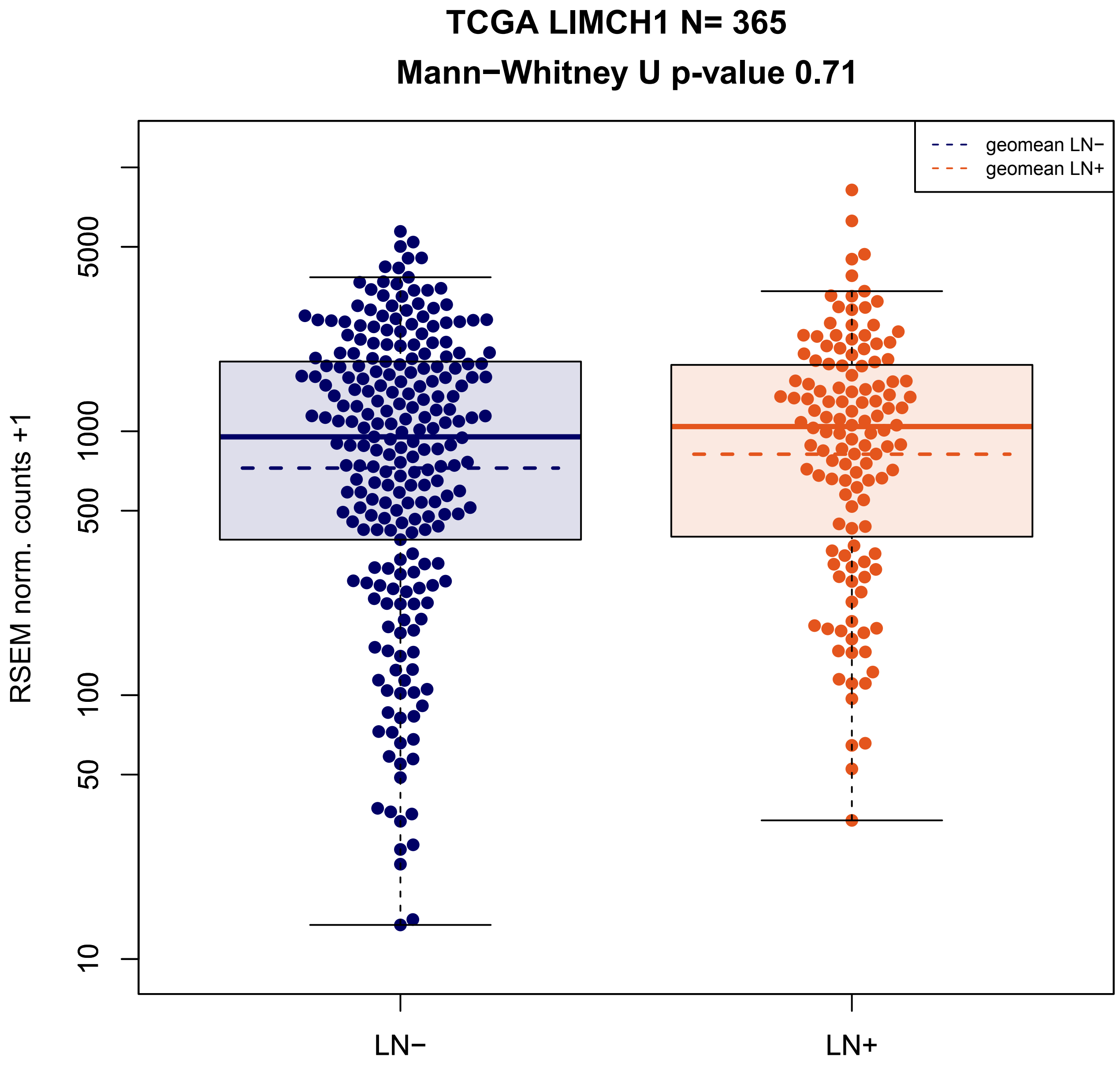
TCGA LIMCH1 N= 365
Mann−Whitney U p-value 0.71
geomean LN−
geomean LN+
RSEM norm. counts +1
10
50
100
500
1000
5000
LN−
LN+

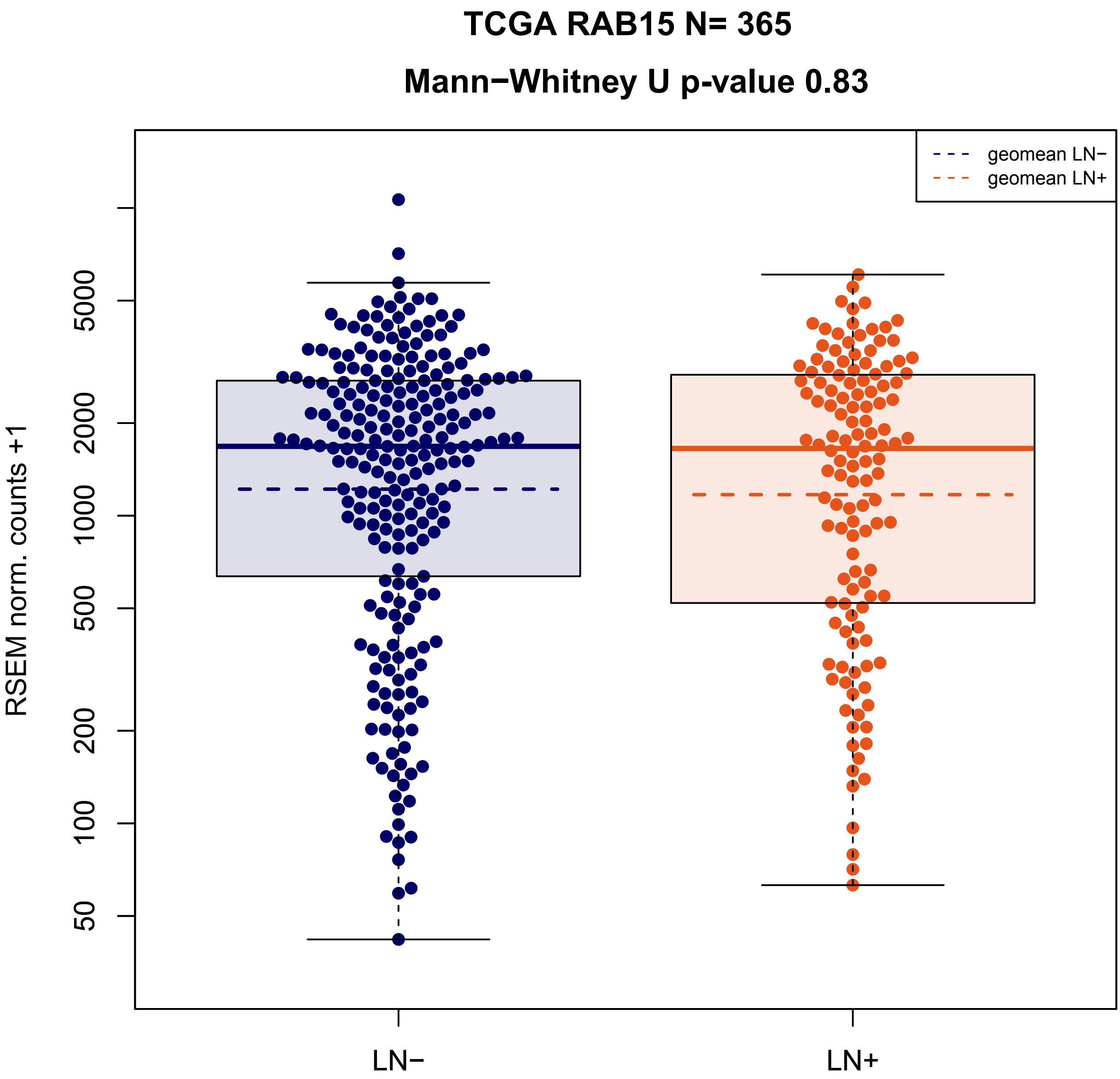
TCGA RAB15 N= 365
Mann−Whitney U p-value 0.83
geomean LN−
geomean LN+
RSEM norm. counts +1
50
100
200
500
1000
2000
5000
LN−
LN+

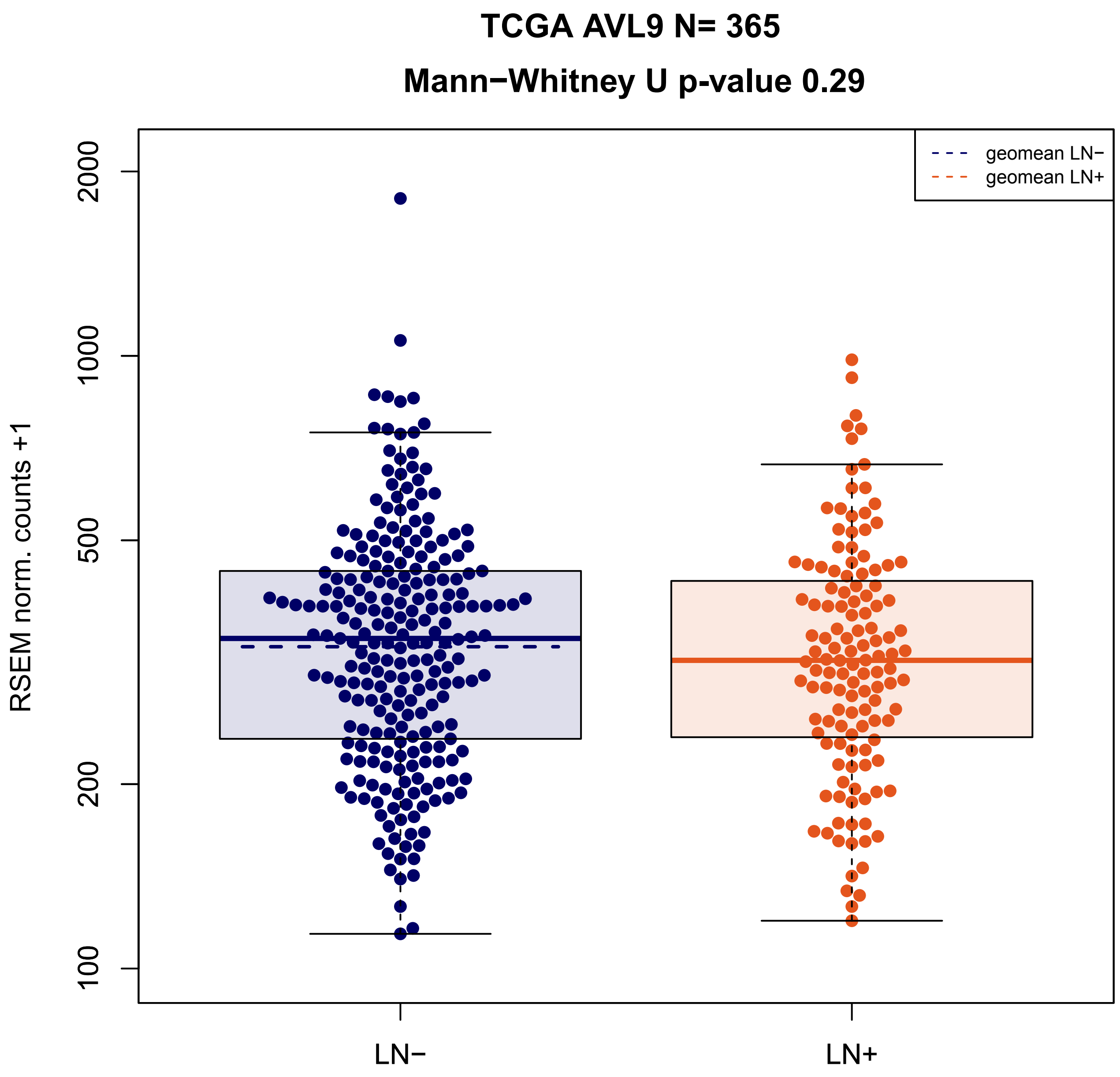
TCGA AVL9 N= 365
Mann−Whitney U p-value 0.29
geomean LN−
geomean LN+
RSEM norm. counts +1
2000
1000
500
200
100
LN−
LN+

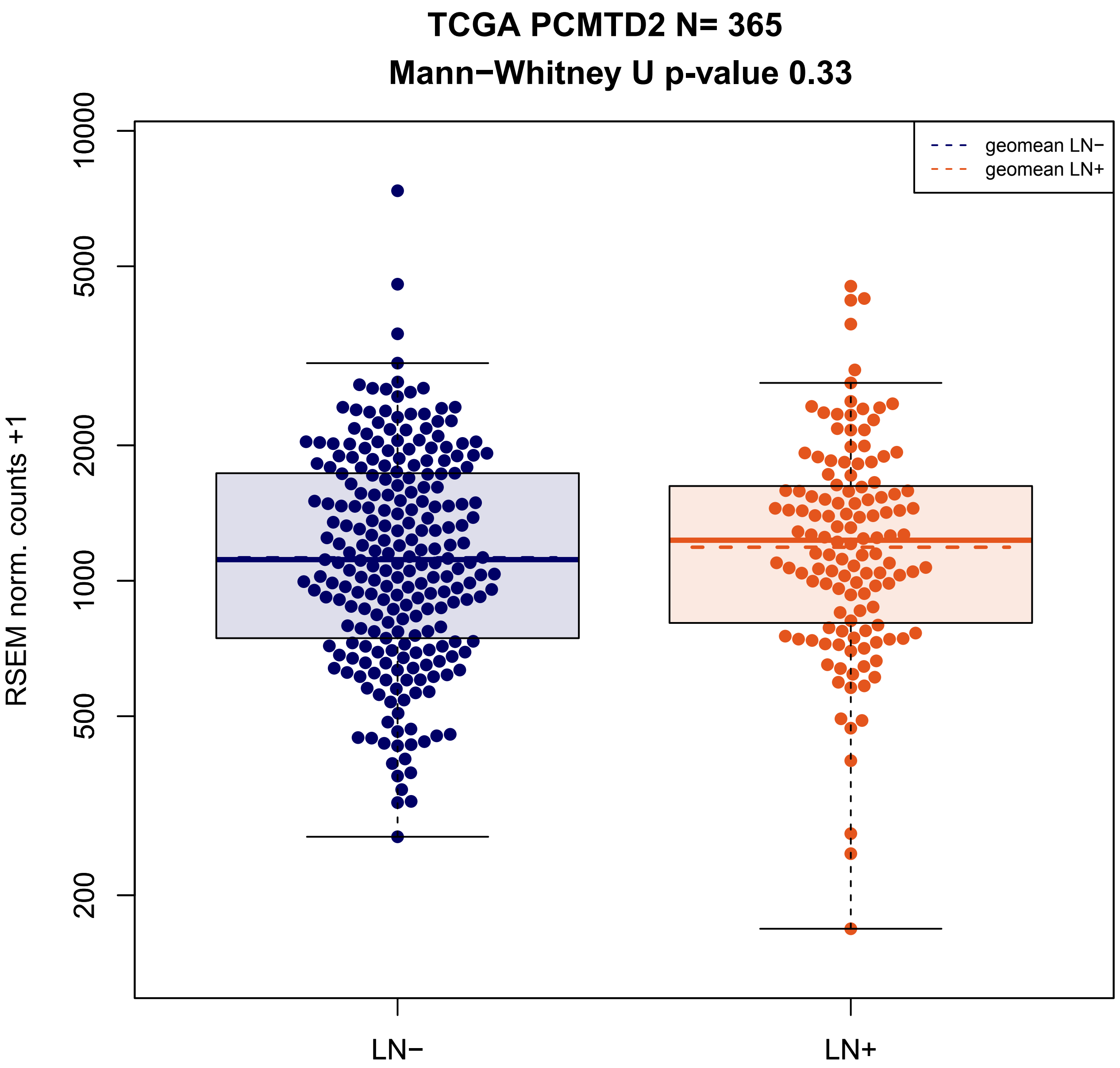

TCGA PCMTD2 N= 365
Mann−Whitney U p-value 0.33
geomean LN−
geomean LN+
RSEM norm. counts +1
10000
5000
2000
1000
500
200
LN−
LN+

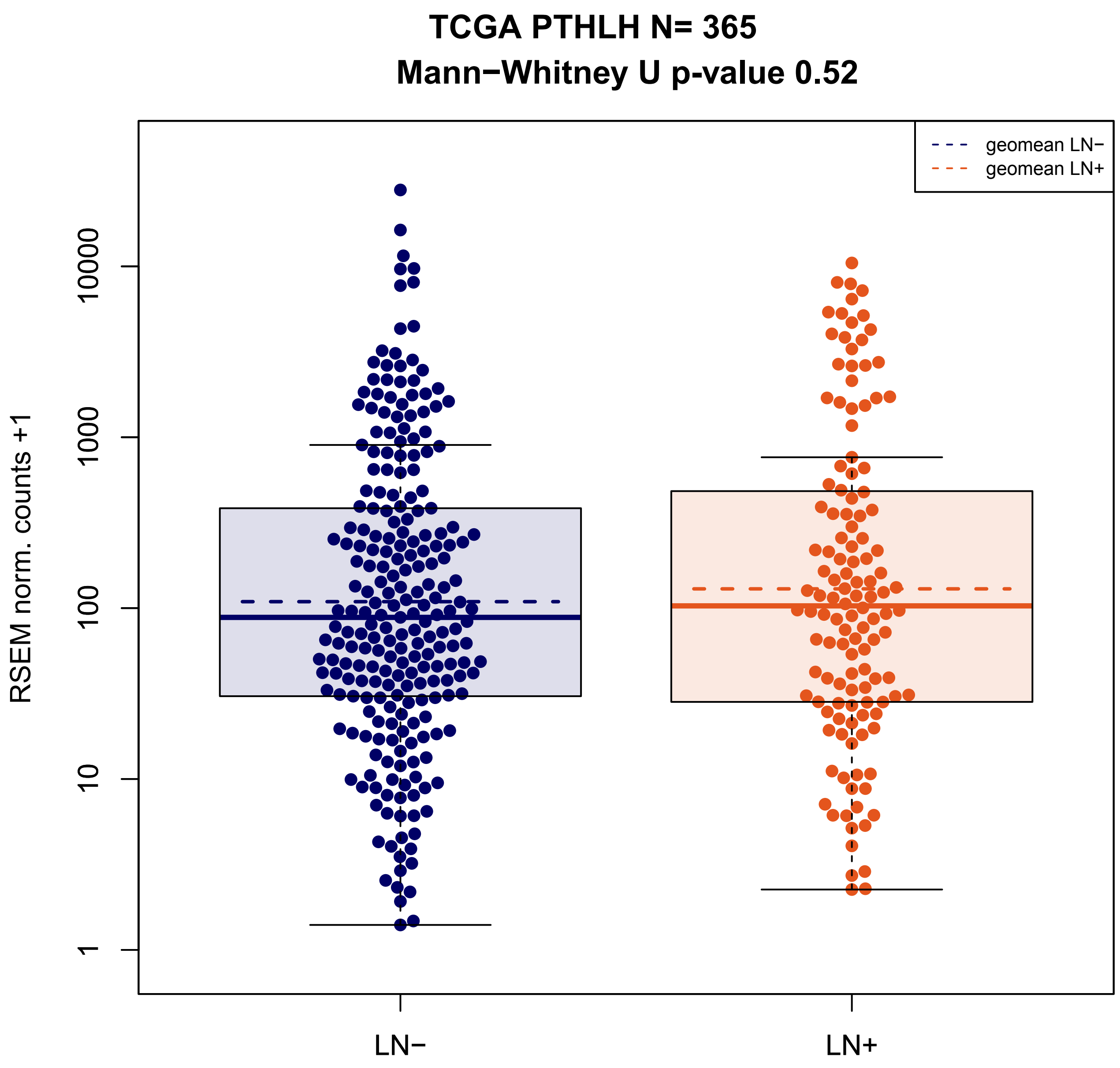
TCGA PTHLH N= 365
Mann−Whitney U p-value 0.52
geomean LN−
geomean LN+
RSEM norm. counts +1
1
10
100
1000
10000
LN−
LN+

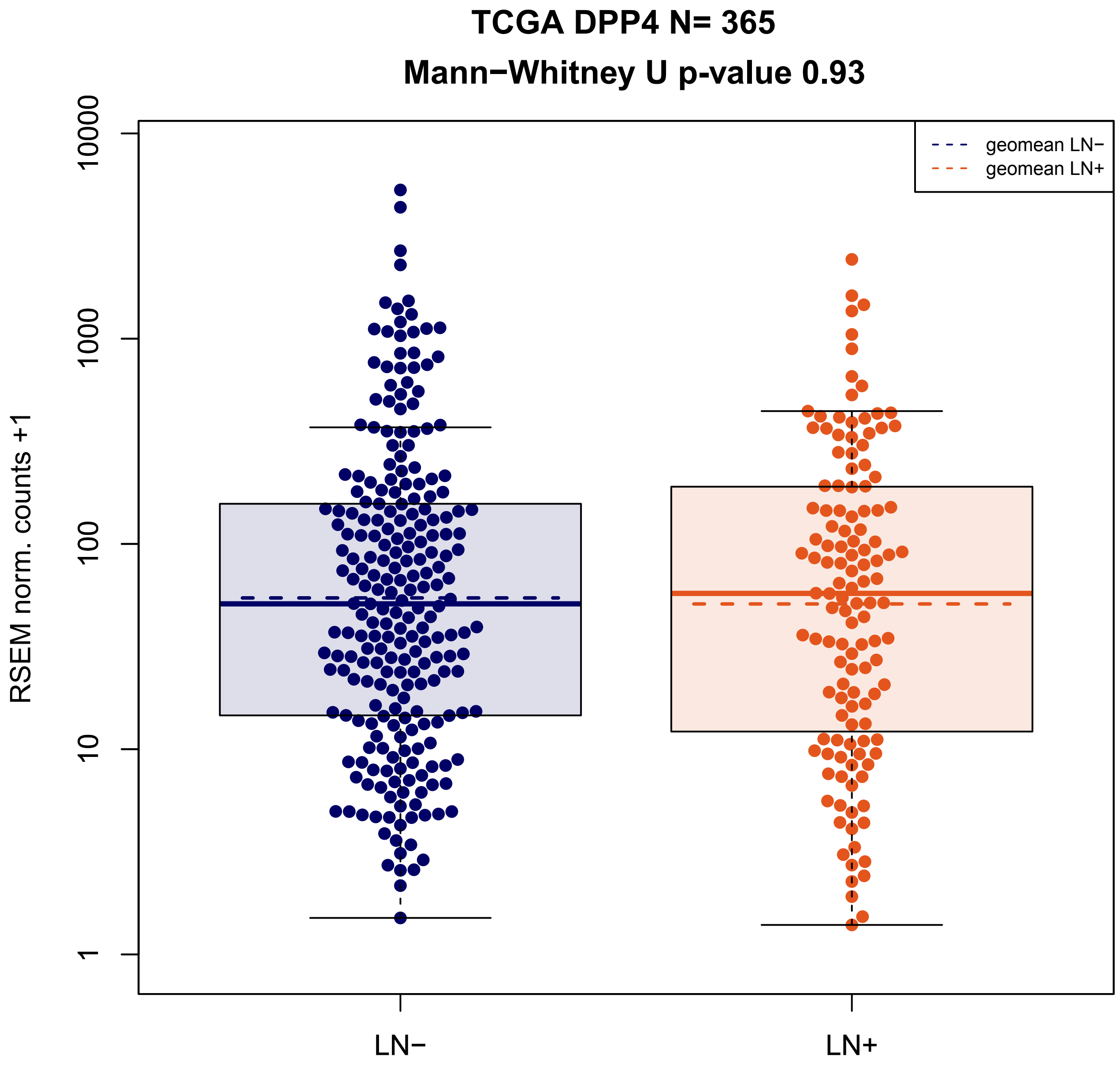
TCGA DPP4 N= 365
Mann−Whitney U p-value 0.93
RSEM norm. counts +1
10000
1000
100
10
1
geomean LN−
geomean LN+
LN−
LN+

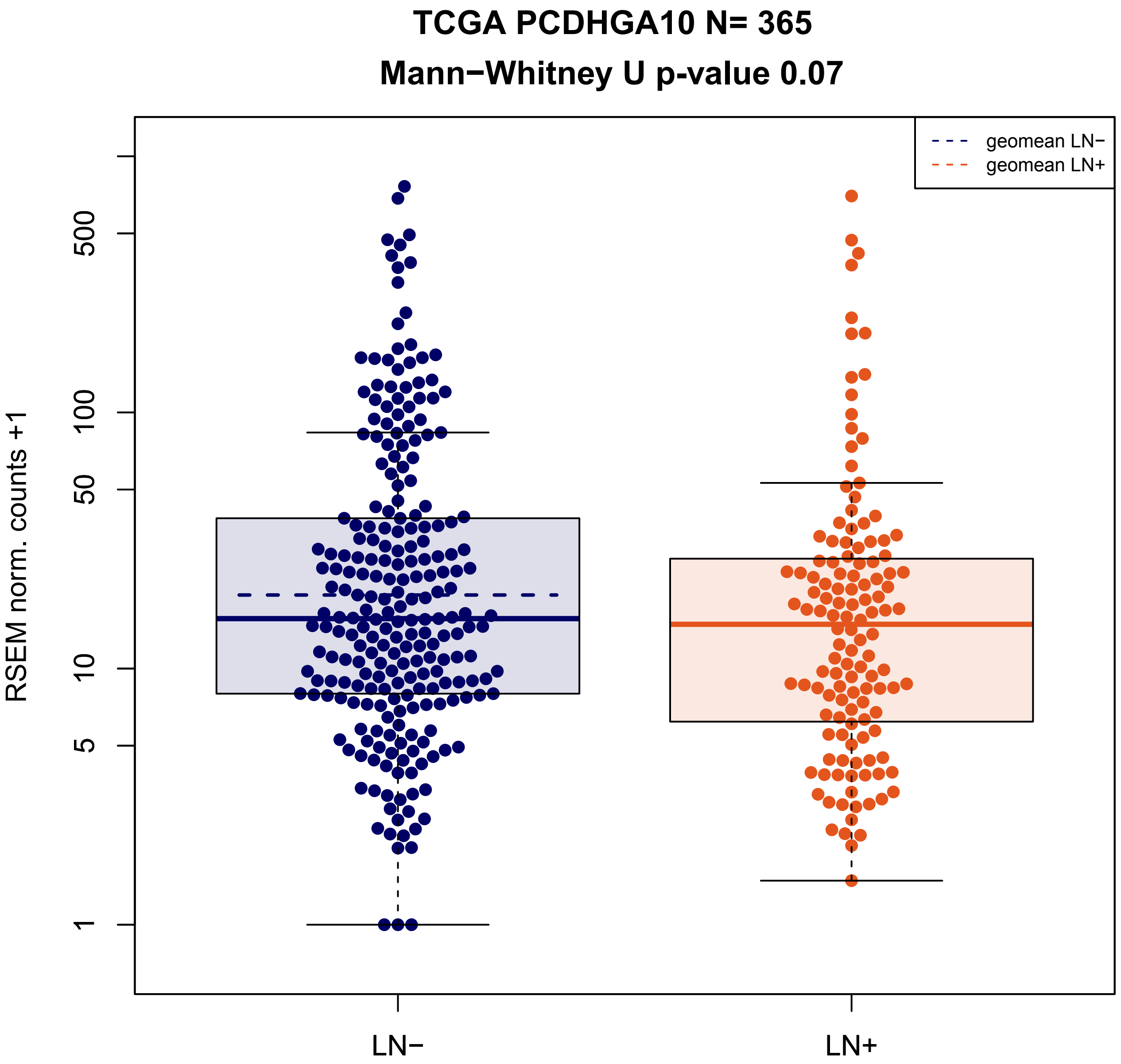

TCGA PCDHGA10 N= 365
Mann−Whitney U p-value 0.07
geomean LN−
geomean LN+
RSEM norm. counts +1
1
5
10
50
100
500
LN−
LN+

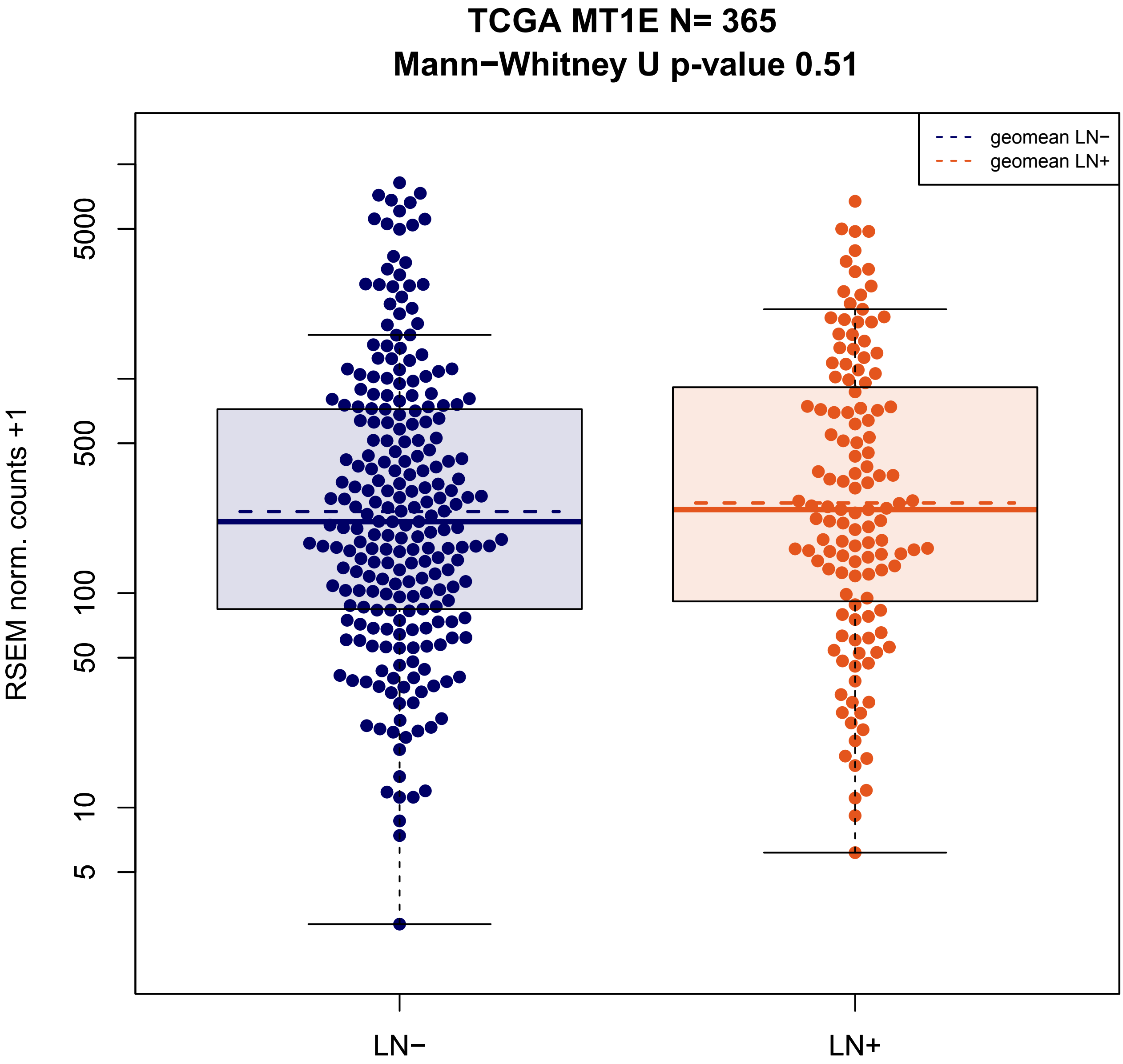

TCGA MT1E N= 365
Mann−Whitney U p-value 0.51
geomean LN−
geomean LN+
RSEM norm. counts +1
5
10
50
100
500
5000
LN−
LN+

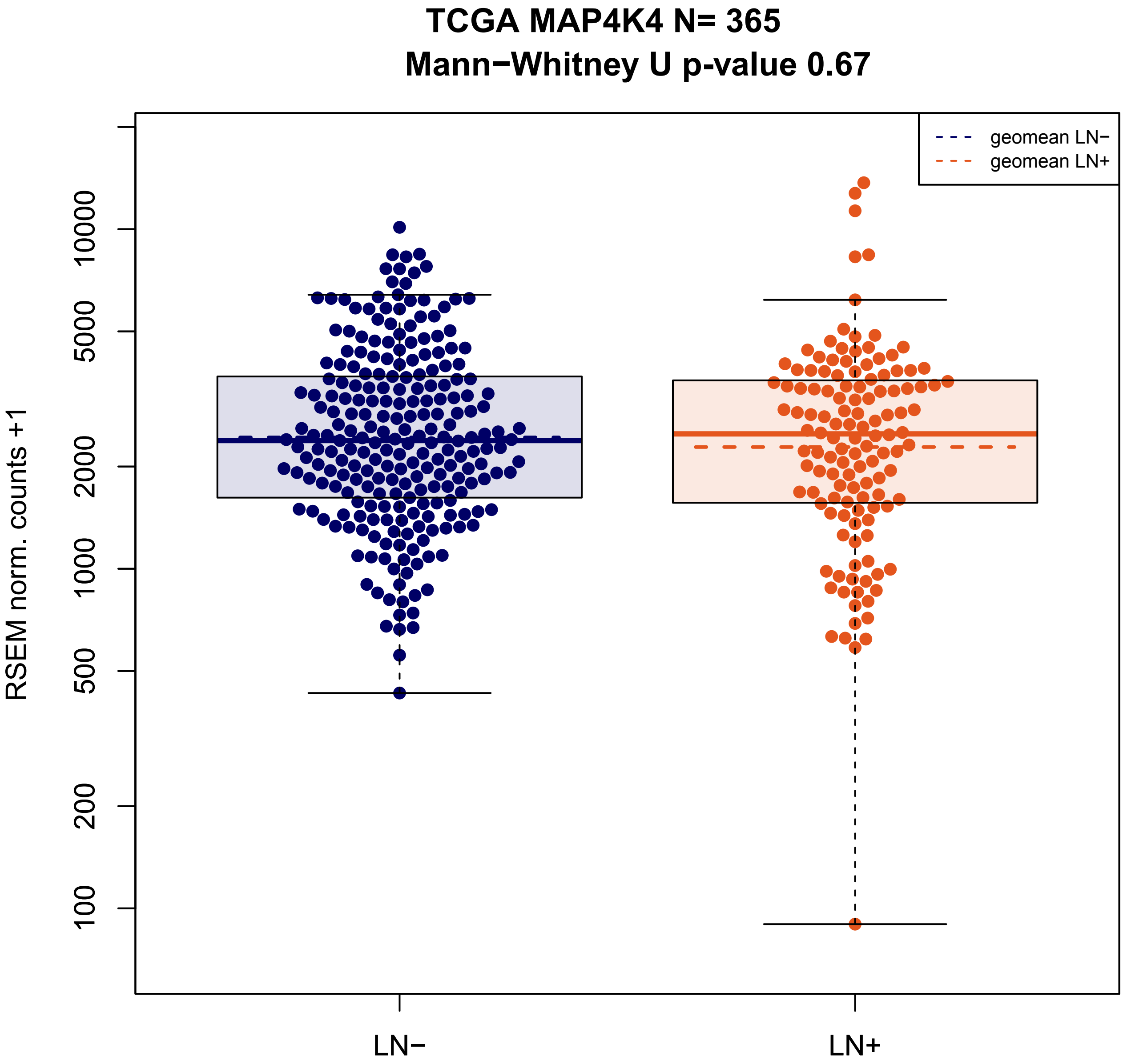

TCGA MAP4K4 N= 365
Mann−Whitney U p-value 0.67
geomean LN−
geomean LN+
RSEM norm. counts +1
100
200
500
1000
2000
5000
10000
LN−
LN+

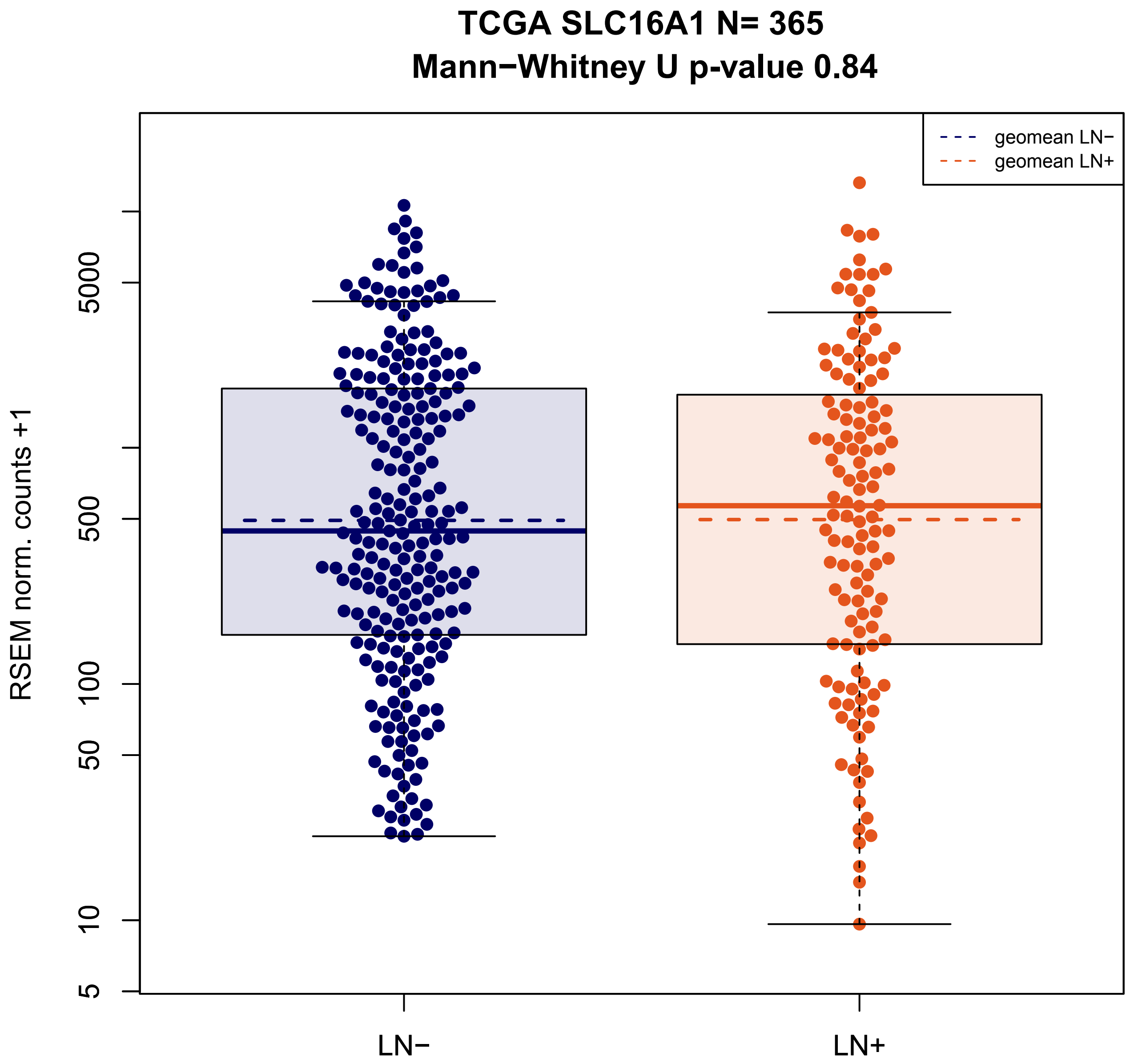

TCGA SLC16A1 N= 365
Mann−Whitney U p-value 0.84
geomean LN−
geomean LN+
RSEM norm. counts +1
5
10
50
100
500
5000
LN−
LN+

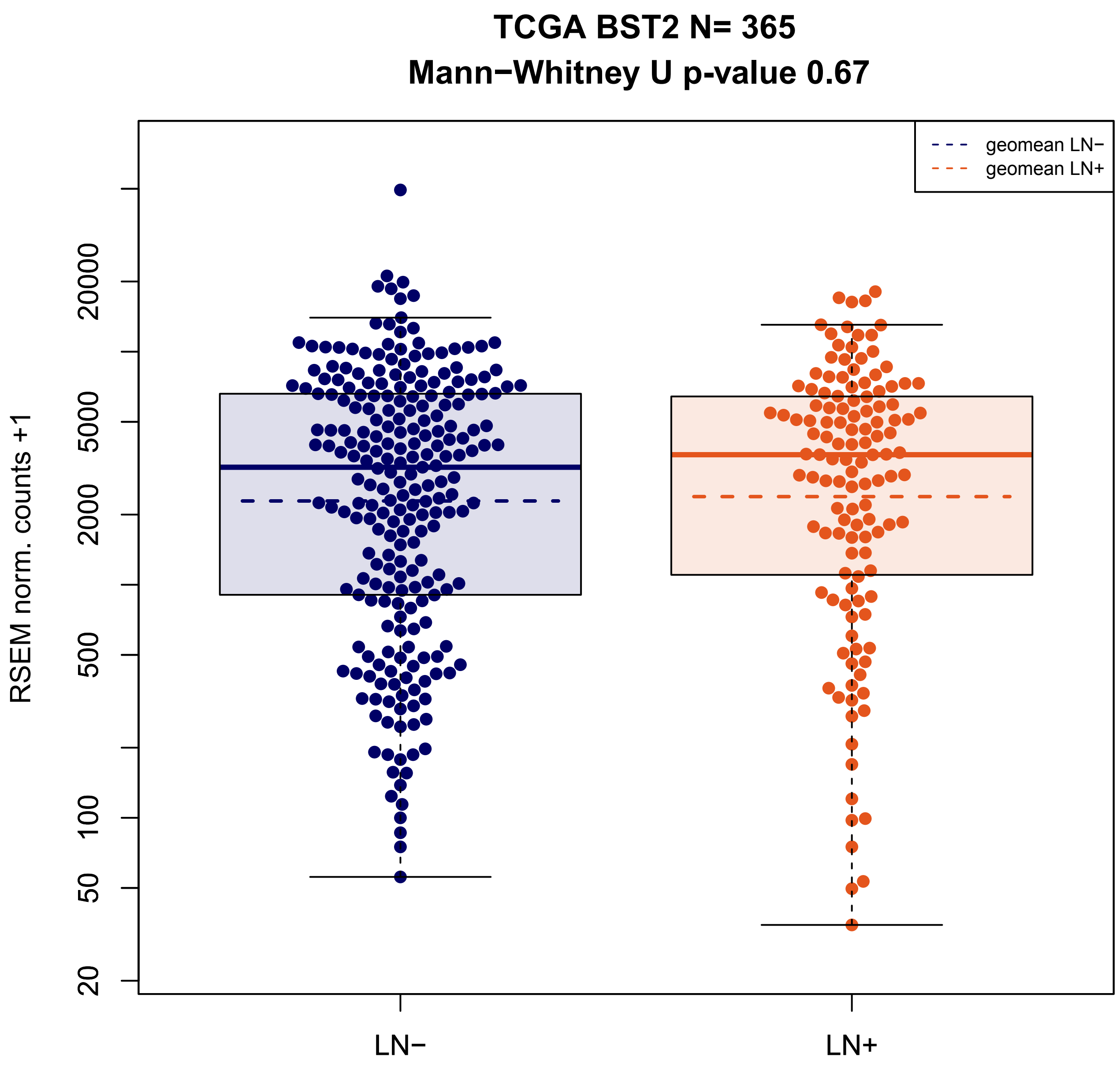
TCGA BST2 N= 365
Mann−Whitney U p-value 0.67
geomean LN−
geomean LN+
RSEM norm. counts +1
20000
5000
2000
500
100
50
20
LN−
LN+

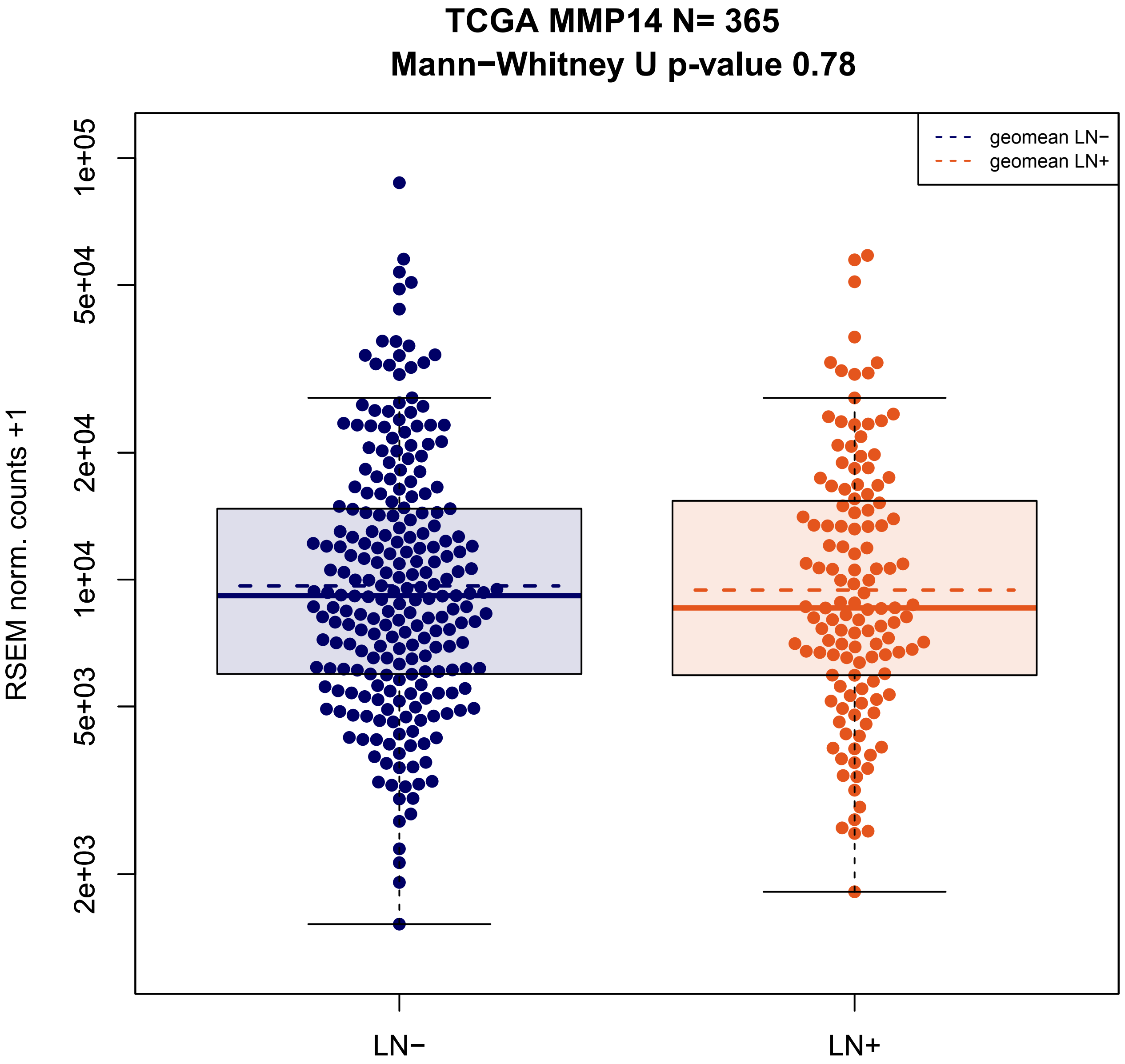
TCGA MMP14 N= 365
Mann−Whitney U p-value 0.78
geomean LN−
geomean LN+
RSEM norm. counts +1
1e+05
5e+04
2e+04
1e+04
5e+03
2e+03
LN−
LN+

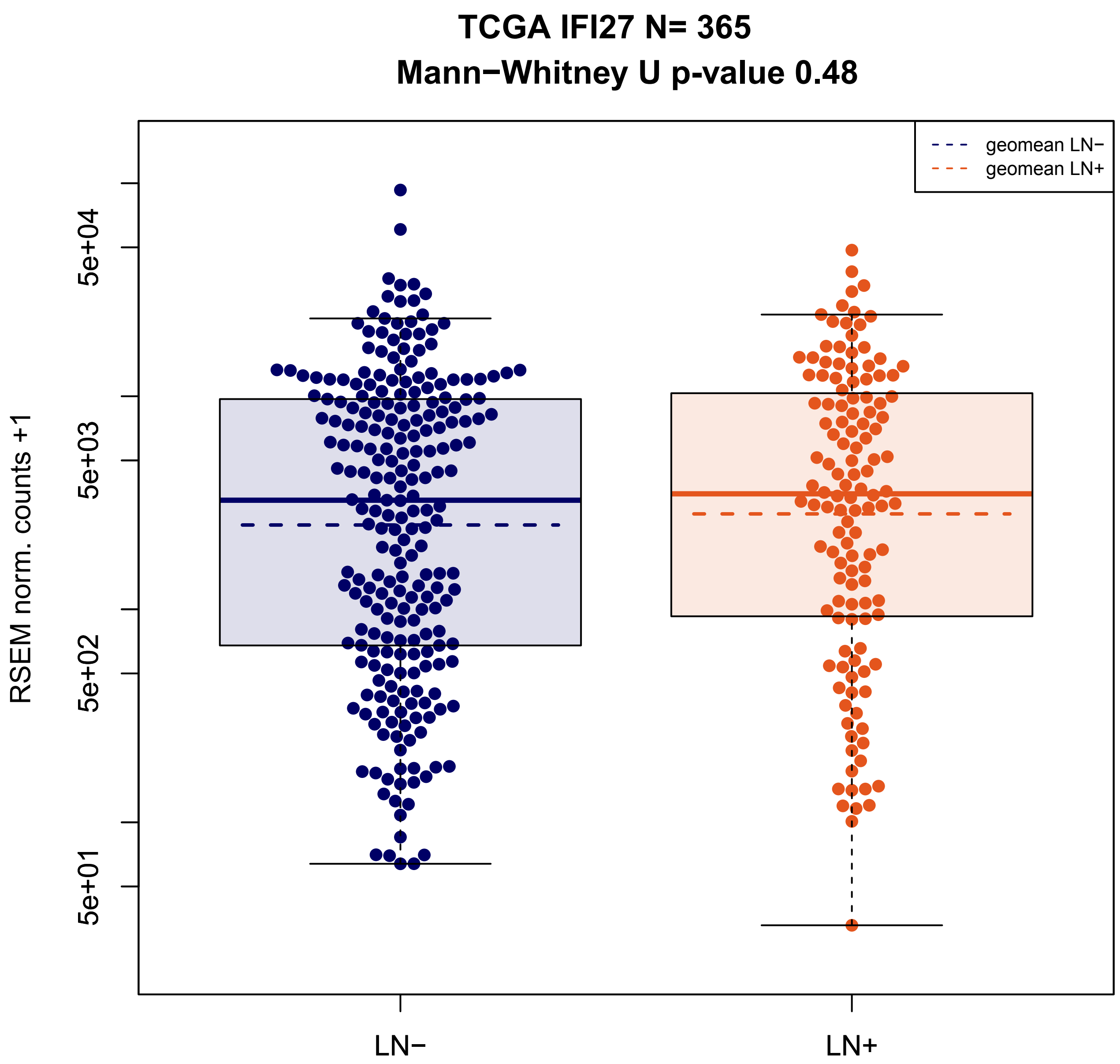
TCGA IFI27 N= 365
Mann−Whitney U p-value 0.48
geomean LN−
geomean LN+
RSEM norm. counts +1
5e+04
5e+03
5e+02
5e+01
LN−
LN+

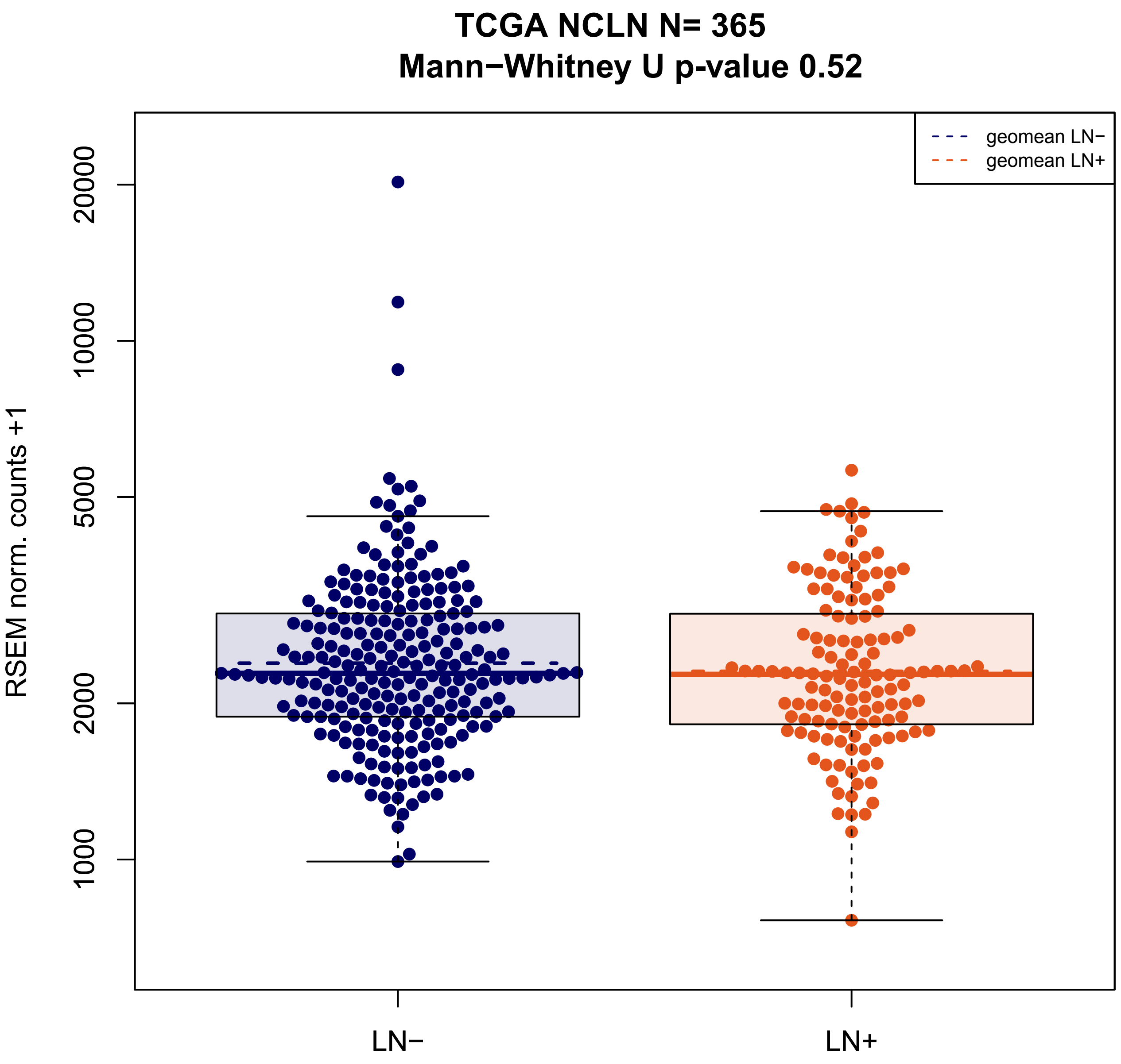
TCGA NCLN N= 365
Mann−Whitney U p-value 0.52
geomean LN−
geomean LN+
RSEM norm. counts +1
20000
10000
5000
2000
1000
LN−
LN+

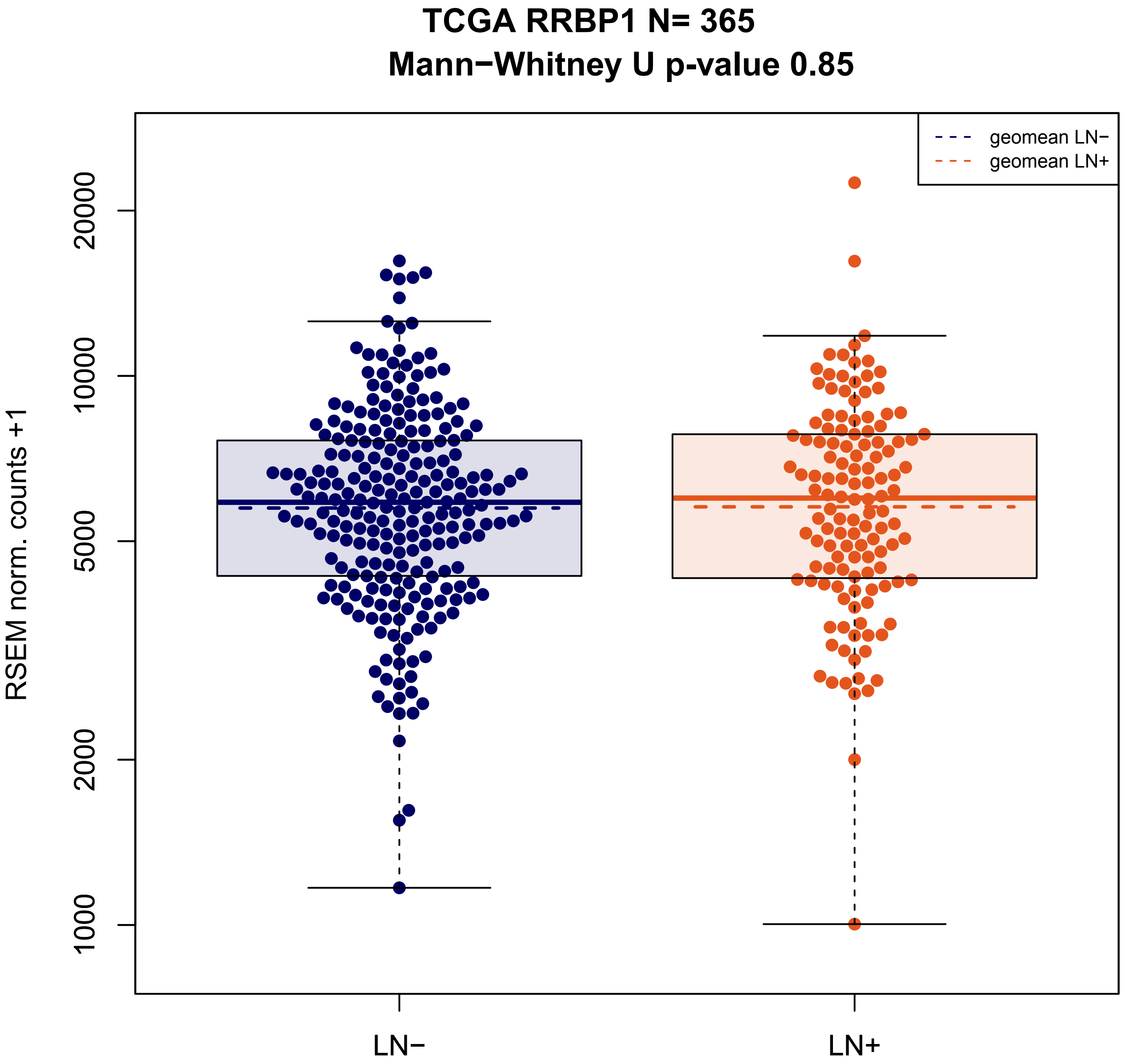
TCGA RRBP1 N= 365
Mann−Whitney U p-value 0.85
geomean LN−
geomean LN+
RSEM norm. counts +1
20000
10000
5000
2000
1000
LN−
LN+

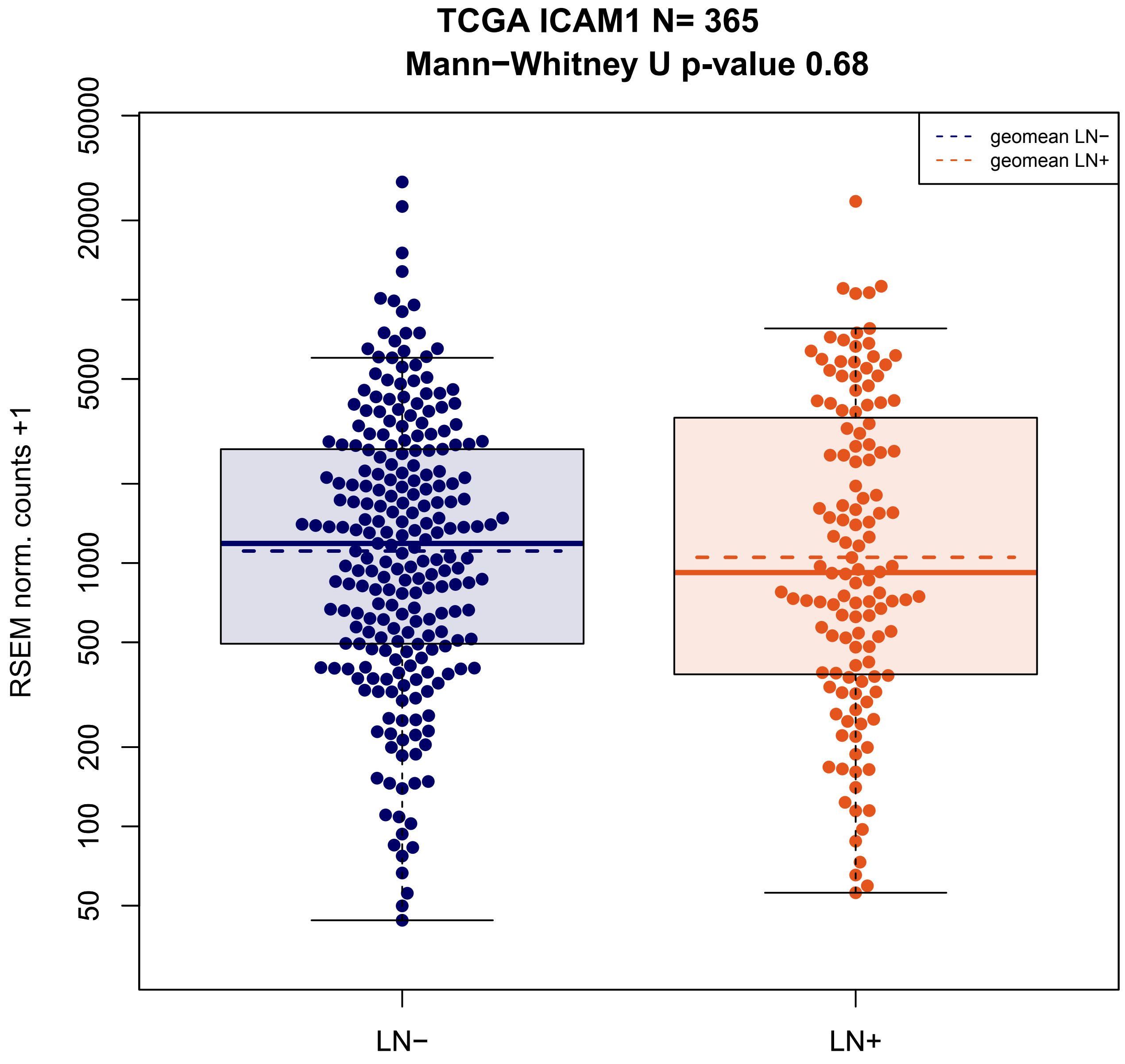

TCGA ICAM1 N= 365
Mann−Whitney U p-value 0.68
RSEM norm. counts +1
50000
20000
5000
1000
500
200
100
50
geomean LN−
geomean LN+
LN−
LN+

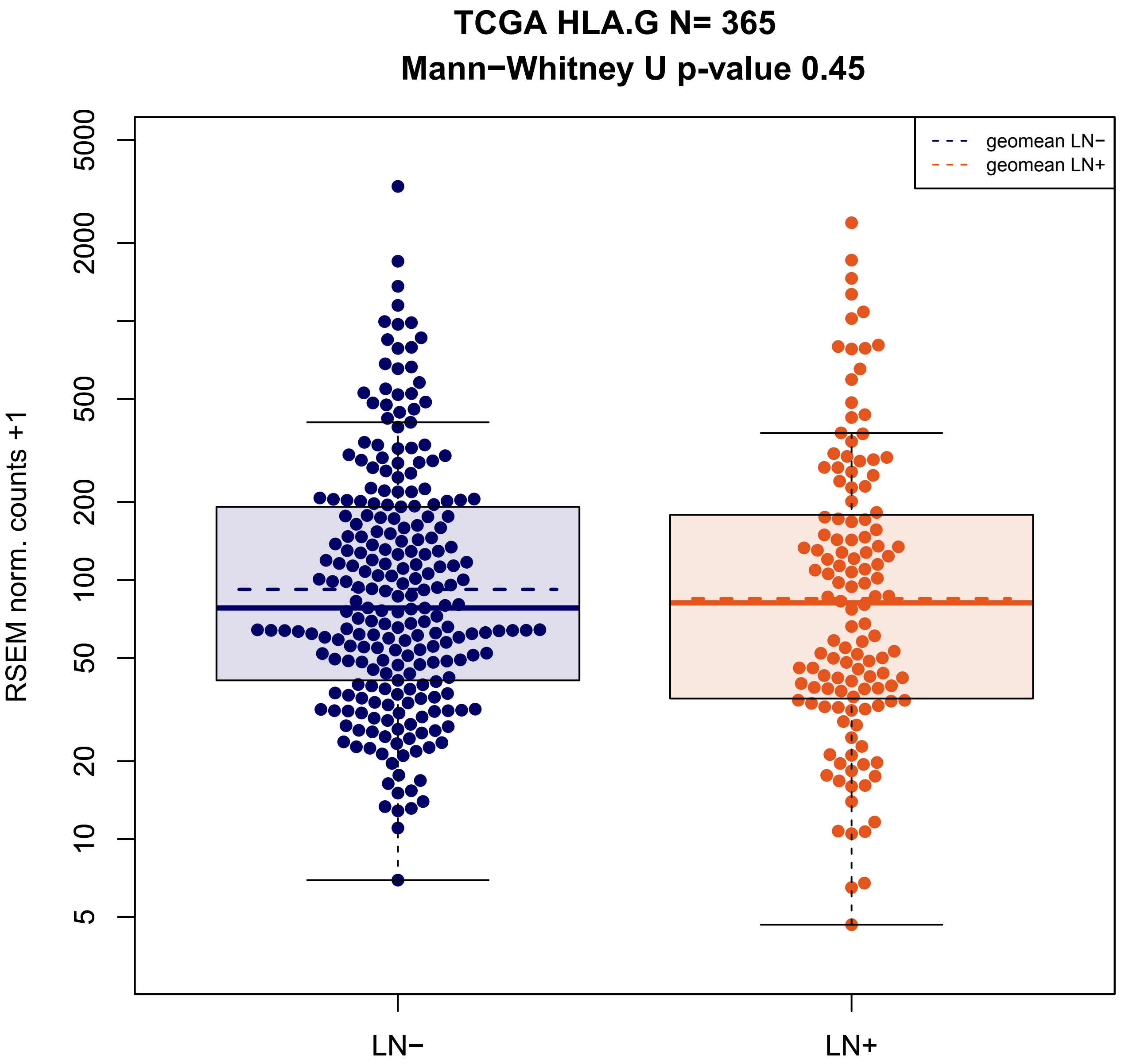

TCGA HLA.G N= 365
Mann−Whitney U p-value 0.45
geomean LN−
geomean LN+
RSEM norm. counts +1
5000
2000
500
200
100
50
20
10
5
LN−
LN+
