# Supplementary figures and images for: A reported 20-gene expression signature to predict lymph node-positive disease at radical cystectomy for muscle-invasive bladder cancer is clinically not applicable
Source: PLoS One. 2017 Mar 20;12(3):e0174039. doi: 10.1371/journal.pone.0174039 (PMC5358850; doi:10.1371/journal.pone.0174039)

corplot N = 408

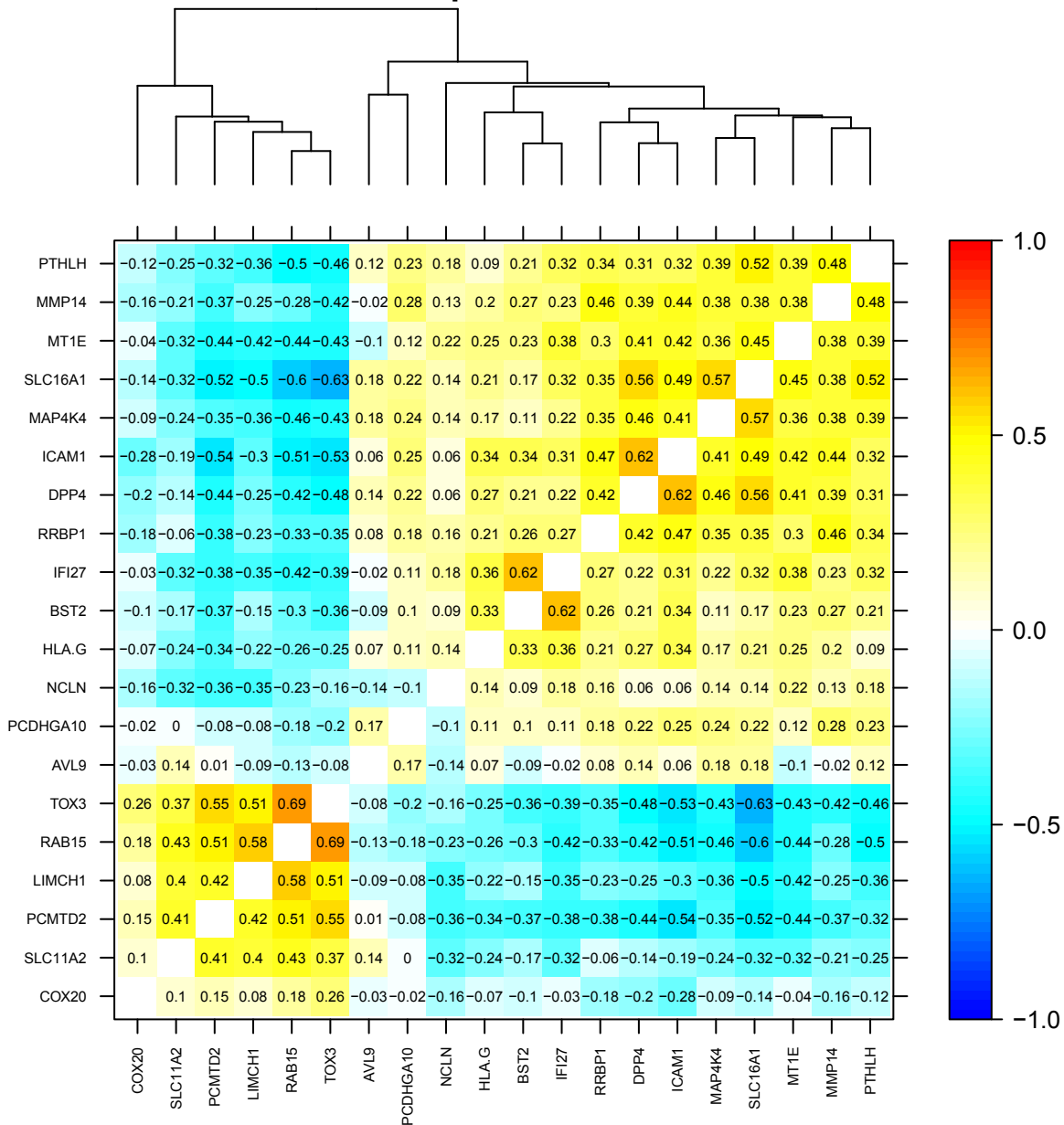

Supplement: S5 Fig — (PDF) [file pone.0174039.s005.pdf]
